# Rh-Catalyzed Cascade C-H Activation/Annulation of *N*-Hydroxybenzamides and Propargylic Acetates for Modular Access to Isoquinolones

**DOI:** 10.3390/molecules27238553

**Published:** 2022-12-05

**Authors:** Taibei Fang, Shiwen Zhang, Qingqing Ye, Shuwen Kong, Tingting Yang, Kaijie Tang, Xinwei He, Yongjia Shang

**Affiliations:** 1 Key Laboratory of Functional Molecular Solids, Ministry of Education, Anhui Laboratory of Molecule-Based Materials (State Key Laboratory Cultivation Base), College of Chemistry and Materials Science, Anhui Normal University, Wuhu 241000, China; 2 Department of Medicine, Chuzhou City Vocation College, Chuzhou 239000, China

**Keywords:** C-H activation, isoquinolones, *N*-hydroxybenzamides, propargylic acetates

## Abstract

A sequential Rh(III)-catalyzed C-H activation/annulation of *N*-hydroxybenzamides with propargylic acetates leading to the formation of NH-free isoquinolones is described. This reaction proceeds through a sequential C-H activation/alkyne insertion/intramolecular annulation/N-O bond cleavage procedure, affording a broad spectrum of products with diverse substituents in moderate-to-excellent yields. Notably, this protocol features the simultaneous formation of two new C-C/C-N bonds and one heterocycle in one pot with the release of water as the sole byproduct.

## 1. Introduction

Isoquinolone is a ubiquitous, structural motif that presents in various natural products, conjugated materials, and pharmaceuticals with a wide range of biological activities [1,2,3,4]. Meanwhile, isoquinolone derivatives, as versatile intermediates, provide wide access to a large variety of chemical molecules and drug structures in various organic transformations [5,6,7]. In this regard, the formation of isoquinolone derivatives has gained significant attention among synthetic and medicinal chemists [8,9,10,11,12,13,14]. Traditional methods to access these valuable derivatives involve the Bischler-Napieralski and Pictet-Spengler reactions [15,16], but they often suffer from the need for pre-activated substrates and harsh reaction conditions, which restrict their regioselectivity. Consequently, the development of efficient and atom/step-economical synthetic methods to construct these structures has attracted considerable attention from synthetic chemists.

**Scheme 1:**
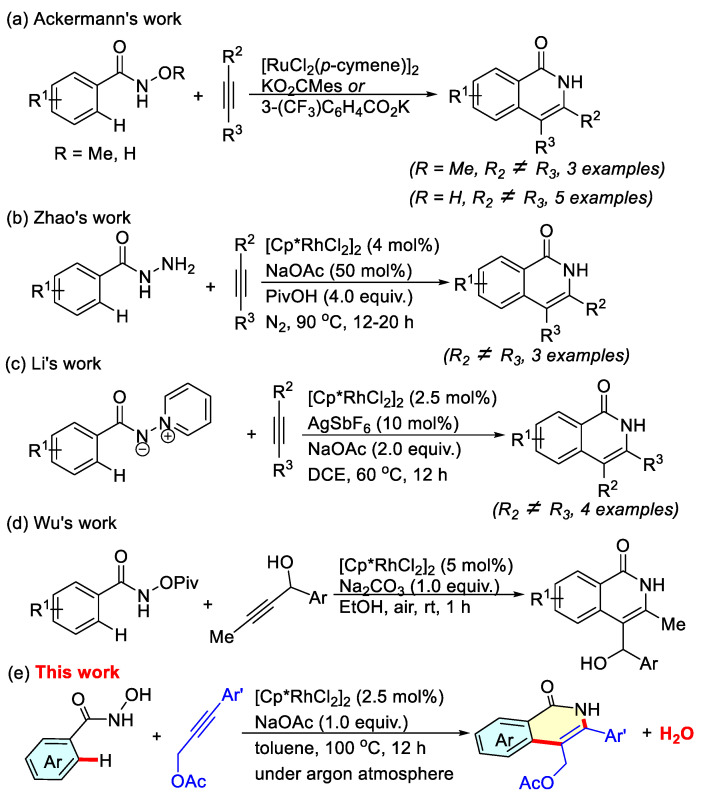
Synthesis of NH-free isoquinolones via C-H activation/annulation strategy.

In the last few decades, transition-metal-catalyzed C-H activation/annulations have been recognized as a powerful and straightforward approach for the synthesis of *N*-heterocycles and, therefore, have attracted increasing attention [17,18,19,20]. Recently, the transition-metal-catalyzed oxidative cyclization of *N*-substituted benzamides with symmetrical alkynes or diazos via C-H bond activation has been an efficient method for constructing *N*-protected isoquinolone derivatives [21,22,23,24,25,26,27,28]. Meanwhile, many other substrates bearing the *N*-directing group have also been used to construct an isoquinolone scaffold through the C-H activation/annulation strategy [29,30,31]. Despite these achievements, directing access to NH-free isoquinolone scaffolds via cascade C-H activation/annulation has attracted our great interest [32]. Ackermann reported the preparations of isoquinolone derivatives by Ru-catalyzed C-H functionalization/annulation of *N*-methoxybenzamides or *N*-hydroxybenzamides with alkynes in water in the presence of MesCO_2_K or 3-(CF_3_)C_6_H_4_CO_2_K as the cocatalytic additive (Scheme 1a) [33,34]. Later, a Rh(III)-catalyzed synthesis of isoquinolones via C-H activation/annulation of benzoylhydrazines and alkynes was further developed (Scheme 1b) [35]. In addition, Li described a highly efficient synthesis pathway by the reaction of iminopyridinium ylides with alkynes for the formation of an isoquinolone skeleton (Scheme 1c) [36]. Very recently, Wu disclosed a robust and convenient rhodium-catalyzed regioselective C-H activation/[4 + 2] annulation using propargyl alcohols as two-carbon synthons to construct 3-methylisoquinolones (Scheme 1d) [37]. Among the limited successful procedures with unsymmetric alkynes serving as the coupling partners, it is desirable to explore a new pathway for furnishing greater structural diversity of NH-free isoquinolone derivatives. We herein describe a novel rhodium(III)-catalyzed cascade C-H activation/annulation of *N*-hydroxybenzamides and propargylic acetates to generate NH-free 3-aryisoquinolones, releasing H_2_O as the sole byproduct (Scheme 1e).

## 2. Results and Discussion

Our study commenced with the reaction between *N*-hydroxybenzamide (**1a**) and 3-phenylprop-2-yn-1-yl acetate (**2a**) in the presence of [Cp * RhCl_2_]_2_ as catalyst (Table 1, entry 5). For experimental condition screening, the catalyst precursor [Cp * RhCl_2_]_2_ was fixed at 2.5 mol%, and, with addition of chloride abstracting reagent NaOAc (1.0 equiv.), the [4 + 2] annulated product **3aa** was isolated in 84% yield after 12 h at 100 °C in toluene (Table 1, entry 5). The reaction was then performed in the presence of other transition metal catalysts, but none of them was as effective as [Cp * RhCl_2_]_2_ (Table 1, entries 1–4). Changing the catalyst loading failed to improve the reaction yield (Table 1, entries 6, 7). Investigation of the additive showed that NaOAc was the best choice (Table 1, entries 8–17). In addition, either decreasing or increasing the loading of additive did not improve this transformation (Table 1, entries 19, 20). Furthermore, several common organic solvents were investigated, and the results indicated that toluene favored this transformation (Table 1, entries 21–26). There was no extra benefit to the product yield at elevated reaction temperature and reaction time (Table 1, entries 27–30). Finally, we chose the reaction conditions of entry 5 as the optimal conditions.

**Scheme 2:**
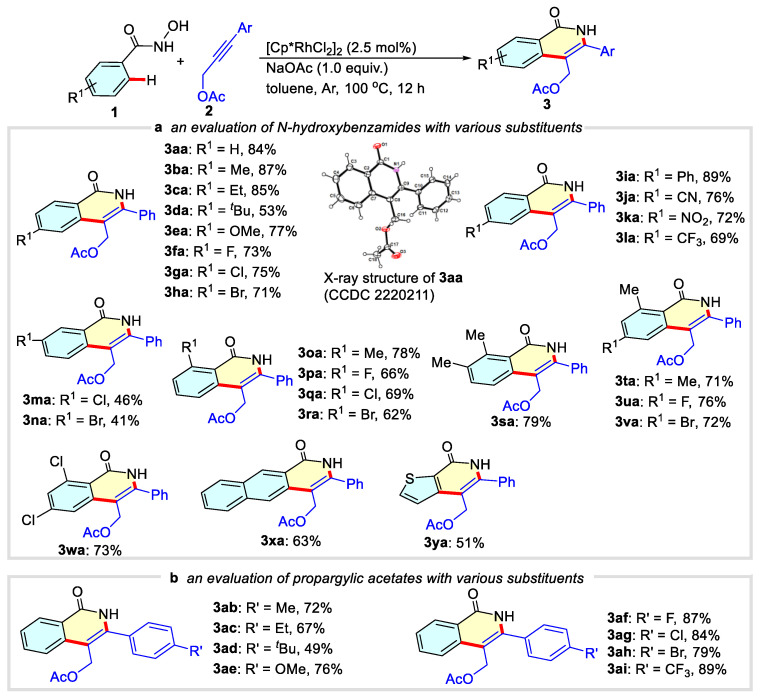
Substrate scope. Reaction conditions: *N*-hydroxybenzamides **1** (0.2 mmol), propargylic acetates **2** (0.2 mmol), [Cp * RhCl_2_]_2_ (2.5 mol%), and NaOAc (0.2 mmol) in toluene at 100 °C for 12 h under argon atmosphere. Isolated yields were reported.

With the establishment of the optimum conditions, we first investigated the scope and generality of *N*-hydroxybenzamide substrates (Scheme 2a). It was found that a variety of substituted *N*-hydroxybenzamides reacted smoothly with 3-phenylprop-2-yn-1-yl acetate **2a** to produce the corresponding isoquinolones in 41–89% yields. In general, *N*-hydroxybenzamides with either electron-donating (e.g., -Me, -Et, -*^t^* Bu, -OMe) or electron-withdrawing (e.g., -F, -Cl, -Br) groups at the *para* position of the benzene ring worked well with **2a** to afford the corresponding products **3ba**–**3ha** in 53–87% yields. Notably, various functional groups, including phenyl and even strong electron-withdrawing substituents -CN, -NO_2_, and -CF_3_, were tolerated well to supply the desired products **3ia**–**3la** in 69–89% yields. Furthermore, *meta*- and *ortho*-substituted substrates were also tolerated regardless of the electronic property on the benzene ring, providing the desired isoquinolones **3ma**–**3ra** in 41–78% yields. In addition, disubstituted *N*-hydroxybenzamides were also reactive to produce the corresponding products in 71–79% yields (products **3sa**–**3wa**). Moreover, this transformation was further extended to *N*-hydroxy-2-naphthamide and *N*-hydroxythiophene-2-carboxamide substrates, giving the corresponding products **3xa** and **3ya** in 63% and 51% yields, respectively. Subsequently, we probed the scope of this transformation, employing propargylic acetates **2** bearing diverse substituents at the *para* position of the benzene ring and leading to the corresponding products **3ab**–**3ai** in 49–89% yields (Scheme 2b). Encouragingly, the halo groups on either benzamide, as well as on the propargylic acetates moiety, were well tolerated to produce the target products, which may have potential applications in organic synthesis by further functionalization through Pd-catalyzed coupling reactions.

**Scheme 3:**
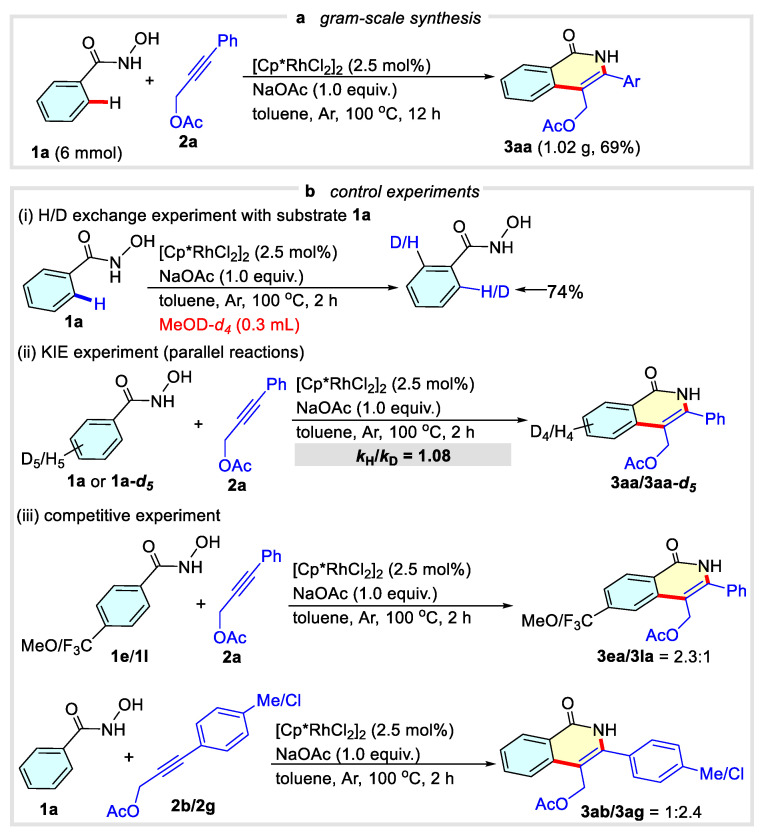
Gram-scale synthesis and control experiments.

To further prove the robustness and the general utility of this protocol, we carried out the reaction of *N*-hydroxybenzamide **1a** and 3-phenylprop-2-yn-1-yl acetate **2a** in gram-scale synthesis under the standard condition. This transformation was easily scaled up to 6 mmol (scaled up to 30 times), producing the desired product **3aa** in 69% yield (Scheme 3a). Subsequently, insights into this cascade C-H activation/annulation were gained by performing control experiments to clarify the reaction mechanism. Treating *N*-hydroxybenzamide **1a** with methanol-*d_4_* under standard conditions for 2 h, a 74% deuterium was detected on the *ortho* C-H bond (Scheme 3b(i)), which indicated that the C-H activation process might be the reversible step. Next, a kinetic isotope effect (KIE) value of 1.08 was measured from two parallel reactions of *N*-hydroxybenzamide **1a** or **1a**-*d_5_* with propargylic acetate **2a** for 2 h under the standard conditions (Scheme 3b(ii)). The intermolecular competitive experiment of *para*-methoxyl- and *para*-trifluoromethyl-substituted *N*-hydroxybenzamide showed that the electron-donating group facilitated the reaction step, implying that C(*sp^2^*)-H bond cleavage might be the limiting step (Scheme 3b(iii)). As for the distinct propargylic acetates **2b** and **2g**, the electron-deficient propargylic acetate **2g** delivered the product in a higher yield.

**Scheme 4:**
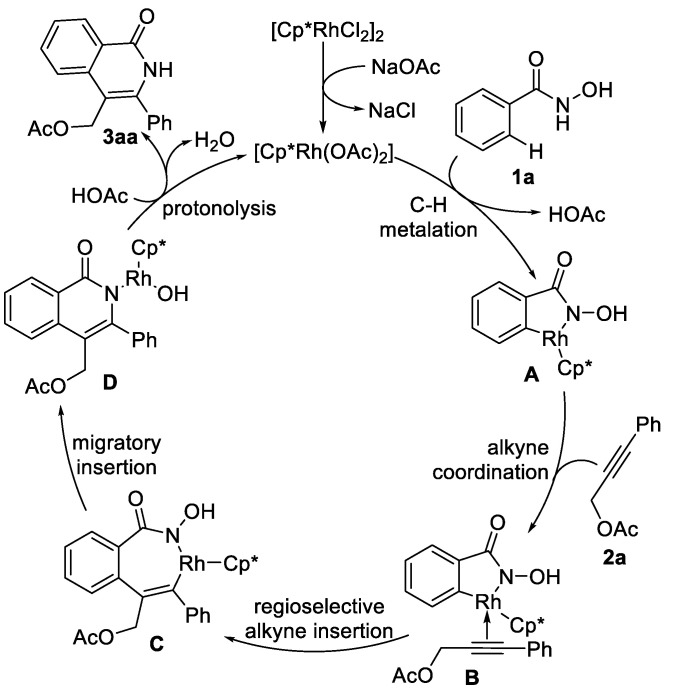
Proposed mechanism for the formation of NH-free isoquinolones.

On the basis of the above control experiments and relevant reports [33,36,38], a plausible mechanism for the Rh-catalyzed cascade C-H activation/annulation for the formation of NH-free isoquinolones is proposed in Scheme 4. Initially, the active catalyst was generated via ligand exchange, which mediated a facile C-H metalation process to give the five-membered rhodacycle **A**. Subsequent coordination of the alkyne to the rhodium center followed by the 1,2-insertion of the alkyne afforded the seven-membered rhodacycle **C**. Then, the metal migration and C-N bond formation of intermediate **C** afforded the intermediate **D**, which underwent the protonation to generate the desired product **3aa** along with the release of a molecule of water.

## 3. Materials and Methods

The detailed procedures for the synthesis and characterization of the products are given in Appendix A.

## 4. Conclusions

In conclusion, we developed an efficient and practical method to construct NH-free isoquinolones via Rh(III)-catalyzed C-H activation/annulation of *N*-hydroxybenzamides and propargylic acetates. A variety of *N*-hydroxybenzamides with a diverse array of substituents, irrespective of their electronic and steric nature, were tolerated well under the optimal conditions. Generation of H_2_O as the only byproduct makes this 100% carbon-efficient process attractive for the synthesis of isoquinolone derivatives. Moreover, the synthetic utility and practicability of the developed methodology in gram-scale synthesis were validated.

## Figures and Tables

**Table 1:** Optimization of the reaction conditions *^a^*.

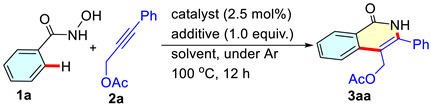				
Entry	Catalyst	Additive	Solvent	Yield (%) *^b^*
1	Pd(PPh_3_)_4_	NaOAc	toluene	0
2	Pd(OAc)_2_	NaOAc	toluene	trace
3	[RuCl_2_(*p*-cymene)]_2_	NaOAc	toluene	37
4	[Cp * IrCl_2_]_2_	NaOAc	toluene	65
5	[Cp * RhCl_2_]_2_	NaOAc	toluene	84
6 *^c^*	[Cp * RhCl_2_]_2_	NaOAc	toluene	46
7 *^d^*	[Cp * RhCl_2_]_2_	NaOAc	toluene	71
8	[Cp * RhCl_2_]_2_	CsOAc	toluene	52
9	[Cp * RhCl_2_]_2_	AgOAc	toluene	41
10	[Cp * RhCl_2_]_2_	NaHCO_3_	toluene	67
11	[Cp * RhCl_2_]_2_	Na_2_CO_3_	toluene	63
12	[Cp * RhCl_2_]_2_	KHCO_3_	toluene	61
13	[Cp * RhCl_2_]_2_	K_2_CO_3_	toluene	30
14	[Cp * RhCl_2_]_2_	Et_3_N	toluene	64
15	[Cp * RhCl_2_]_2_	AgSbF_6_	toluene	29
16	[Cp * RhCl_2_]_2_	AgNTf_2_	toluene	17
17	[Cp * RhCl_2_]_2_	Cs_2_CO_3_	MeOH	48
18	[Cp * RhCl_2_]_2_	-	toluene	0
19 *^e^*	[Cp * RhCl_2_]_2_	NaOAc	toluene	62
20 *^f^*	[Cp * RhCl_2_]_2_	NaOAc	toluene	80
21	[Cp * RhCl_2_]_2_	NaOAc	DCE	62
22	[Cp * RhCl_2_]_2_	NaOAc	MeCN	43
23	[Cp * RhCl_2_]_2_	NaOAc	DMF	54
24	[Cp * RhCl_2_]_2_	NaOAc	THF	67
25	[Cp * RhCl_2_]_2_	NaOAc	1,4-dioxane	59
26	[Cp * RhCl_2_]_2_	NaOAc	*o*-xylene	60
27 *^g^*	[Cp * RhCl_2_]_2_	NaOAc	toluene	61
28 *^h^*	[Cp * RhCl_2_]_2_	NaOAc	toluene	84
29 *^i^*	[Cp * RhCl_2_]_2_	NaOAc	toluene	50
30 *^j^*	[Cp * RhCl_2_]_2_	NaOAc	toluene	83

*^a^* Reaction conditions: *N*-hydroxybenzamide **1a** (0.2 mmol), 3-phenylprop-2-yn-1-yl acetate **2a** (0.2 mmol), catalyst (2.5 mol%), and additive (0.2 mmol) in solvent at 100 °C for 12 h under argon atmosphere. *^b^* Isolated yields. *^c^* The amount of catalyst was 1 mol%. *^d^* The amount of catalyst was 5 mol%. *^e^* The amount of additive was 0.5 equivalent. *^f^* The amount of additive was 1.5 equivalent. *^g^* At 80 °C. *^h^* At reflux. *^i^* For 5 h. *^j^* For 15 h.

## Data Availability

The data presented in this study are available in this article.

## Supplementary Materials

The following supporting information can be downloaded at: https://www.mdpi.com/article/10.3390/molecules27238553/s1, Characterization data for product **3**, including ^1^H- and ^13^C-NMR spectroscopies, are available online. CCDC 2220211 contains the supplementary crystallographic data for this paper. These data can be obtained free of charge via www.ccdc.cam.ac.uk/data_request/cif or by emailing data_request@ccdc.cam.ac.uk or by contacting the Cambridge Crystallographic Data Centre, 12 Union Road, Cambridge, CB2 1EZ, UK; fax: +44-1223-336033.

## Appendix A. Experimental Section

Unless otherwise noted, all reagents were purchased from commercial suppliers and used without purification. All cascade reactions were performed in a resealable screw-capped Schlenk flask (approx. 15 mL volume) in the presence of a Teflon-coated magnetic stirrer bar (4 mm × 10 mm). Reactions were monitored by using thin-layer chromatography (TLC) on commercial silica gel plates (GF 254). Visualization of the developed plates was performed under UV lights (GF 254 nm). Flash column chromatography was performed on silica gel (200–300 mesh). ^1^H NMR spectra were recorded on a 500 MHz spectrometer, and ^13^C NMR spectra were recorded on a 125 MHz spectrometer (Supplementary Materials: ^1^H NMR and ^13^C NMR). Chemical shifts were expressed in parts per million (*δ*), and the signals were reported as s (singlet), d (doublet), dd (doublet of doublet), t (triplet), q (quartet), and m (multiplet), and coupling constants (*J*) were given in Hz. Chemical shifts as internal standards were referenced to CDCl_3_ (*δ* = 7.26 for ^1^H and *δ* = 77.16 for ^13^C NMR) as internal standard. HRMS analysis with a quadrupole time-of-flight mass spectrometer yielded ion mass/charge (*m/z*) ratios in atomic mass units. The melting points were measured using SGWX-4 melting point apparatus and were not corrected. The X-ray source used for the single-crystal X-ray diffraction analysis of compound **3aa** was Mo Kα (λ = 0.71073 Å), and the thermal ellipsoid was drawn at the 30% probability level (Supplementary Materials: X-ray crystal data).

**General procedure for the synthesis of isoquinolones 3** (product **3aa** as an example). *N*-Hydroxybenzamides **1a** (27.4 mg, 0.2 mmol), [Cp * RhCl_2_]_2_ (3.1 mg, 2.5 mol%), and NaOAc (16.4 mg, 0.2 mmol) were loaded into a Schlenk tube equipped with a Teflon-coated magnetic stir bar. The tube was evacuated and flushed with argon for three cycles. Propargylic acetates **2a** (34.8 mg, 0.2 mmol) and toluene (2 mL) were then added, and the tube was placed into a preheated oil bath (100 °C) and stirred for 12 h. After the completion of the reaction, the reaction tube was allowed to cool to room temperature, extracted with CH_2_Cl_2_ (3 × 10 mL), and washed with brine. The organic layers were combined, dried over Na_2_SO_4_, filtered, and then evaporated under vacuum. The residue was purified using flash column chromatography with a silica gel (200–300 mesh) and using ethyl acetate and petroleum ether as the elution solvent to give desired product **3**.

*(1-Oxo-3-phenyl-1,2-dihydroisoquinolin-4-yl)methyl acetate (**3aa**)*. This compound was purified by column chromatography (ethyl acetate/petroleum ether = 1:6) to provide a white solid in 84% yield (49 mg); mp 163–164 °C; ^1^H NMR (500 MHz, DMSO-*d_6_*) *δ* 11.58 (s, 1H), 8.29 (d, *J* = 8.0 Hz, 1H), 7.82–7.75 (m, 2H), 7.58–7.47 (m, 6H), 4.97 (s, 2H), 2.03 (s, 3H); ^13^C NMR (100 MHz, DMSO-*d_6_*) *δ* 170.9, 162.2, 143.2, 137.5, 133.9, 133.4, 129.9, 129.8, 128.9, 127.5, 127.0, 125.8, 124.1, 106.8, 60.7, 21.2; HRMS (ESI-TOF) *m/z*: [M + H]^+^ Calcd for C_18_H_16_NO_3_ 294.1125; Found 294.1127.

*(6-Methyl-1-oxo-3-phenyl-1,2-dihydroisoquinolin-4-yl)methyl acetate (**3ba**)*. This compound was purified by column chromatography (ethyl acetate/petroleum ether = 1:6) to provide a white solid in 87% yield (53 mg); mp 166–167 °C; ^1^H NMR (500 MHz, CDCl_3_) *δ* 9.52 (s, 1H), 8.29 (d, *J* = 8.5 Hz, 1H), 7.52–7.47 (m, 6H), 7.36 (d, *J* = 8.0 Hz, 1H), 5.13 (s, 2H), 2.53 (s, 3H), 2.12 (s, 3H); ^13^C NMR (125 MHz, CDCl_3_) *δ* 171.4, 163.1, 144.4, 142.4, 142.3, 138.0, 134.3, 130.3, 129.3, 128.9, 128.3, 123.6, 108.0, 61.2, 22.7, 21.5; HRMS (ESI-TOF) *m/z*: [M + H]^+^ Calcd for C_19_H_18_NO_3_ 308.1281; Found 308.1283.

*(6-Ethyl-1-oxo-3-phenyl-1,2-dihydroisoquinolin-4-yl)methyl acetate (**3ca**)*. This compound was purified by column chromatography (ethyl acetate/petroleum ether = 1:6) to provide a white solid in 85% yield (54 mg); mp 172–173 °C; ^1^H NMR (500 MHz, CDCl_3_) *δ* 9.76 (s, 1H), 8.30 (d, *J* = 8.0 Hz, 1H), 7.50–7.48(m, 6H), 7.39 (d, *J* = 8.0 Hz, 1H), 5.15 (s, 2H), 2.82 (q, *J* = 7.5, 2H), 2.12 (s, 3H), 1.31 (t, *J* = 7.5 Hz, 3H); ^13^C NMR (125 MHz, CDCl_3_) *δ* 171.4, 163.3, 150.5, 142.4, 138.1, 134.3, 130.3, 129.4, 129.3, 128.4, 127.8, 123.8, 122.4, 108.2, 61.2, 29.9, 21.5, 15.8; HRMS (ESI-TOF) *m/z*: [M + H]^+^ Calcd for C_20_H_20_NO_3_ 322.1438; Found 322.1430.

*(6-(tert-Butyl)-1-oxo-3-phenyl-1,2-dihydroisoquinolin-4-yl)methyl acetate (**3da**)*. This compound was purified by column chromatography (ethyl acetate/petroleum ether = 1:6) to provide a white solid in 53% yield (37 mg); mp 174–175 °C; ^1^H NMR (500 MHz, CDCl_3_) *δ* 8.68 (s, 1H), 8.38 (d, *J* = 8.5 Hz, 1H), 7.74–7.68 (m, 1H), 7.64–7.57 (m, 1H), 7.54–7.47 (m, 5H), 5.21 (s, 2H), 2.10 (s, 3H), 1.41 (s, 9H); ^13^C NMR (125 MHz, CDCl_3_) *δ* 171.3, 162.7, 157.3, 141.9, 137.6, 134.5, 130.3, 129.5, 129.2, 128.1, 125.4, 123.6, 120.1, 108.6, 61.0, 35.9, 31.5, 21.3; HRMS (ESI-TOF) *m/z*: [M + H]^+^ Calcd for C_22_H_24_NO_3_ 350.1751; Found 350.1759.

*(6-Methoxy-1-oxo-3-phenyl-1,2-dihydroisoquinolin-4-yl)methyl acetate (**3ea**)*. This compound was purified by column chromatography (ethyl acetate/petroleum ether = 1:6) to provide a white solid in 77% yield (49 mg); mp 172–173 °C; ^1^H NMR (500 MHz, CDCl_3_) *δ* 9.62 (s, 1H), 8.32 (d, *J* = 10.0 Hz, 1H), 7.51 (s, 5H), 7.14–7.05 (m, 2H), 5.14 (s, 2H), 3.92 (s, 3H), 2.11 (s, 3H); ^13^C NMR (125 MHz, CDCl_3_) *δ* 171.4, 164.1, 162.4, 142.8, 140.0, 134.4, 130.4, 130.4, 129.5, 129.2, 119.6, 116.1, 108.1, 105.9, 61.0, 55.9, 21.4; HRMS (ESI-TOF) *m/z*: [M + H]^+^ Calcd for C_19_H_18_NO_4_ 324.1230; Found 324.1228.

*(6-Fluoro-1-oxo-3-phenyl-1,2-dihydroisoquinolin-4-yl)methyl acetate (**3fa**)*. This compound was purified by column chromatography (ethyl acetate/petroleum ether = 1:6) to provide a white solid in 73% yield (45 mg); mp 161–162 °C; ^1^H NMR (500 MHz, CDCl_3_) *δ* 9.00 (s, 1H), 8.42 (m, 1H), 7.53–7.47 (m, 6H), 7.35 (dd, *J* = 10.5 Hz, 2.0 Hz, 1H), 5.07 (s, 2H), 2.10 (s, 3H); ^13^C NMR (125 MHz, CDCl_3_) *δ* 171.3, 166.4 (d, *J_C-F_* = 250.1 Hz), 162.4, 143.6, 140.6 (d, *J_C-F_* = 9.9 Hz), 133.7, 131.5 (d, *J_C-F_* = 10.1 Hz), 130.4, 129.2 (d, *J_C-F_* = 26.2 Hz), 115.9(d, *J_C-F_* = 23.1 Hz), 115.6, 109.4, 109.2, 60.8, 21.2; HRMS (ESI-TOF) *m/z*: [M + H]^+^ Calcd for C_18_H_15_FNO_3_ 312.1030; Found 312.1022.

*(6-Chloro-1-oxo-3-phenyl-1,2-dihydroisoquinolin-4-yl)methyl acetate (**3ga**).* This compound was purified by column chromatography (ethyl acetate/petroleum ether = 1:6) to provide a white solid in 75% yield (49 mg); mp 174–175 °C; ^1^H NMR (500 MHz, CDCl_3_) *δ* 9.37 (s, 1H), 8.38 (d, *J* = 2.0 Hz, 1H), 7.70–7.64 (m, 2H), 7.54–7.48 (m, 5H), 5.13 (s, 2H), 2.10 (s, 3H); ^13^C NMR (125 MHz, CDCl_3_) *δ* 171.3, 161.9, 142.4, 136.3, 134.1, 133.9, 133.5, 130.6, 129.5, 129.2, 127.8, 127.1, 125.7, 108.1, 60.8, 21.4; HRMS (ESI-TOF) *m/z*: [M + H]^+^ Calcd for C_18_H_15_ClNO_3_ 328.0735; Found 328.0727.

*(6-Bromo-1-oxo-3-phenyl-1,2-dihydroisoquinolin-4-yl)methyl acetate (**3ha**)*. This compound was purified by column chromatography (ethyl acetate/petroleum ether = 1:6) to provide a white solid in 71% yield (52 mg); mp 164–165 °C; ^1^H NMR (500 MHz, CDCl_3_) *δ* 9.20 (s, 1H), 8.26 (d, *J* = 9.0 Hz, 1H), 7.86 (d, *J* = 2.0 Hz, 1H), 7.64 (dd, *J* = 8.5, 2.0 Hz, 1H), 7.56–7.46 (m, 5H), 5.08 (s, 2H), 2.12 (s, 3H); ^13^C NMR (125 MHz, CDCl_3_) *δ* 171.3, 162.5, 143.4, 139.5, 133.9, 130.7, 130.7, 130.0, 129.5, 129.2, 129.2, 126.8, 124.6, 107.5, 60.8, 21.4; HRMS (ESI-TOF) *m/z*: [M + H]^+^ Calcd for C_18_H_15_BrNO_3_ 372.0230; Found 372.0235.

*(1-Oxo-3,6-diphenyl-1,2-dihydroisoquinolin-4-yl)methyl acetate (**3ia**)*. This compound was purified by column chromatography (ethyl acetate/petroleum ether = 1:6) to provide a white solid in 89% yield (65 mg); mp 181–182 °C; ^1^H NMR (500 MHz, CDCl_3_) *δ* 8.68 (s, 1H), 8.52 (d, *J* = 8 Hz, 1H), 7.92 (s, 1H), 7.80 (dd, *J* = 8.5, 1Hz, 1H), 7.68 (d, *J* = 5.0 Hz, 2H), 7.49–7.55 (m, 7H), 7.44 (m, 1H), 5.21 (s, 2H), 2.12 (s, 3H); ^13^C NMR (125 MHz, CDCl_3_) *δ* 171.4, 163.1, 146.4, 142.8, 140.6, 138.3, 134.3, 130.4, 129.5, 129.4, 129.4, 129.2, 128.8, 127.9, 126.5, 124.7, 122.1, 108.5, 61.1, 21.5; HRMS (ESI-TOF) *m/z*: [M + H]^+^ Calcd for C_24_H_20_NO_3_ 370.1438; Found 370.1439.

*(6-Cyano-1-oxo-3-phenyl-1,2-dihydroisoquinolin-4-yl)methyl acetate (**3ja**)*. This compound was purified by column chromatography (ethyl acetate/petroleum ether = 1:6) to provide a white solid in 76% yield (48 mg); mp 205–206 °C; ^1^H NMR (500 MHz, CDCl_3_) *δ* 9.42 (s, 1H), 8.50 (d, *J* = 8.0 Hz, 1H), 8.05 (s, 1H), 7.74 (dd, *J* = 8.0, 1Hz, 1H), 7.59–7.52 (m, 6H), 5.12 (s, 2H), 2.13 (s, 3H); ^13^C NMR (125 MHz, CDCl_3_) *δ* 171.1, 161.8, 144.1, 138.2, 133.5, 131.0, 129.5, 129.4, 129.2, 129.1, 128.9, 128.4, 118.6, 117.3, 107.6, 60.6, 21.4; HRMS (ESI-TOF) *m/z*: [M + H]^+^ Calcd for C_19_H_15_N_2_O_3_ 319.1077; Found 319.1079.

*(6-Nitro-1-oxo-3-phenyl-1,2-dihydroisoquinolin-4-yl)methyl acetate (**3ka**)*. This compound was purified by column chromatography (ethyl acetate/petroleum ether = 1:6) to provide a yellow solid in 72% yield(48 mg); mp 191–192 °C; ^1^H NMR (500 MHz, CDCl_3_) *δ* 10.16 (s, 1H), 8.61 (s, 1H), 8.54 (d, *J* = 9.0 Hz, 1H), 8.39–8.21 (m, 1H), 7.60–7.50 (m, 5H), 5.19 (s, 2H), 2.12 (s, 3H); ^13^C NMR (125 MHz, CDCl_3_) *δ* 171.1, 161.9, 151.3, 144.4, 138.9, 133.4, 131.0, 130.4, 129.6, 129.5, 129.2, 120.9, 119.8, 108.5, 60.7, 21.3; HRMS (ESI-TOF) *m/z*: [M + H]^+^ Calcd for C_18_H_15_N_2_O_5_ 339.0975; Found 339.0971.

*(1-Oxo-3-phenyl-6-(trifluoromethyl)-1,2-dihydroisoquinolin-4-yl)methyl acetate (**3la**)*. This compound was purified by column chromatography (ethyl acetate/petroleum ether = 1:6) to provide a white solid in 69% yield (50 mg); mp 193–194 °C; ^1^H NMR (500 MHz, CDCl_3_) *δ* 9.40 (s, 1H), 8.53 (d, *J* = 8.0 Hz, 1H), 7.99 (s, 1H), 7.74 (d, *J* = 8.5 Hz, 1H), 7.57–7.53 (m, 3H), 7.52–7.48 (m, 2H), 5.16 (s, 2H), 2.11 (s, 3H); ^13^C NMR (125 MHz, CDCl_3_) *δ* 171.0, 162.5, 143.7, 137.9, 135.1 (q, *J_C-F_* = 32.5 Hz), 133.4, 130.4, 129.3, 129.1, 127.7, 125.18 (q, *J_C-F_* = 271.3 Hz), 123.0, 122.8, 121.1, 108.1, 60.6, 21.1; HRMS (ESI-TOF) *m/z*: [M + H]^+^ Calcd for C_19_H_15_F_3_NO_3_ 362.0999; Found 362.1007.

*(7-Chloro-1-oxo-3-phenyl-1,2-dihydroisoquinolin-4-yl)methyl acetate (**3ma**)*. This compound was purified by column chromatography (ethyl acetate/petroleum ether = 1:6) to provide a white solid in 46% yield (30 mg); mp 177–178 °C; ^1^H NMR (500 MHz, CDCl_3_) *δ* 9.11 (s, 1H), 8.39 (d, *J* = 2.0 Hz, 1H), 7.70 (dd, *J* = 10.0, 5.0 Hz, 1H), 7.66 (d, *J* = 10.0 Hz, 1H), 7.57–7.51 (m, 3H), 7.49 (dd, *J* = 5.0, 1.5 Hz, 2H), 5.12 (s, 2H), 2.11 (s, 3H); ^13^C NMR (125 MHz, CDCl_3_) *δ* 171.3, 161.9, 142.3, 136.3, 134.1, 133.9, 133.6, 130.6, 129.5, 129.2, 127.8, 127.1, 125.7, 108.1, 60.8, 21.4; HRMS (ESI-TOF) *m/z*: [M + H]^+^ Calcd for C_18_H_15_ClNO_3_ 328.0735; Found 328.0741.

*(7-Bromo-1-oxo-3-phenyl-1,2-dihydroisoquinolin-4-yl)methyl acetate (**3na**)*. This compound was purified by column chromatography (ethyl acetate/petroleum ether = 1:6) to provide a white solid in 41% yield (30 mg); mp 172–173 °C; ^1^H NMR (500 MHz, CDCl_3_) *δ* 9.77 (s, 1H), 8.50 (s, 1H), 7.82 (dd, *J* = 5.0, 1.5 Hz, 1H), 7.58 (d, *J* = 10.0 Hz, 1H), 7.56–7.47 (m, 5H), 5.12 (s, 2H), 2.10 (s, 3H); ^13^C NMR (125 MHz, CDCl_3_) *δ* 171.0, 161.6, 142.4, 136.6, 136.5, 133.8, 130.8, 130.5, 129.3, 129.0, 127.2, 125.6, 121.2, 107.9, 60.6, 21.2; HRMS (ESI-TOF) *m/z*: [M + H]^+^ Calcd for C_18_H_15_BrNO_3_ 372.0230; Found 372.0231.

*(8-Methyl-1-oxo-3-phenyl-1,2-dihydroisoquinolin-4-yl)methyl acetate (**3oa**)*. This compound was purified by column chromatography (ethyl acetate/petroleum ether = 1:6) to provide a white solid in 78% yield (48 mg); mp 182–183 °C; ^1^H NMR (500 MHz, CDCl_3_) *δ* 10.77 (s, 1H), 7.56–7.48 (m, 7H), 7.24 (d, *J* = 7.0 Hz, 1H), 5.13 (s, 2H), 2.76 (s, 3H), 2.10 (s, 3H); ^13^C NMR (125 MHz, CDCl_3_) *δ* 171.5, 164.8, 142.8, 139.7, 134.1, 132.7, 130.2, 130.0, 129.6, 129.1, 124.2, 121.7, 108.0, 61.7, 24.3, 21.5; HRMS (ESI-TOF) *m/z*: [M + H]^+^ Calcd for C_19_H_18_NO_3_ 308.1281; Found 308.1286.

*(8-Fluoro-1-oxo-3-phenyl-1,2-dihydroisoquinolin-4-yl)methyl acetate (**3pa**)*. This compound was purified by column chromatography (ethyl acetate/petroleum ether = 1:6) to provide a brownish yellow solid in 66% yield (41 mg); mp 164–165 °C; ^1^H NMR (500 MHz, CDCl_3_) *δ* 9.45 (s, 1H), 7.70–7.66 (m, 1H), 7.53–7.49 (m, 5H), 7.47 (d, *J* = 5.0 Hz, 1H), 7.17 (dd, *J* = 15.0 Hz, 5.0 Hz, 1H), 5.11 (s, 2H), 2.11 (s, 3H); ^13^C NMR (125 MHz, CDCl_3_) *δ* 171.3, 163.3 (d, *J_C-F_* = 262.5 Hz), 160.6, 143.6, 140.8, 134.6 (d, *J_C-F_* = 10.0 Hz), 133.7, 130.6, 129.5, 129.3 (d, *J_C-F_* = 7.5 Hz), 119.7 (d, *J_C-F_* = 5.0 Hz), 115.1 (d, *J_C-F_* = 5.0 Hz), 114.2 (d, *J_C-F_* = 21.2 Hz), 107.5, 61.3, 21.4; HRMS (ESI-TOF) *m/z*: [M + H]^+^ Calcd for C_18_H_15_FNO_3_ 312.1030; Found 312.1031.

*(8-Chloro-1-oxo-3-phenyl-1,2-dihydroisoquinolin-4-yl)methyl acetate (**3qa**)*. This compound was purified by column chromatography (ethyl acetate/petroleum ether = 1:6) to provide a brownish yellow solid in 69% yield (45 mg); mp 171–172 °C; ^1^H NMR (500 MHz, CDCl_3_) *δ* 10.28 (s, 1H), 7.60–7.49 (m, 8H), 5.11 (s, 2H), 2.11 (s, 3H); ^13^C NMR (125 MHz, CDCl_3_) *δ* 171.4, 161.8, 143.8, 141.1, 136.4, 133.6, 133.2, 130.5, 130.4, 129.4, 129.4, 122.8, 122.4, 107.6, 61.4, 21.4; HRMS (ESI-TOF) *m/z*: [M + H]^+^ Calcd for C_18_H_15_ClNO_3_ 328.0735; Found 328.0744.

*(8-Bromo-1-oxo-3-phenyl-1,2-dihydroisoquinolin-4-yl)methyl acetate (**3ra**)*. This compound was purified by column chromatography (ethyl acetate/petroleum ether = 1:6) to provide a brownish yellow solid in 62% yield (46 mg); mp 168–169 °C; ^1^H NMR (500 MHz, CDCl_3_) *δ* 10.21 (s, 1H), 7.76 (d, *J* = 7.5, 1H), 7.64 (dd, *J* = 8.5, 1.0 Hz, 1H), 7.55–7.50 (m, 5H), 7.47 (t, *J* = 8.0 Hz, 1H), 5.10 (s, 2H), 2.10 (s, 3H); ^13^C NMR (125 MHz, CDCl_3_) *δ* 171.4, 161.8, 143.6, 141.1, 134.3 133.7, 133.3, 130.4, 129.4, 123.8, 123.5, 123.3, 107.7, 61.4, 21.4; HRMS (ESI-TOF) *m/z*: [M + H]^+^ Calcd for C_18_H_15_BrNO_3_ 372.0230; Found 372.0237.

*(7,8-Dimethyl-1-oxo-3-phenyl-1,2-dihydroisoquinolin-4-yl)methyl acetate (**3sa**)*. This compound was purified by column chromatography (ethyl acetate/petroleum ether = 1:6) to provide a white solid in 79% yield (51 mg); mp 198–199 °C; ^1^H NMR (500 MHz, CDCl_3_) *δ* 10.14 (s, 1H), 7.56–7.46 (m, 6H), 7.42 (d, *J* = 8.3 Hz, 1H), 5.12 (s, 2H), 2.78 (s, 3H), 2.42 (s, 3H), 2.10 (s, 3H); ^13^C NMR (125 MHz, CDCl_3_) *δ* 171.5, 164.7, 141.6, 140.8, 137.8, 136.6, 135.3, 134.2, 130.0, 129.5, 129.2, 124.2, 120.9, 107.9, 61.7, 21.5, 21.4, 18.3; HRMS (ESI-TOF) *m/z*: [M + H]^+^ Calcd for C_20_H_20_NO_3_ 322.1438; Found 322.1444.

*(6,8-Dimethyl-1-oxo-3-phenyl-1,2-dihydroisoquinolin-4-yl)methyl acetate (**3ta**)*. This compound was purified by column chromatography (ethyl acetate/petroleum ether = 1:6) to provide a light yellow solid in 71% yield (45 mg); mp 214–215 °C; ^1^H NMR (500 MHz, CDCl_3_) *δ* 10.75 (s, 1H), 7.56–7.45 (m, 5H), 7.28 (s, 1H), 7.07 (s, 1H), 5.10 (s, 2H), 2.71 (s, 3H), 2.44 (s, 3H), 2.11 (s, 3H); ^13^C NMR (125 MHz, CDCl_3_) *δ* 171.6, 164.7, 143.1, 142.9, 142.6, 139.8, 134.3, 131.8, 129.9, 129.6, 129.1, 121.9, 121.6, 107.7, 61.8, 24.1, 22.4, 21.5; HRMS (ESI-TOF) *m/z*: [M + H]^+^ Calcd for C_20_H_20_NO_3_ 322.1438; Found 322.1437.

*(6-Fluoro-8-methyl-1-oxo-3-phenyl-1,2-dihydroisoquinolin-4-yl)methyl acetate (**3ua**)*. This compound was purified by column chromatography (ethyl acetate/petroleum ether = 1:6) to provide a white solid in 76% yield (49 mg); mp 182–183 °C; ^1^H NMR (500 MHz, DMSO-*d_6_*) *δ* 11.43 (s, 1H), 7.53–7.48 (m, 3H), 7.48–7.39 (m, 2H), 7.30 (d, *J* = 10.0 Hz, 1H), 7.19 (d, *J* = 10.0 Hz, 1H), 4.88 (s, 2H), 2.84 (s, 3H), 2.02 (s, 3H); ^13^C NMR (100 MHz, DMSO-*d_6_*) *δ* 170.4, 164.1 (d, *J* = 247.2 Hz), 162.5, 145.8 (d, *J* = 1.0 Hz), 144.2, 141.9 (d, *J* = 10.5 Hz), 133.1, 129.6, 129.2, 128.4, 120.6, 117.1 (d, *J* = 27.5 Hz), 107.0, 106.8, 106.0 (d, *J* = 5.0 Hz), 60.6, 23.7, 20.8; HRMS (ESI-TOF) *m/z*: [M + H]^+^ Calcd for C_19_H_17_FNO_3_ 326.1187; Found 326.1179.

*(6-Bromo-8-methyl-1-oxo-3-phenyl-1,2-dihydroisoquinolin-4-yl)methyl acetate (**3va**)*. This compound was purified by column chromatography (ethyl acetate/petroleum ether = 1:6) to provide a white solid in 72% yield (55 mg); mp 180–181 °C; ^1^H NMR (500 MHz, DMSO-*d_6_*) *δ* 11.49 (s, 1H), 7.69 (s, 1H), 7.50 (s, 4H), 7.42 (d, *J* = 5.0 Hz, 2H), 4.88 (s, 2H), 2.80 (s, 3H), 2.01 (s, 3H); ^13^C NMR (100 MHz, DMSO-*d_6_*) *δ* 170.8, 162.7, 144.7, 144.2, 140.9, 133.5, 132.1, 130.1, 129.7, 128.9, 126.8, 124.4, 123.0, 105.9, 60.8, 23.7, 21.2; HRMS (ESI-TOF) *m/z*: [M + H]^+^ Calcd for C_19_H_17_BrNO_3_ 386.0386; Found 386.0377.

*(6,8-Dichloro-1-oxo-3-phenyl-1,2-dihydroisoquinolin-4-yl)methyl acetate (**3wa**)*. This compound was purified by column chromatography (ethyl acetate/petroleum ether = 1:6) to provide a white solid in 73% yield (53 mg); mp 217–218 °C; ^1^H NMR (500 MHz, CDCl_3_) *δ* 9.74 (s, 1H), 7.57–7.25 (m, 7H), 5.05 (s, 2H), 2.12 (s, 3H); ^13^C NMR (125 MHz, CDCl_3_) *δ* 171.2, 161.0, 144.8, 141.9, 139.3, 137.6, 133.3, 130.8, 130.3, 129.5, 129.2, 122.6, 120.9, 107.0, 61.2, 21.4; HRMS (ESI-TOF) *m/z*: [M + H]^+^ Calcd for C_18_H_14_Cl_2_NO_3_ 362.0345; Found 362.0339.

*(1-Oxo-3-phenyl-1,2-dihydrobenzo[g]isoquinolin-4-yl)methyl acetate (**3xa**)*. This compound was purified by column chromatography (ethyl acetate/petroleum ether = 1:6) to provide a yellow solid in 63% yield (43 mg); mp 185–186 °C; ^1^H NMR (500 MHz, CDCl_3_) *δ* 9.24 (s, 1H), 9.03 (s, 1H), 8.15 (s, 1H), 8.07 (d, *J* = 8.0 Hz, 1H), 7.99 (d, *J* = 8.0 Hz, 1H), 7.66–7.60 (m, 1H), 7.59–7.50 (m, 6H), 5.26 (s, 2H), 2.14 (s, 3H); ^13^C NMR (125 MHz, CDCl_3_) *δ* 171.6, 163.7, 141.1, 136.2, 134.6, 133.5, 131.9, 130.3, 129.8, 129.4129.3, 128.9, 128.6, 126.8, 124.1, 122.6, 108.2, 61.5, 21.5; HRMS (ESI-TOF) *m/z*: [M + H]^+^ Calcd for C_22_H_18_NO_3_ 344.1281; Found 344.1277.

*(7-Oxo-5-phenyl-6,7-dihydrothieno [2,3-c]pyridin-4-yl)methyl acetate (**3ya**)*. This compound was purified by column chromatography (ethyl acetate/petroleum ether = 1:6) to provide a white solid in 51% yield (30 mg); mp 158–159 °C; ^1^H NMR (500 MHz, CDCl_3_) *δ* 9.64 (s, 1H), 7.80 (d, *J* = 5.0 Hz, 1H), 7.53–7.51 (m, 5H), 7.38 (d, *J* = 5.0 Hz, 1H), 5.13 (s, 2H), 2.09 (s, 3H); ^13^C NMR (100 MHz, CDCl_3_) *δ* 171.0, 158.9, 147.1, 142.8, 134.4, 133.1, 130.1, 129.1, 129.0, 124.4, 123.5, 108.0, 61.2, 21.1; HRMS (ESI-TOF) *m/z*: [M + H]^+^ Calcd for C_16_H_14_NO_3_S 300.0689; Found 300.0684.

*(1-Oxo-3-(p-tolyl)-1,2-dihydroisoquinolin-4-yl)methyl acetate (**3ab**)*. This compound was purified by column chromatography (ethyl acetate/petroleum ether = 1:6) to provide a white solid in 72% yield (44 mg); mp 163–164 °C; ^1^H NMR (500 MHz, CDCl_3_) *δ* 9.67 (s, 1H), 8.39 (d, *J* = 8.0 Hz, 1H), 7.76–7.68 (m, 2H), 7.54 –7.51 (m, 1H), 7.39 (d, *J* = 8.0 Hz, 2H), 7.30 (d, *J* = 8.0 Hz, 2H), 5.15 (s, 2H), 2.43 (s, 3H), 2.11 (s, 3H); ^13^C NMR (125 MHz, CDCl_3_) *δ* 171.4, 163.2, 142.5, 140.5, 138.0, 133.6, 131.3, 130.0, 129.2, 128.3, 127.1, 125.8, 123.8, 108.1, 61.2, 21.8, 21.5; HRMS (ESI-TOF) *m/z*: [M + H]^+^ Calcd for C_19_H_18_NO_3_ 308.1281; Found 308.1279.

*(3-(4-Ethylphenyl)-1-oxo-1,2-dihydroisoquinolin-4-yl)methyl acetate (**3ac**)*. This compound was purified by column chromatography (ethyl acetate/petroleum ether = 1:6) to provide a white solid in 67% yield (43 mg); mp 165–166 °C; ^1^H NMR (500 MHz, CDCl_3_) *δ* 9.94 (s, 1H), 8.38 (d, *J* = 8.0 Hz, 1H), 7.76–7.68 (m, 2H), 7.51 (m, 1H), 7.43 (d, *J* = 8.0 Hz, 2H), 7.33 (d, *J* = 8.0 Hz, 2H), 5.17 (s, 2H), 2.73 (q, *J* = 5.0 Hz, 2H), 2.11 (s, 3H), 1.30 (t, *J* = 5.0 Hz, 3H); ^13^C NMR (125 MHz, CDCl_3_) *δ* 171.5, 163.5, 146.6, 142.7, 138.1, 133.6, 131.5, 129.4, 128.8, 128.3, 127.1, 125.8, 123.7, 108.0, 61.3, 29.1, 21.5, 15.7; HRMS (ESI-TOF) *m/z*: [M + H]^+^ Calcd for C_20_H_20_NO_3_ 322.1438; Found 322.1436.

*(3-(4-(tert-Butyl)phenyl)-1-oxo-1,2-dihydroisoquinolin-4-yl)methyl acetate (**3ad**)*. This compound was purified by column chromatography (ethyl acetate/petroleum ether = 1:6) to provide a white solid in 49% yield (34 mg); mp 171–172 °C; ^1^H NMR (500 MHz, CDCl_3_) *δ* 8.42 (dd, *J* = 10.0, 5.0 Hz, 1H), 7.75–7.74 (m, 1H), 7.69 (d, *J* = 5.0 Hz, 1H), 7.53–7.51(m, 3H), 7.43 (dd, *J* = 5.0, 2.0 Hz, 2H), 5.18 (s, 2H), 2.13 (s, 3H), 1.38 (s, 11H); ^13^C NMR (125 MHz, CDCl_3_) *δ* 171.5, 162.9, 153.6, 142.4, 138.0, 133.7, 131.3, 129.0, 128.3, 127.2, 126.4, 125.9, 123.8, 108.0, 61.3, 35.3, 31.6, 21.5; HRMS (ESI-TOF) *m/z*: [M + H]^+^ Calcd for C_22_H_24_NO_3_ 350.1751; Found 350.1754.

*(3-(4-Methoxyphenyl)-1-oxo-1,2-dihydroisoquinolin-4-yl)methyl acetate (**3ae**)*. This compound was purified by column chromatography (ethyl acetate/petroleum ether = 1:6) to provide a white solid in 76% yield (49 mg); mp 174–175 °C; ^1^H NMR (500 MHz, CDCl_3_) *δ* 8.77 (s, 1H), 8.45 (d, *J* = 7.5 Hz, 1H), 7.77–7.68 (m, *2*H), 7.56–7.50 (m, 1H), 7.42 (d, *J* = 8.5 Hz, 2H), 7.02 (d, *J* = 8.5 Hz, 2H), 5.16 (s, 2H), 3.88 (s, 3H), 2.12 (s, 3H); ^13^C NMR (125 MHz, CDCl_3_) *δ* 171.4, 163.0, 161.2, 142.0, 138.0, 133.7 130.6, 128.3, 127.2, 126.5, 125.8, 123.8, 114.9, 108.1, 61.3, 55.8, 21.4; HRMS (ESI-TOF) *m/z*: [M + H]^+^ Calcd for C_19_H_18_NO_4_ 324.1230; Found 324.1225.

*(3-(4-Fluorophenyl)-1-oxo-1,2-dihydroisoquinolin-4-yl)methyl acetate (**3af**)*. This compound was purified by column chromatography (ethyl acetate/petroleum ether = 1:6) to provide a white solid in 87% yield (54 mg); mp 193–194 °C; ^1^H NMR (500 MHz, CDCl_3_) *δ* 10.50 (s, 1H), 8.35 (d, *J* = 8.0 Hz, 1H), 7.78–7.70 (m, 2H), 7.59–7.47 (m, 3H), 7.22 (dd, *J* = 8.5, 8.5 Hz, 2H), 5.14 (s, 2H), 2.11 (s, 3H); ^13^C NMR (125 MHz, CDCl_3_) *δ* 171.1, 164.1 (d, *J* = 243.8 Hz), 163.4, 141.2, 137.5, 133.5, 131.4 (d, *J* = 8.8 Hz), 123.0, 128.0, 127.2, 125.6, 123.6, 116.2 (d, *J* = 21.2 Hz), 108.4, 60.8, 21.2; HRMS (ESI-TOF) *m/z*: [M + H]^+^ Calcd for C_18_H_15_FNO_3_ 312.1030; Found 312.1021.

*(3-(4-Chlorophenyl)-1-oxo-1,2-dihydroisoquinolin-4-yl)methyl acetate (**3ag**)*. This compound was purified by column chromatography (ethyl acetate/petroleum ether = 1:6) to provide a white solid in 84% yield (55 mg); mp 184–185 °C; ^1^H NMR (500 MHz, CDCl_3_) *δ* 10.57 (s, 1H), 8.34 (d, *J* = 8.0 Hz, 1H), 7.78–7.70 (m, 2H), 7.57–7.47 (m, 5H), 5.13 (s, 2H), 2.11 (s, 3H); ^13^C NMR (125 MHz, CDCl_3_) *δ* 171.3, 163.6, 141.3, 137.8, 136.6, 133.8, 132.5, 131.0, 129.5, 128.2, 127.5, 125.8, 123.9, 108.7, 60.9, 21.4; HRMS (ESI-TOF) *m/z*: [M + H]^+^ Calcd for C_18_H_15_ClNO_3_ 328.0735; Found 328.0736.

*(3-(4-Bromophenyl)-1-oxo-1,2-dihydroisoquinolin-4-yl)methyl acetate (**3ah**)*. This compound was purified by column chromatography (ethyl acetate/petroleum ether = 1:6) to provide a white solid in 79% yield (58 mg); mp 178–179 °C; ^1^H NMR (500 MHz, CDCl_3_) *δ* 10.55 (s, 1H), 8.35 (d, *J* = 8.0 Hz, 1H), 7.76 (dd, *J* = 7.5, 7.5 Hz, 1H), 7.71 (d, *J* = 8.0 Hz, 1H), 7.66 (d, *J* = 8.5 Hz, 2H), 7.56 (dd, 7.5, 7.5 Hz, 1H), 7.41(d, *J* = 8.5 Hz, 2H), 5.13 (s, 2H), 2.11 (s, 3H); ^13^C NMR (125 MHz, CDCl_3_) *δ* 171.3, 163.6, 141.3, 137.8, 133.8, 132.9, 132.5, 131.2, 128.3, 127.5, 125.8, 124.9, 123.9, 108.7, 60.9, 21.4; HRMS (ESI-TOF) *m/z*: [M + H]^+^ Calcd for C_18_H_15_BrNO_3_ 372.0230; Found 372.0225.

*(1-Oxo-3-(4-(trifluoromethyl)phenyl)-1,2-dihydroisoquinolin-4-yl)methyl acetate (**3ai**)*. This compound was purified by column chromatography (EtOAc/petroleum ether = 1:6) to provide a white solid in 89% yield (64 mg); mp 195–196 °C; ^1^H NMR (500 MHz, CDCl_3_) *δ* 10.87 (s, 1H), 8.32–8.29 (m, 1H), 7.81–7.69 (m, 6H), 7.59–7.57 (m, 1H), 5.14 (s, 2H), 2.12 (s, 3H); ^13^C NMR (125 MHz, CDCl_3_) *δ* 171.1, 163.5, 141.0, 137.6, 137.5, 133.9, 132.1 (q, *J* = 32.5 Hz), 130.2, 128.2, 127.7, 126.2, 125.9, 124.2 (q, *J* = 276.3 Hz), 108.9, 60.5, 21.2; HRMS (ESI-TOF) *m/z*: [M + H]^+^ Calcd for C_19_H_15_F_3_NO_3_ 362.0999; Found 362.0992.
