# Supplementary material for: Rh-Catalyzed Cascade C-H Activation/Annulation of N-Hydroxybenzamides and Propargylic Acetates for Modular Access to Isoquinolones
Source: Molecules. 2022 Dec 5;27(23):8553. doi: 10.3390/molecules27238553 (PMC9740102; doi:10.3390/molecules27238553)
Supplement: Supplementary file 1 [file molecules-27-08553-s001.zip › molecules-2071503-supplementary.pdf]

Supporting Information

Rh-Catalyzed Cascade C-H Activation/Annulation of *N*-Hydroxybenzamides and Propargylic Acetates for Modular Access to Isoquinolones

Taibei Fang [1,†], Shiwen Zhang [1,†], Qingqing Ye [2,*], Shuwen Kong [1], Tingting Yang [1], Kaijie Tang [1], Xinwei He [1,*] and Yongjia Shang [1,*]

[1] Key Laboratory of Functional Molecular Solids, Ministry of Education, Anhui Laboratory of Molecule-Based Materials (State Key Laboratory Cultivation Base), College of Chemistry and Materials Science, Anhui Normal University, Wuhu 241000, China
[2] Department of Medicine, Chuzhou City Vocation College, Chuzhou 239000, China
* Correspondence: yeqingqing1983@sohu.com (Q.Y.); xinweihe@mail.ahnu.edu.cn (X.H.); shyj@mail.ahnu.edu.cn (Y.S.)
† These authors contributed equally to this work.

Table of contents

1. ^{1}H and ^{13}C NMR spectrum for all compounds---S2-S34

2. X-ray crystal data of compound **3aa**--S35

1. ^{1}H and ^{13}C NMR spectrum for all compounds

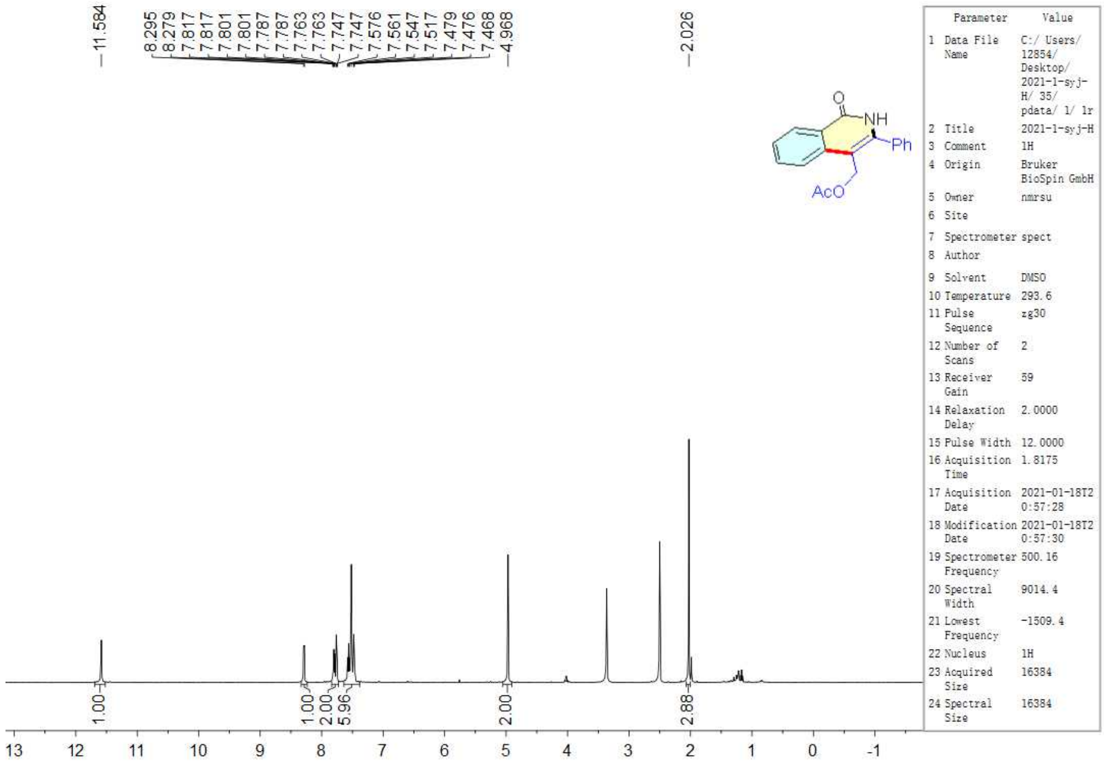

^{1}H NMR spectrum for compound **3aa**

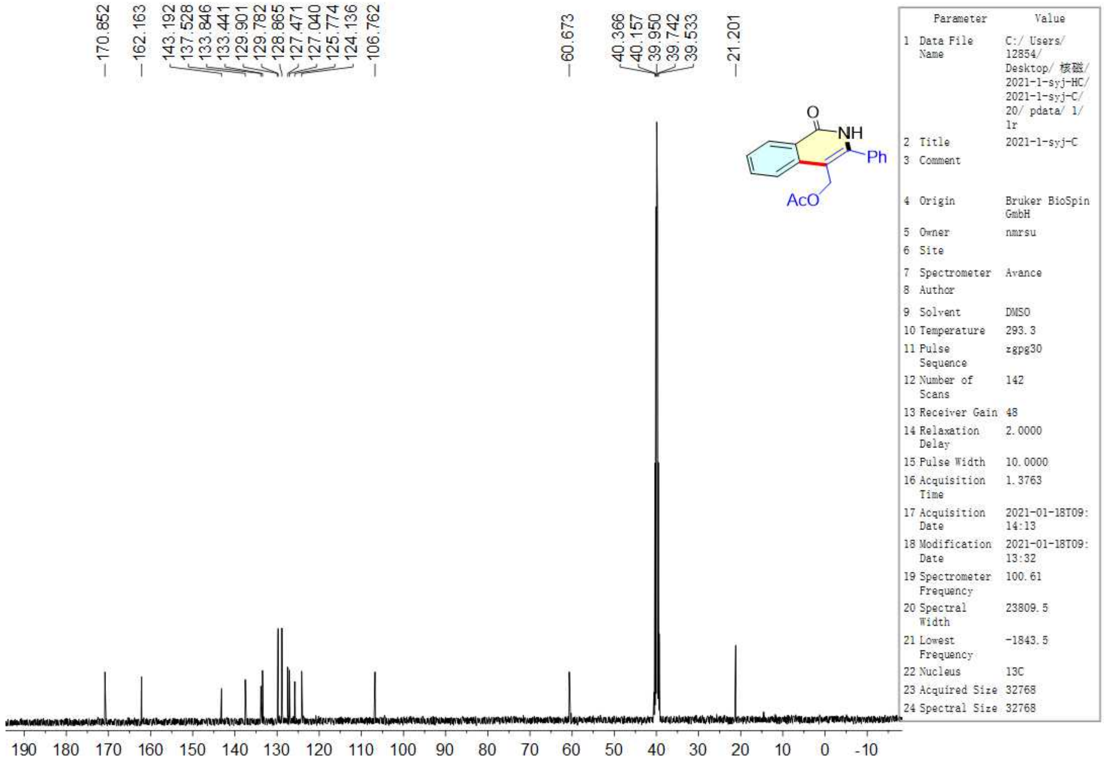

^{13}C NMR spectrum for compound **3aa**

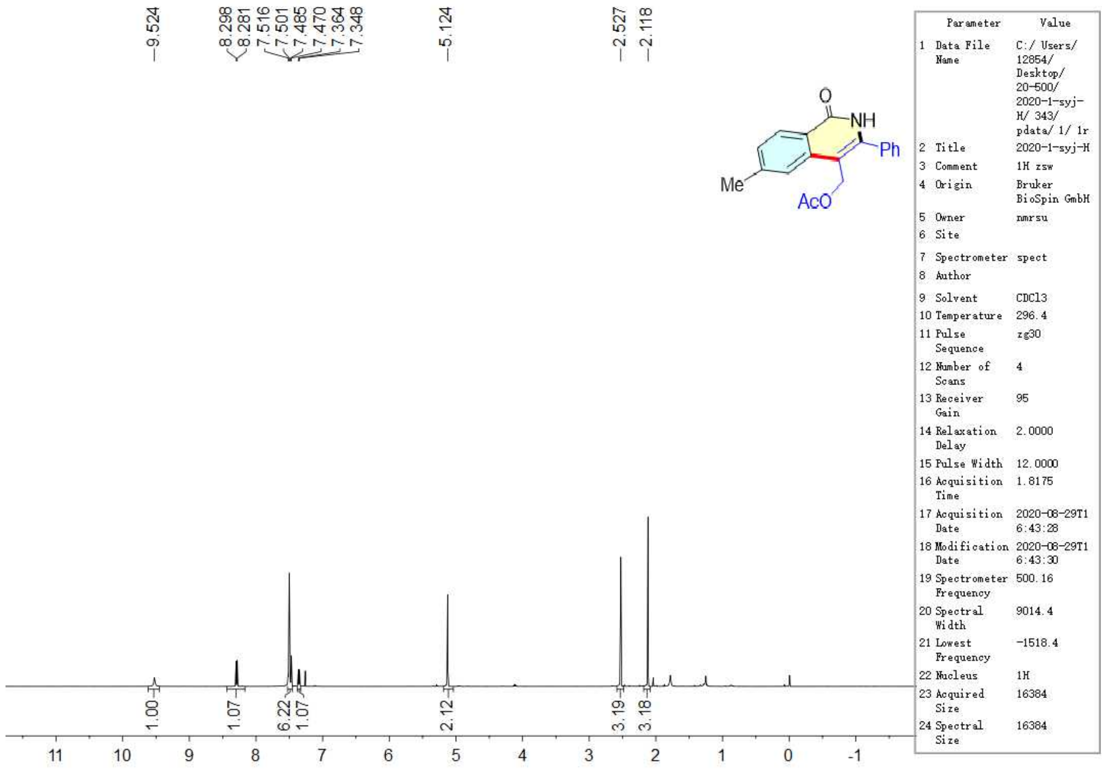

^{1}H NMR spectrum for compound **3ba**

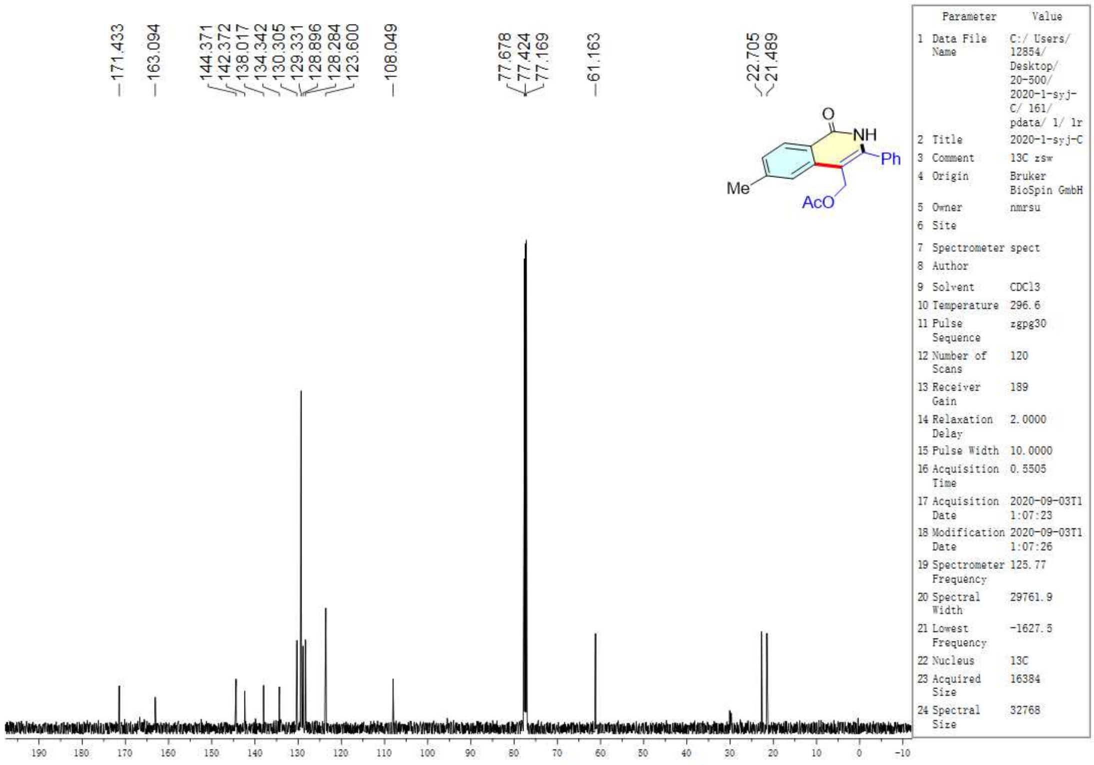

^{13}C NMR spectrum for compound **3ba**

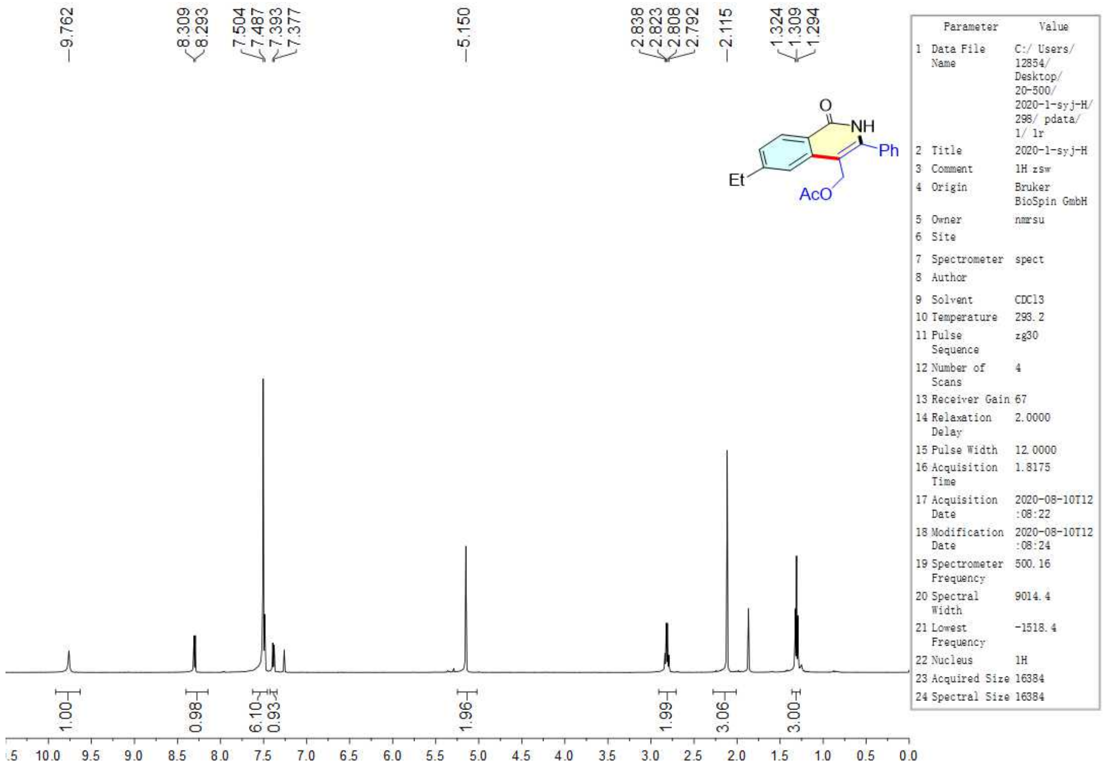

^{1}H NMR spectrum for compound **3ca**

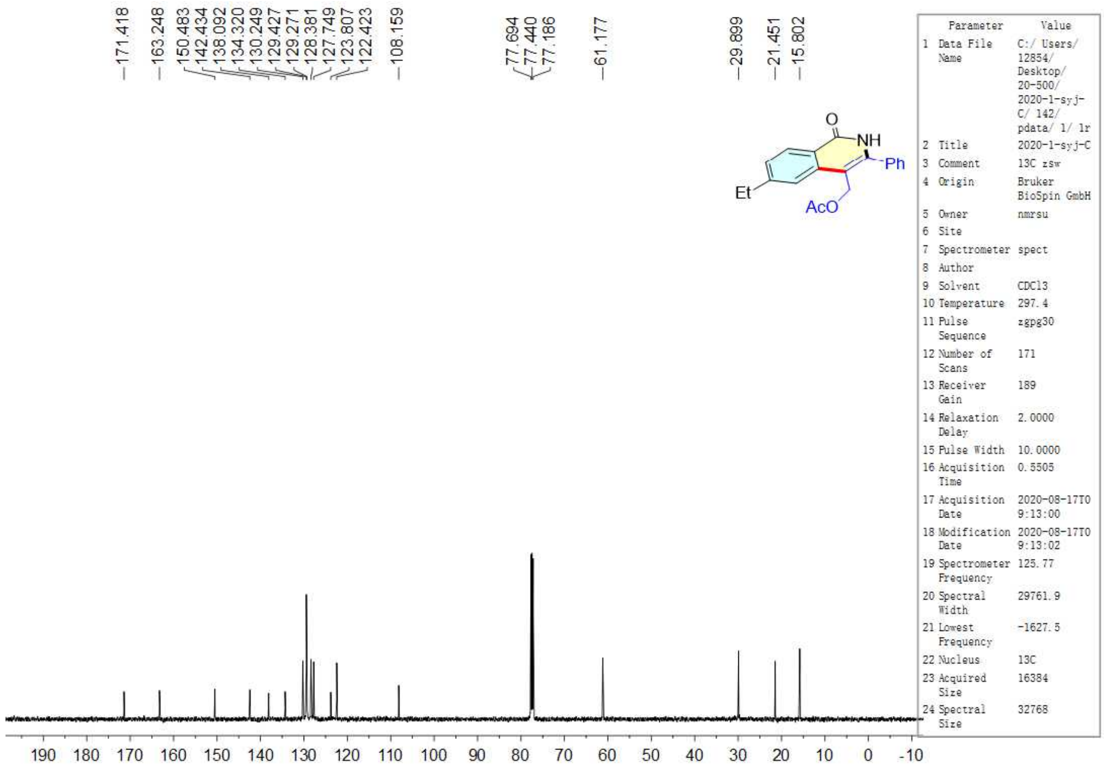

^{13}C NMR spectrum for compound **3ca**

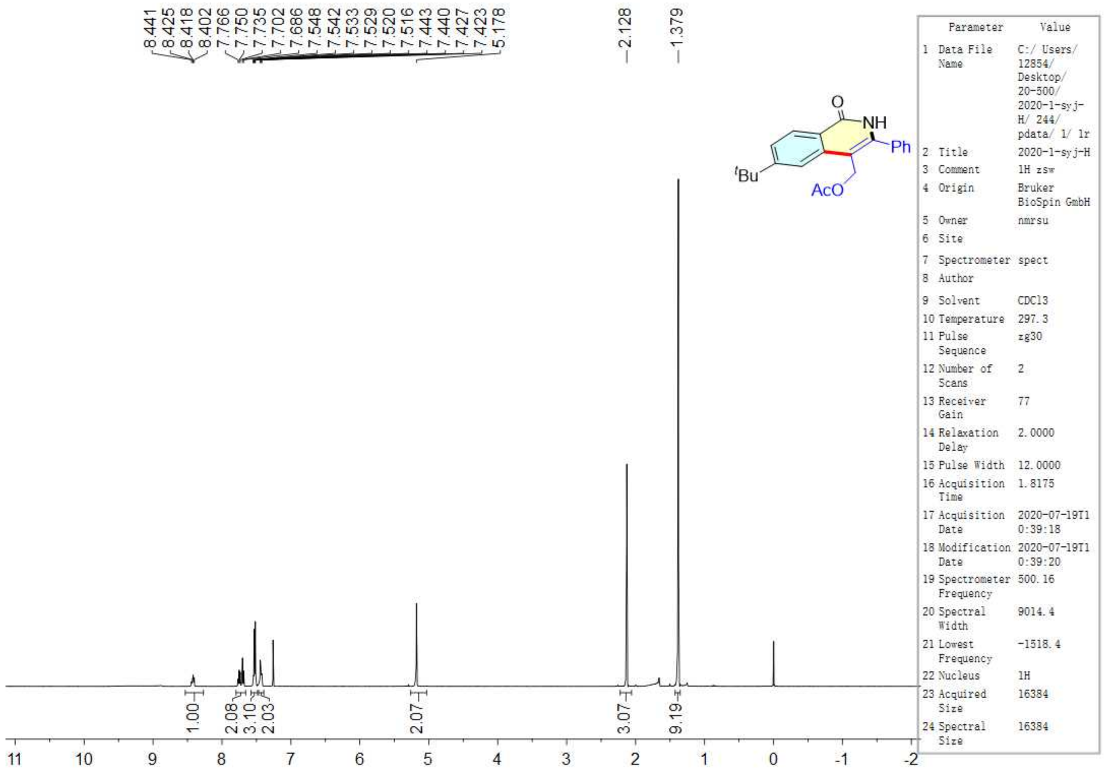

^{1}H NMR spectrum for compound **3da**

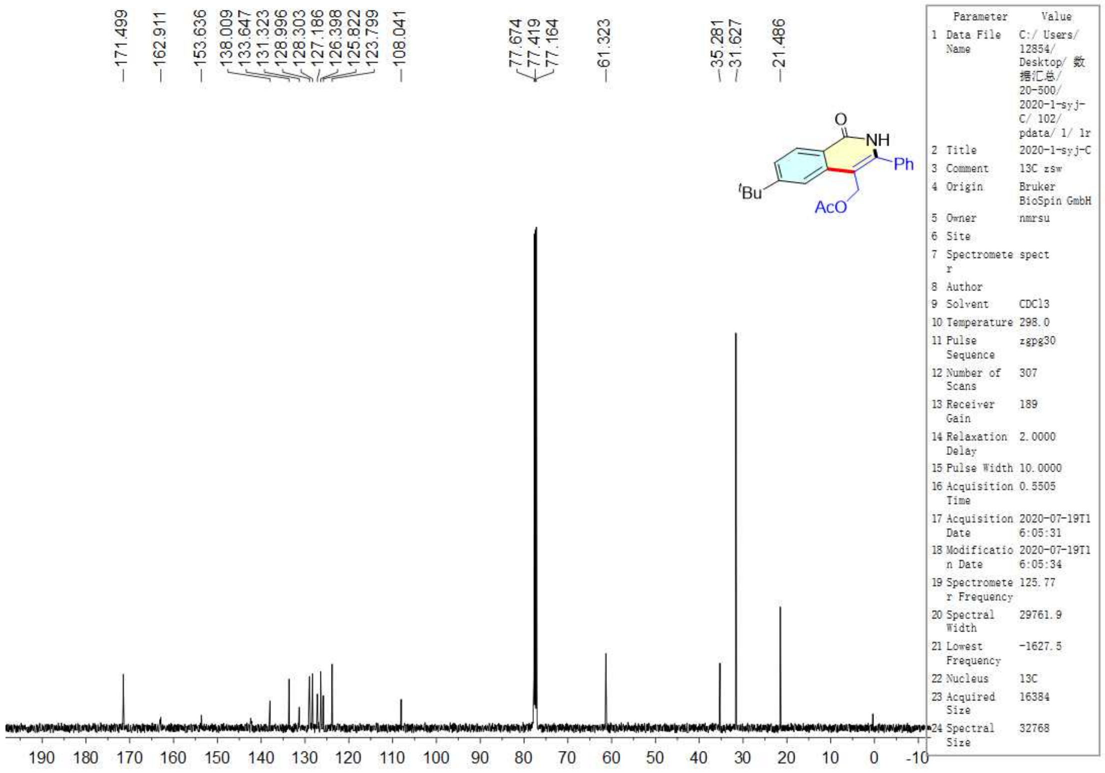

^{13}C NMR spectrum for compound **3da**

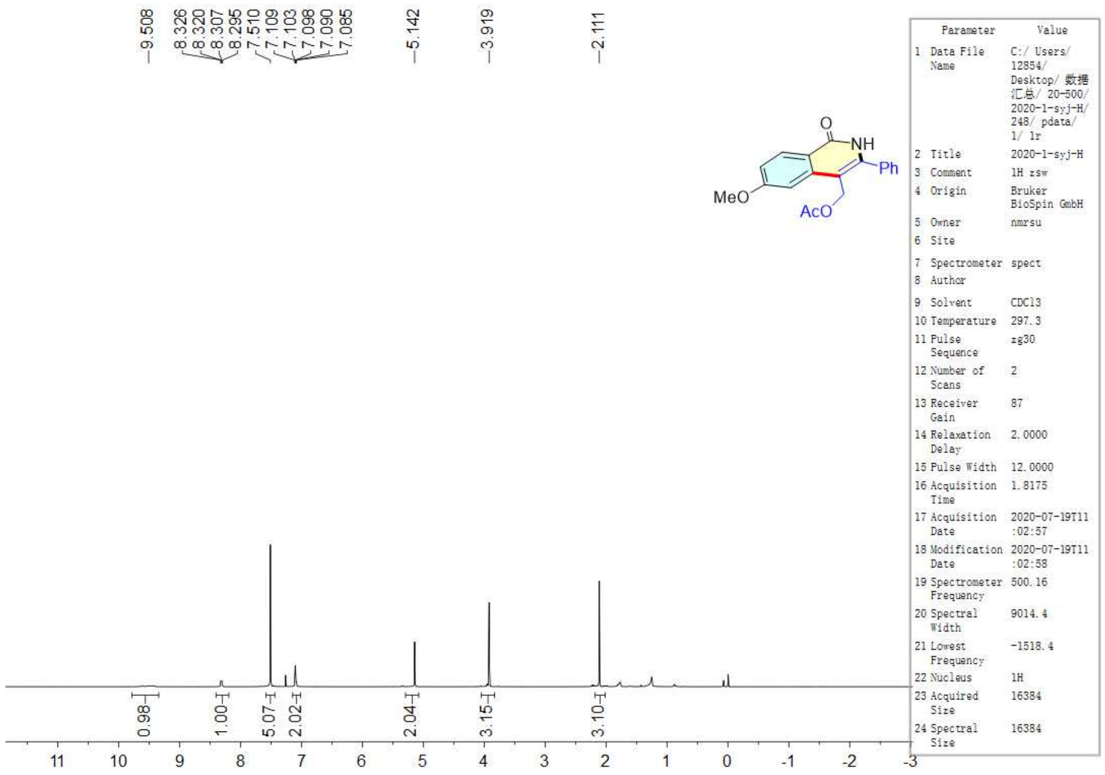

^{1}H NMR spectrum for compound **3ea**

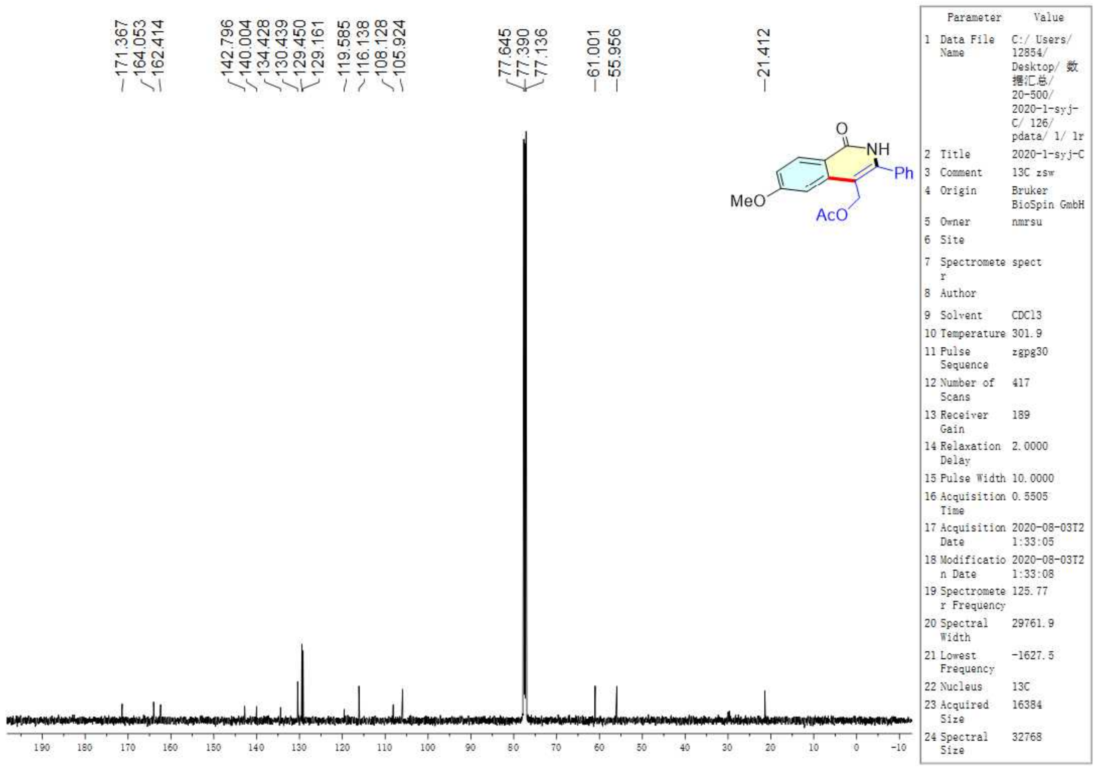

^{13}C NMR spectrum for compound **3ea**

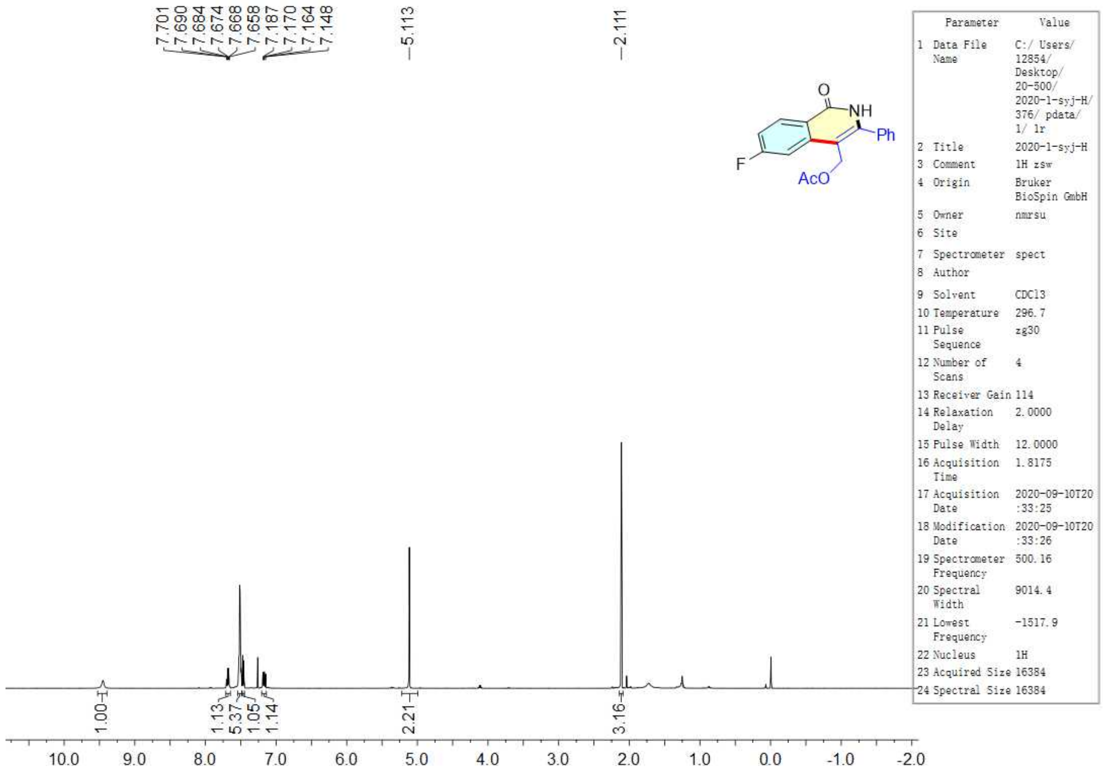

^{1}H NMR spectrum for compound **3fa**

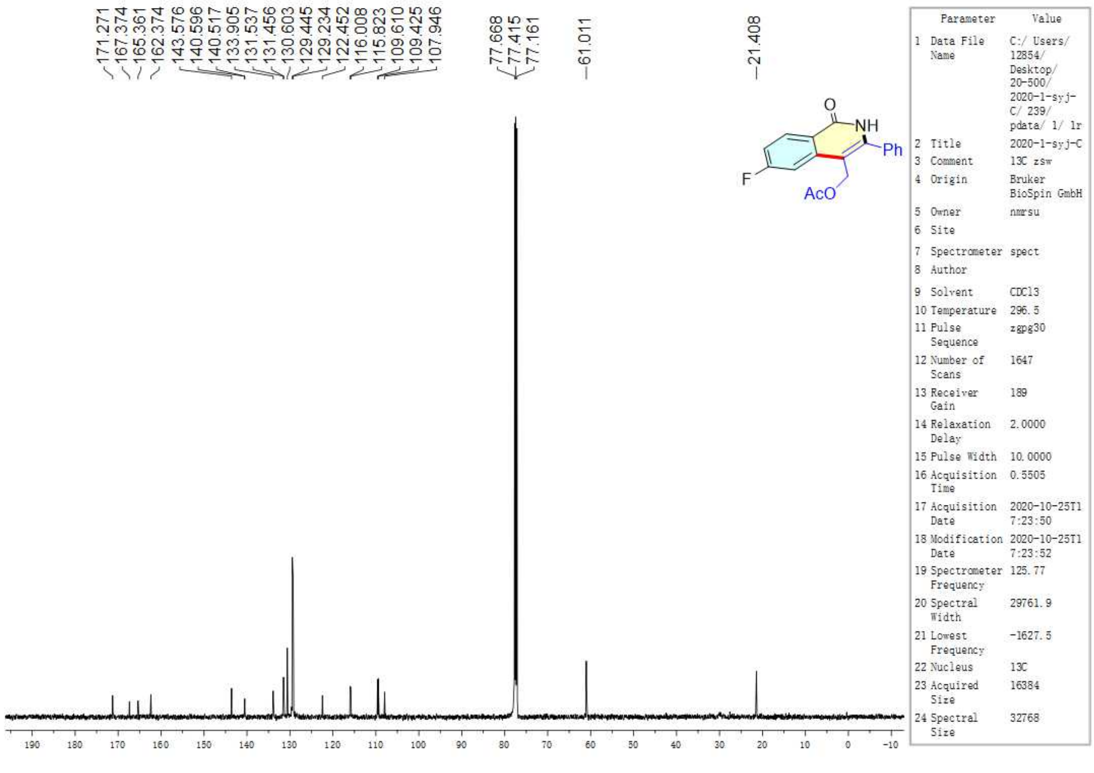

^{13}C NMR spectrum for compound **3fa**

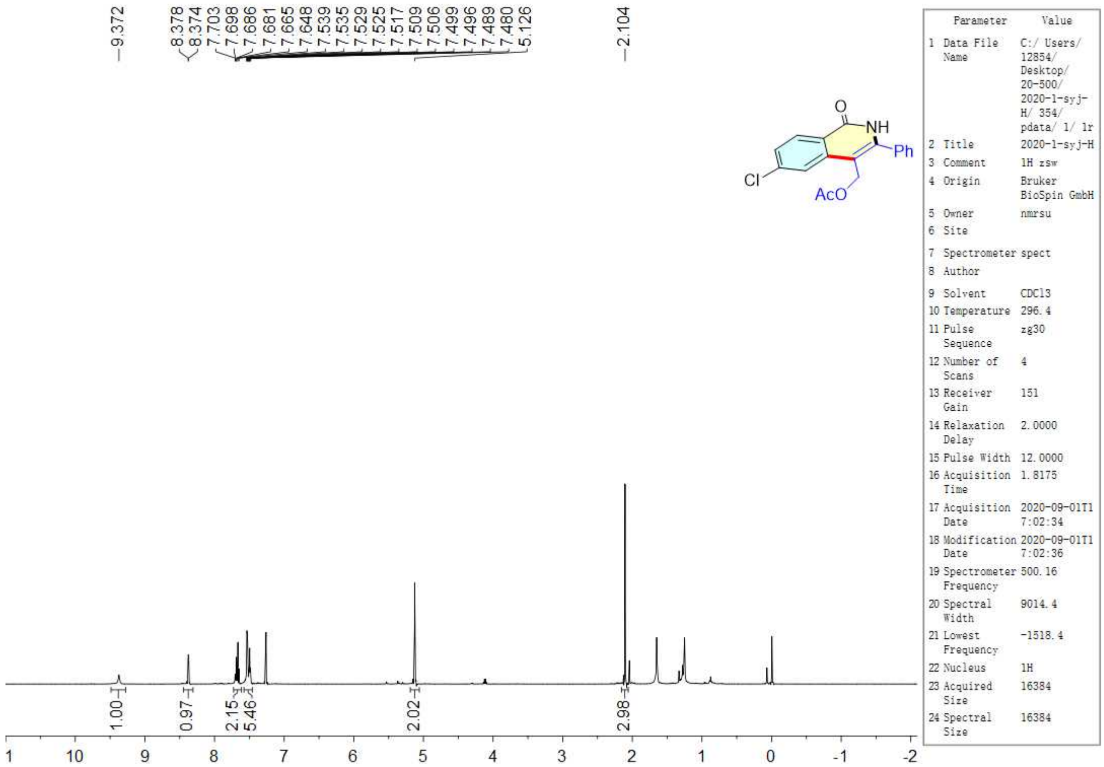

^{1}H NMR spectrum for compound **3ga**

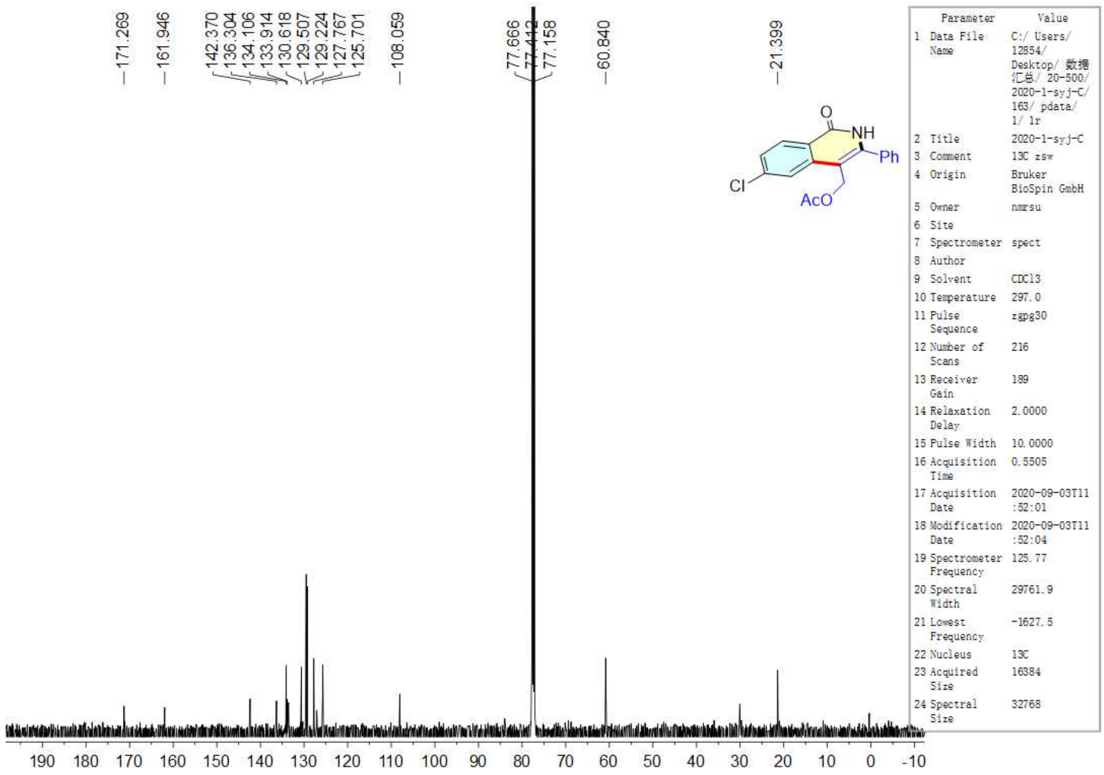

^{13}C NMR spectrum for compound **3ga**

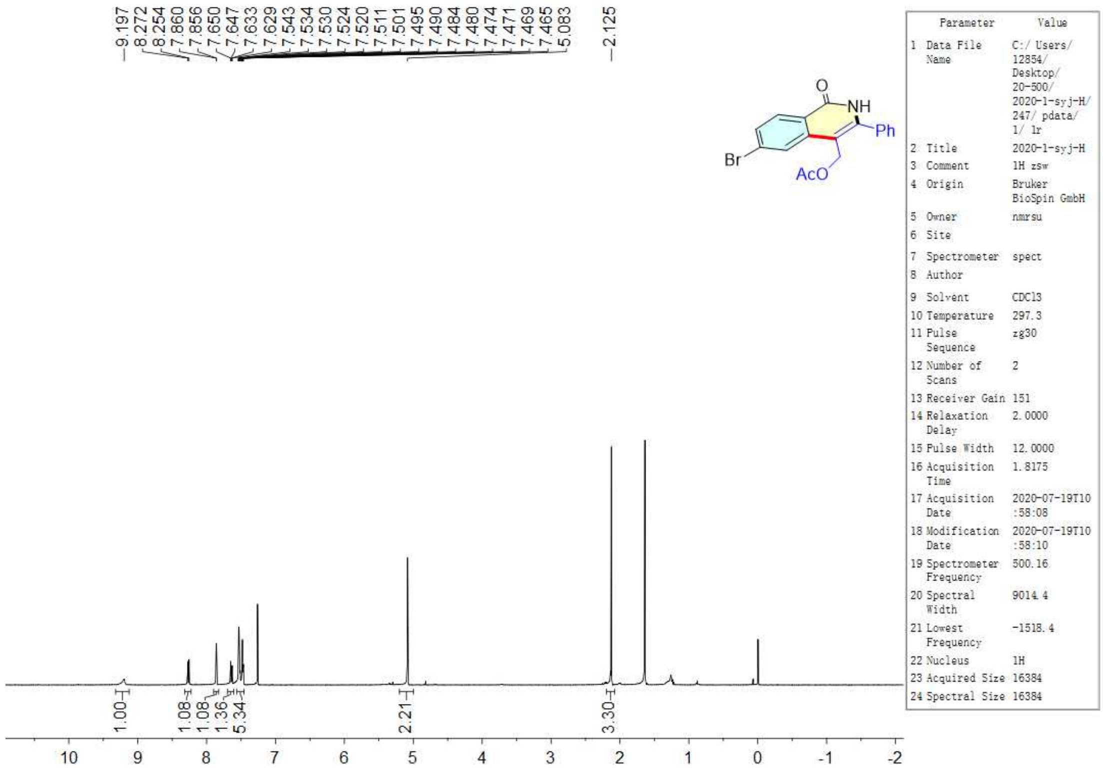

^{1}H NMR spectrum for compound **3ha**

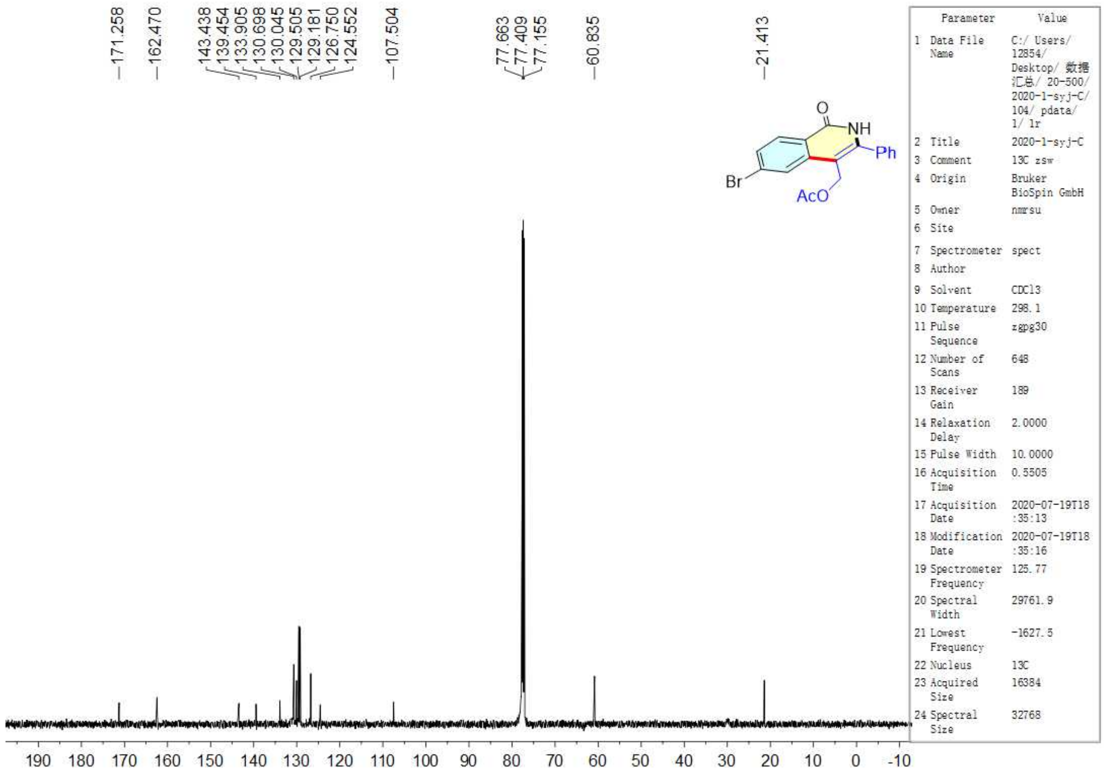

^{13}C NMR spectrum for compound **3ha**

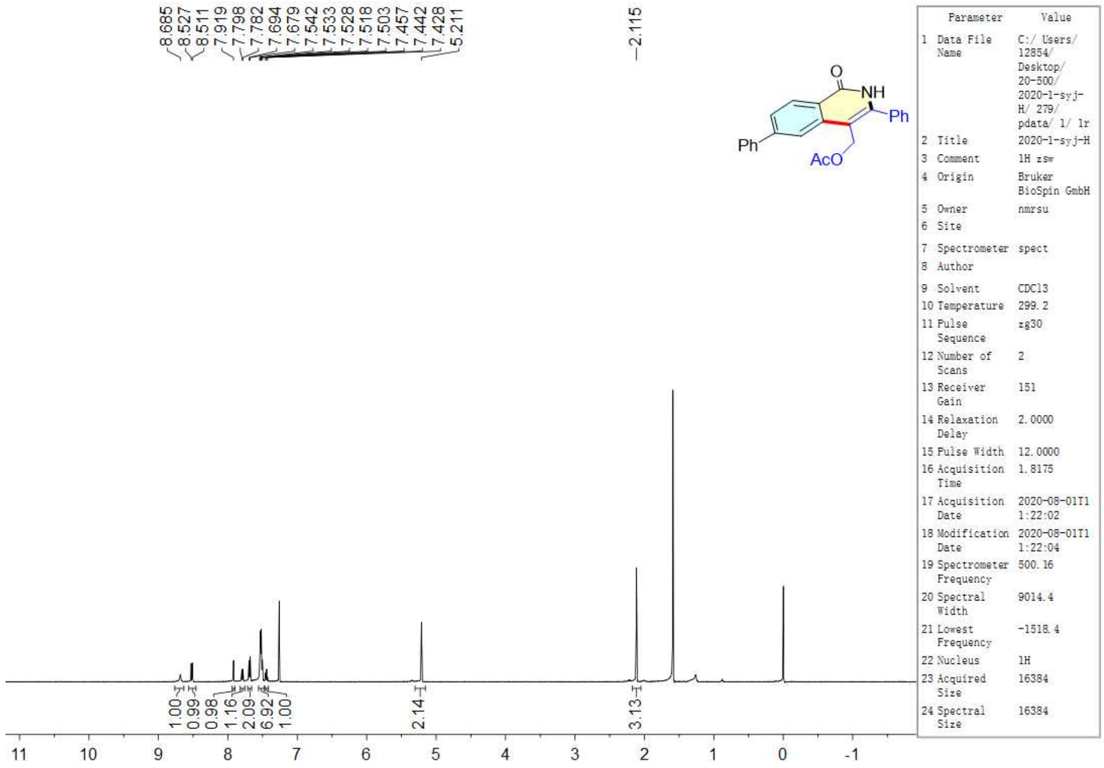

^{1}H NMR spectrum for compound **3ia**

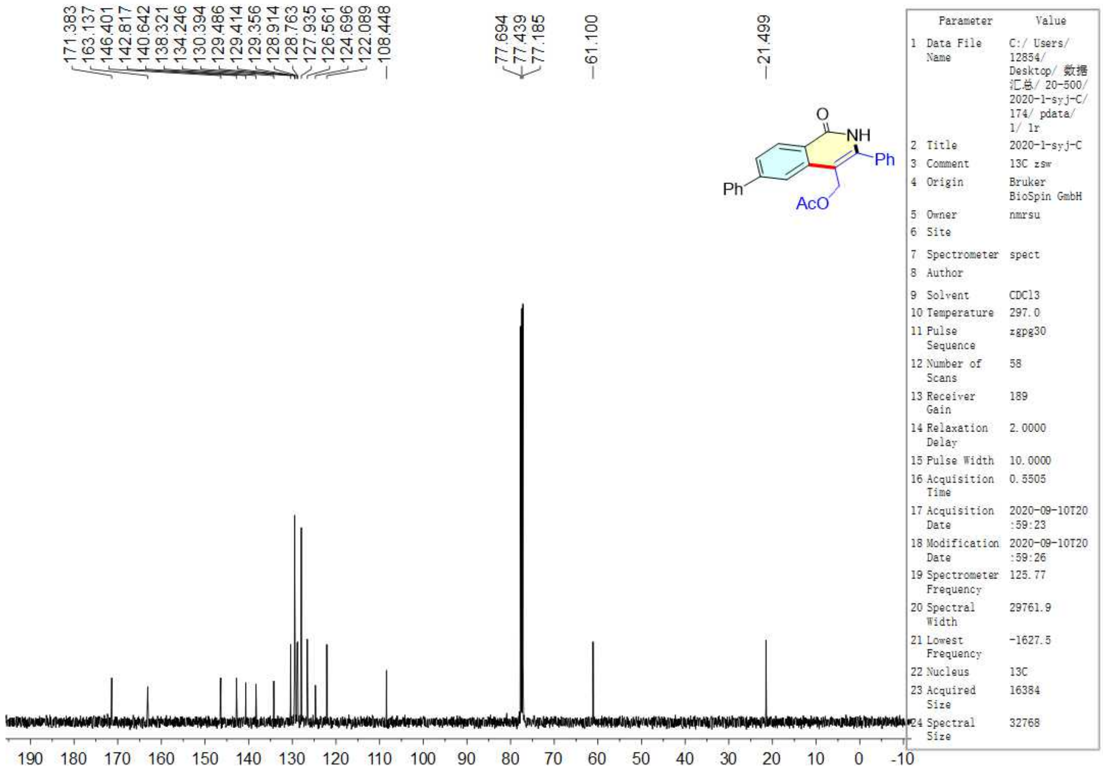

^{13}C NMR spectrum for compound **3ia**

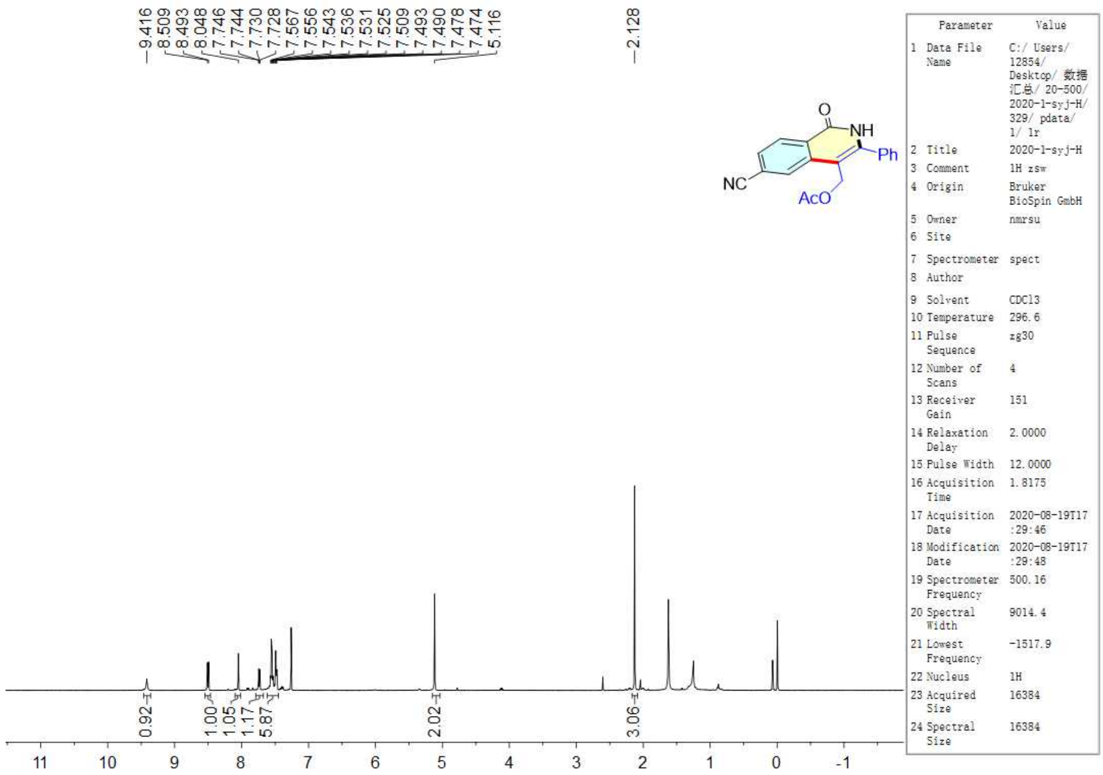

^{1}H NMR spectrum for compound **3ja**

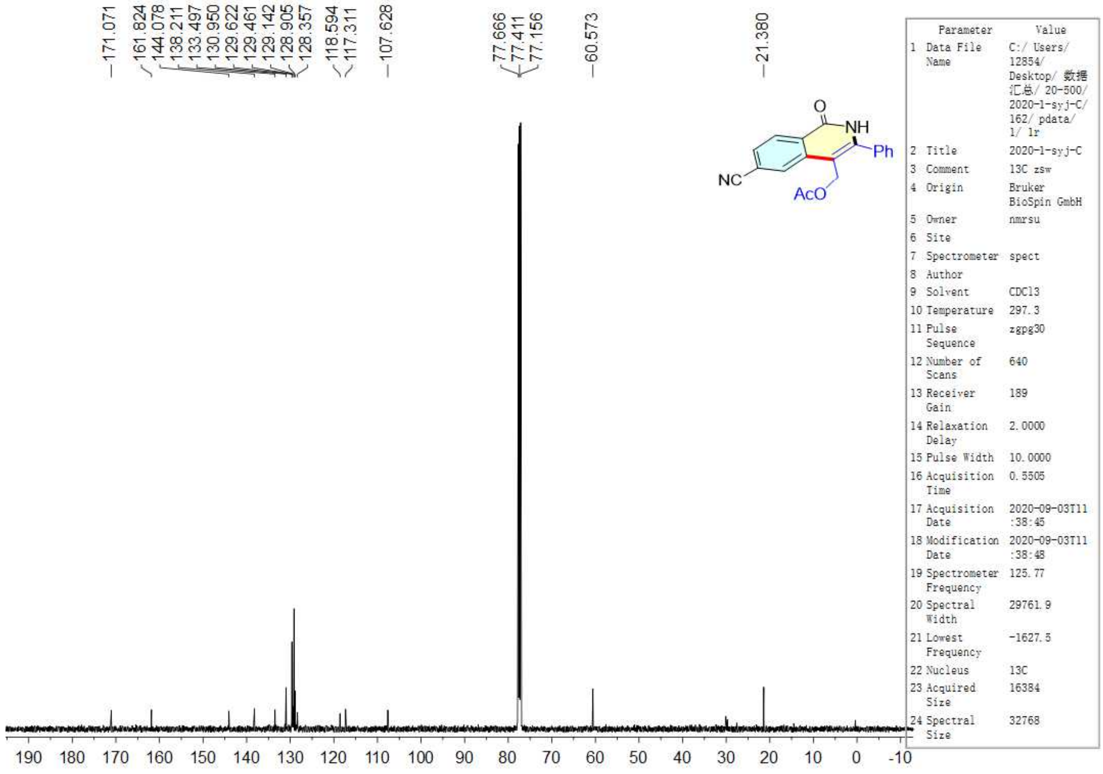

^{13}C NMR spectrum for compound **3ja**

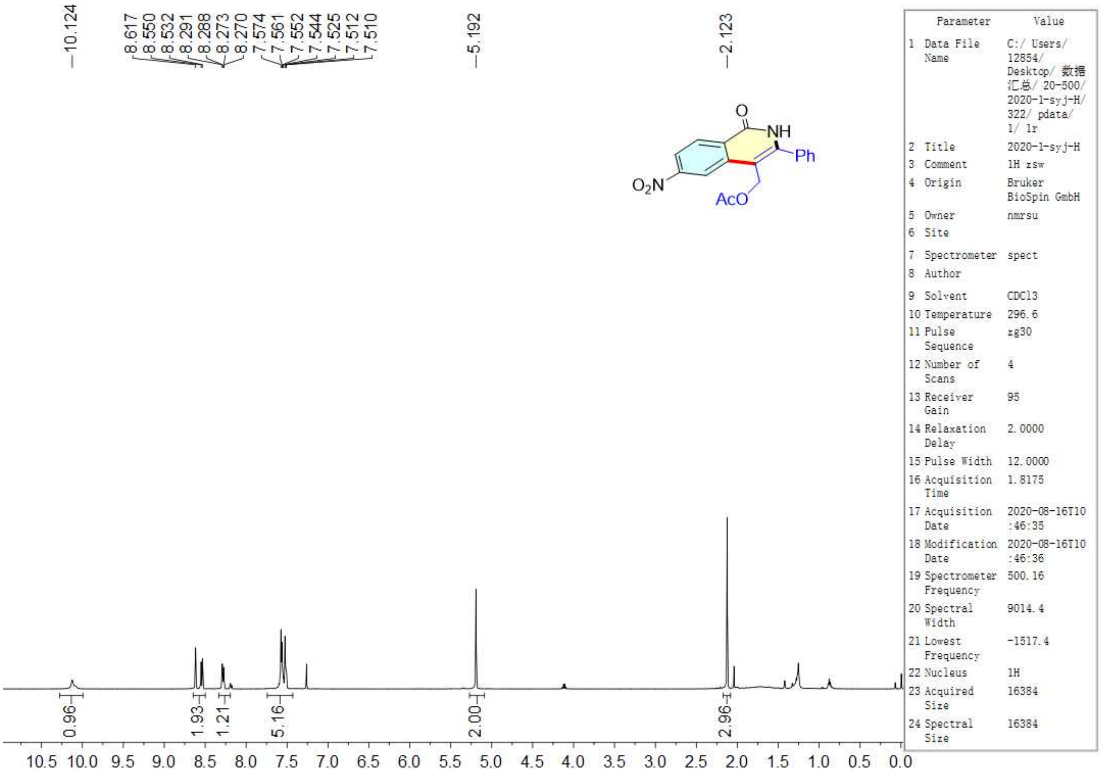

^{1}H NMR spectrum for compound **3ka**

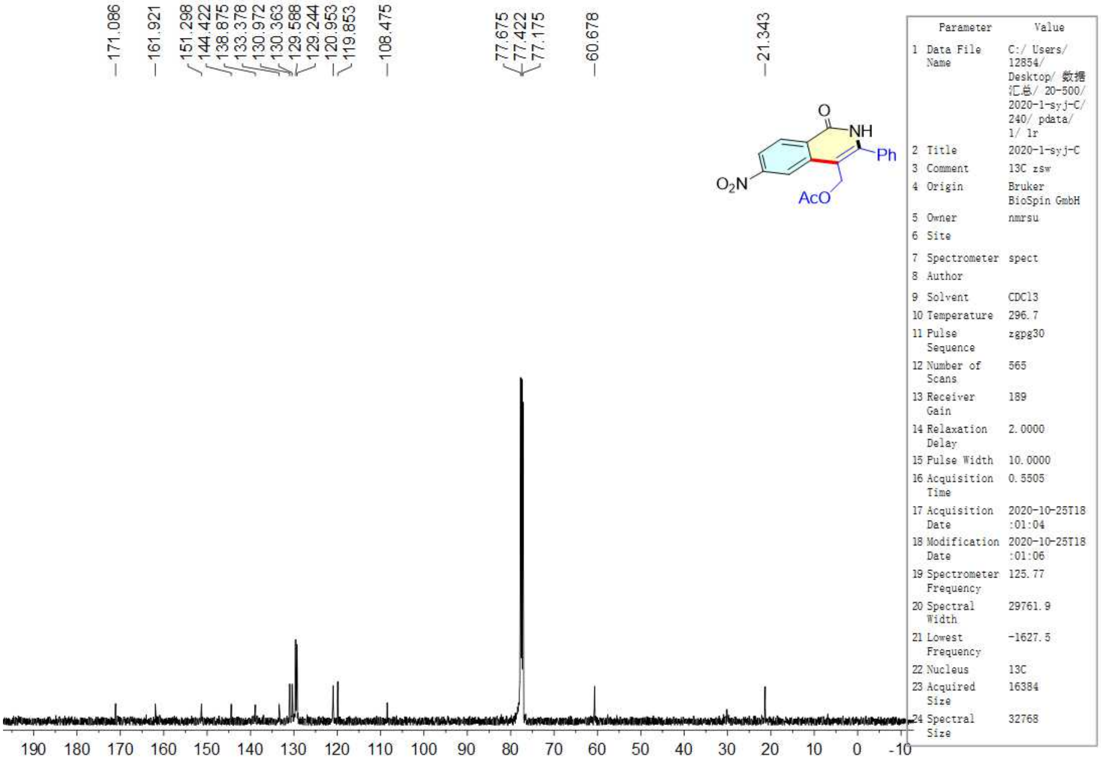

^{13}C NMR spectrum for compound **3ka**

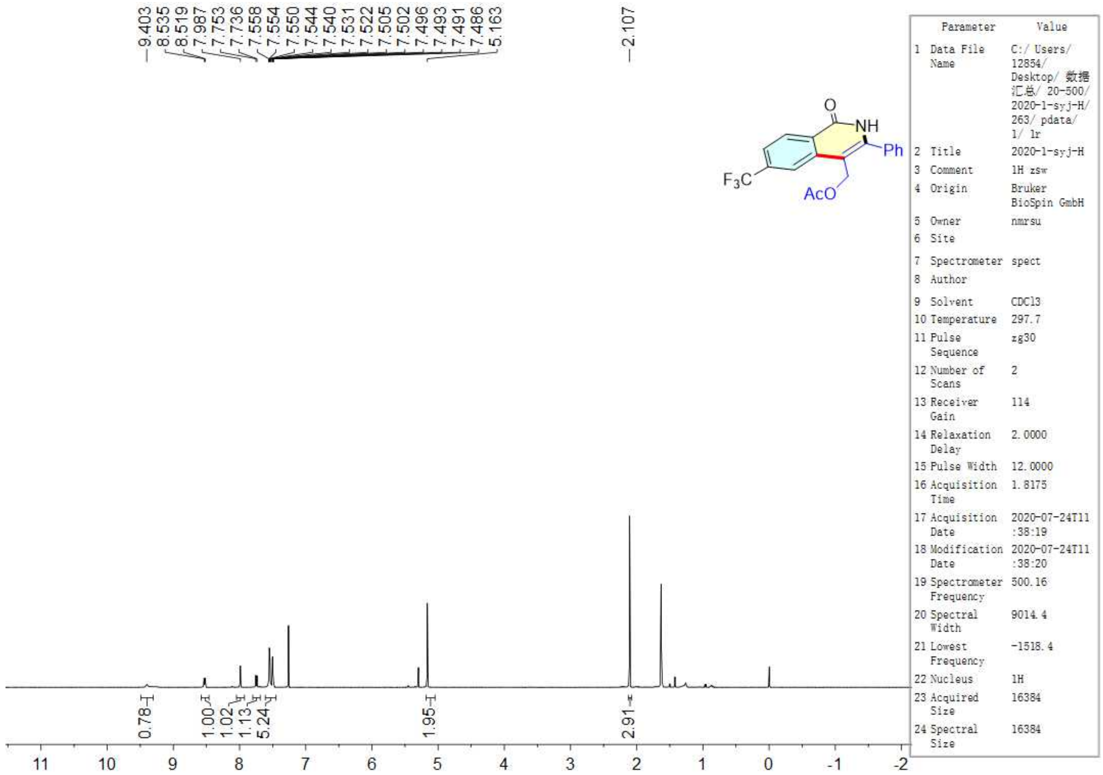

^{1}H NMR spectrum for compound **3la**

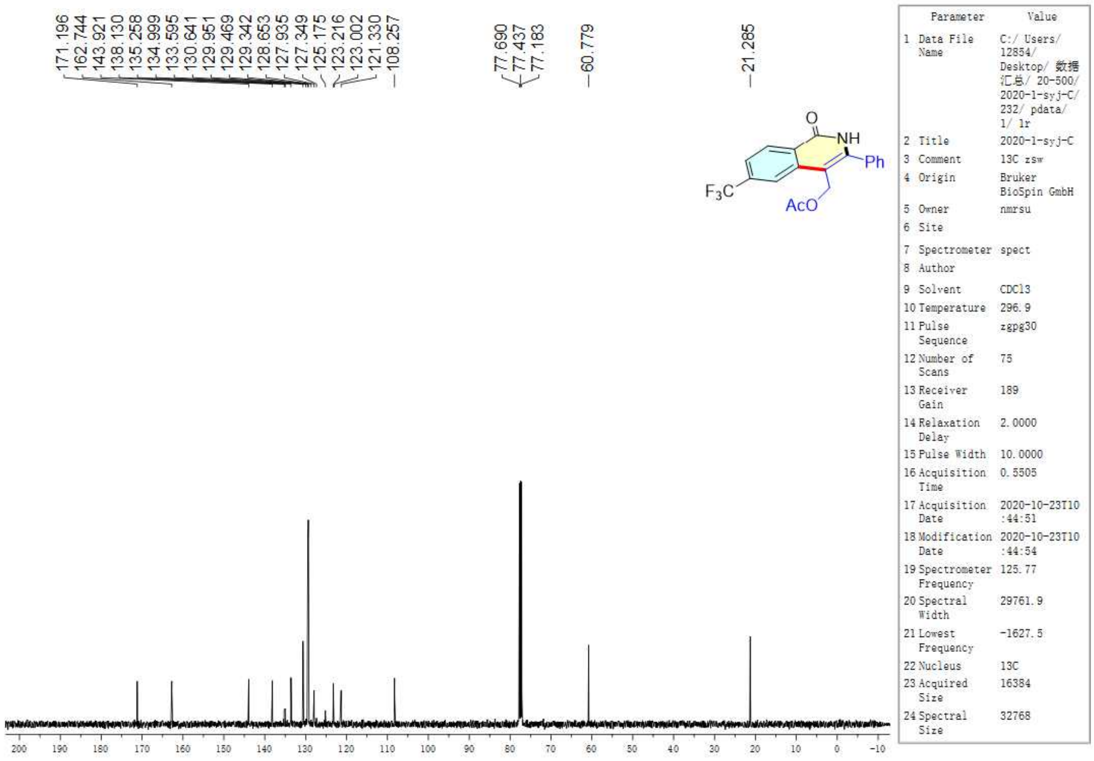

^{13}C NMR spectrum for compound **3la**

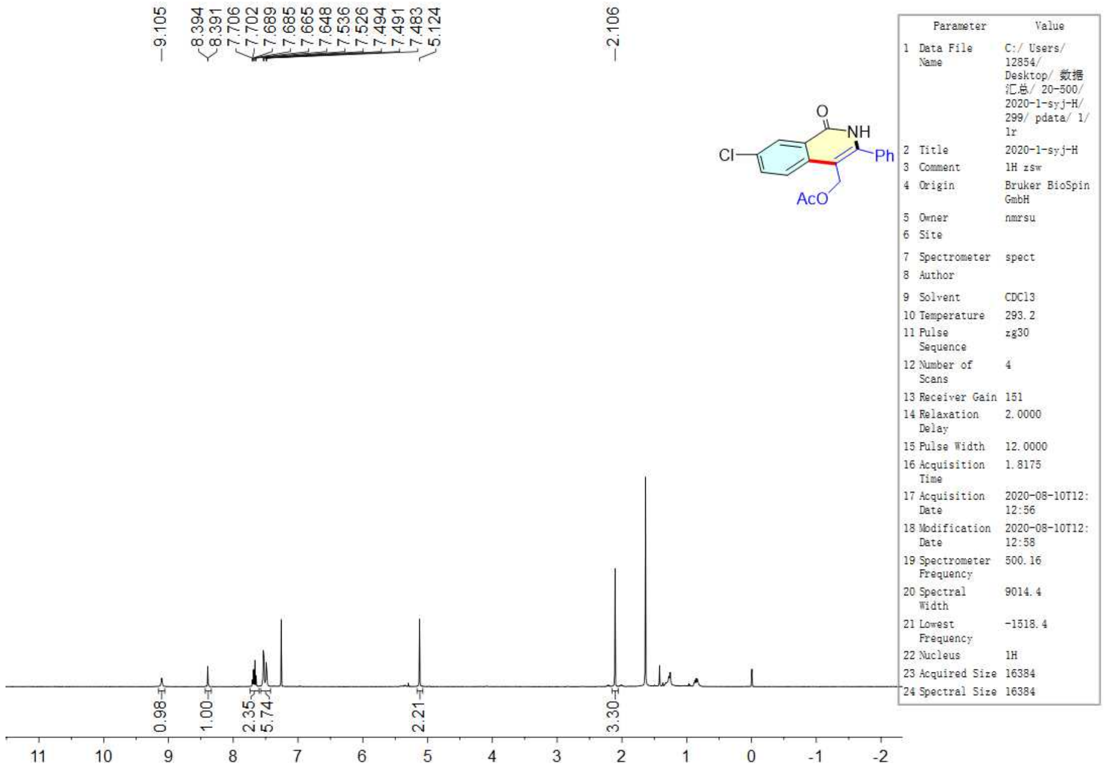

^{1}H NMR spectrum for compound **3ma**

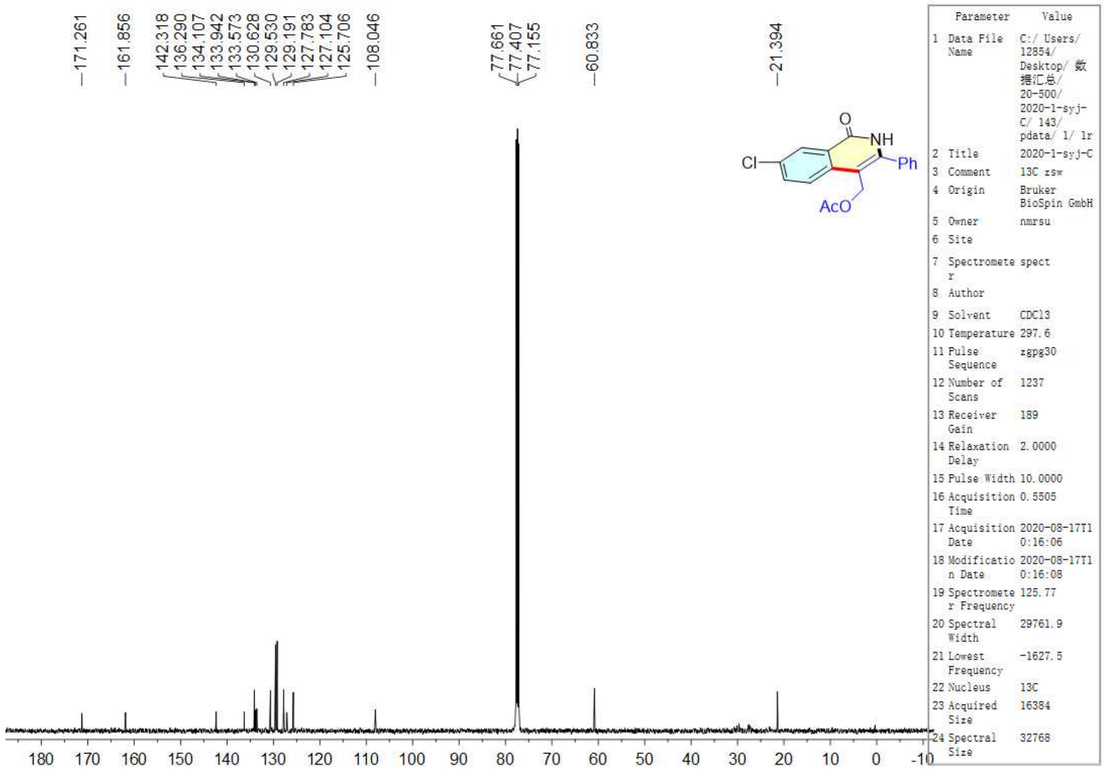

^{13}C NMR spectrum for compound **3ma**

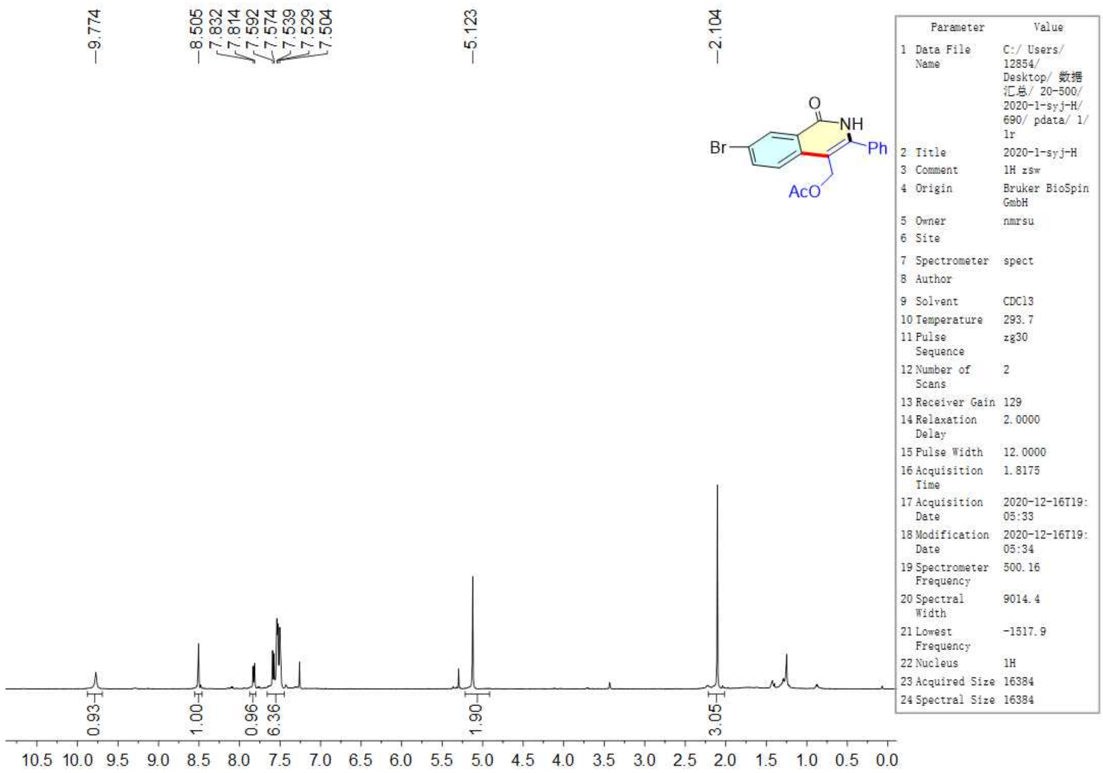

^{1}H NMR spectrum for compound **3na**

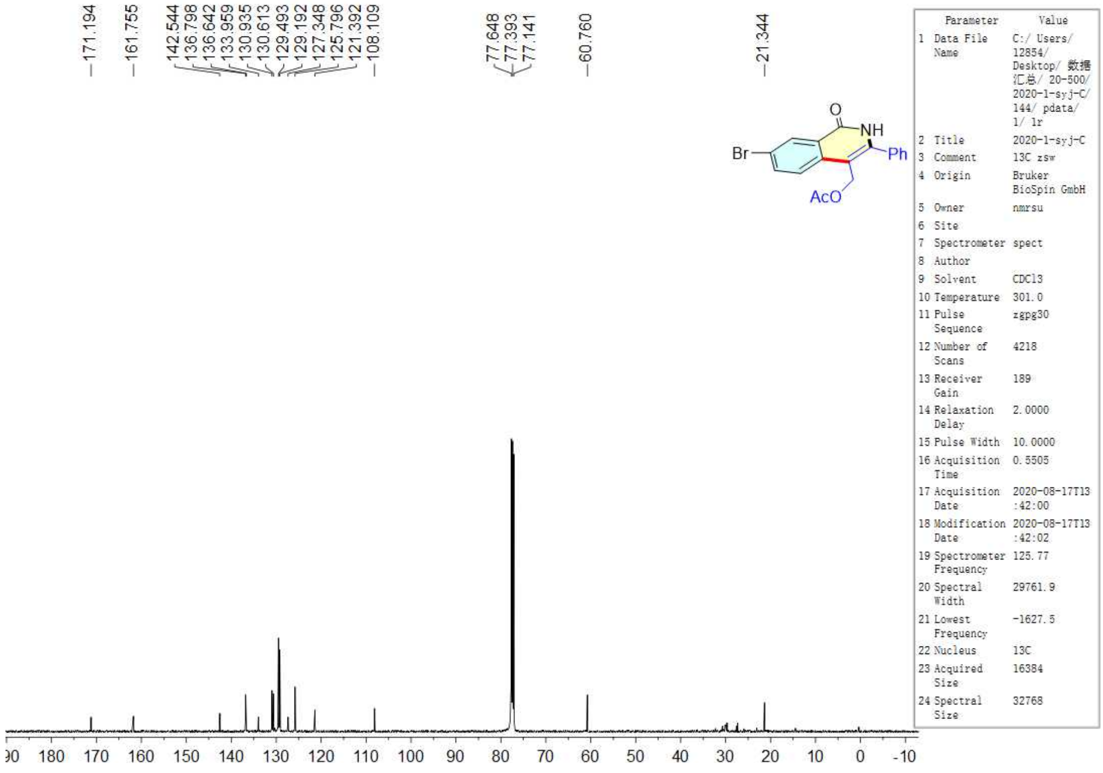

^{13}C NMR spectrum for compound **3na**

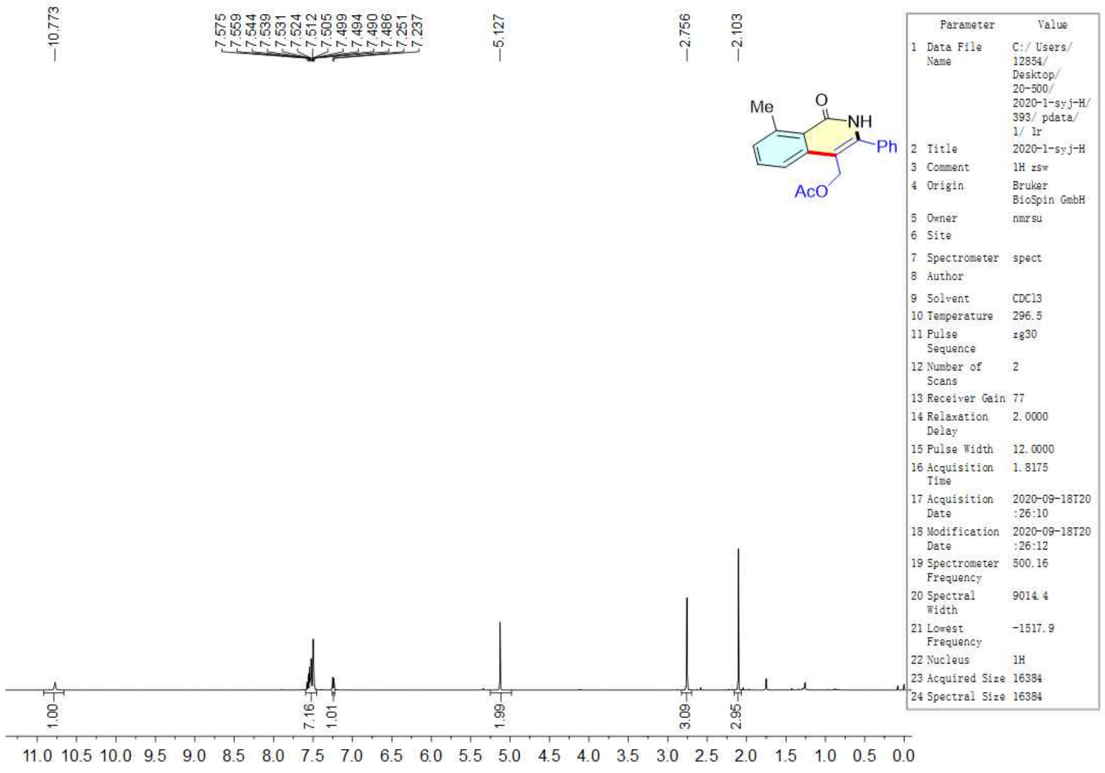

^{1}H NMR spectrum for compound **3oa**

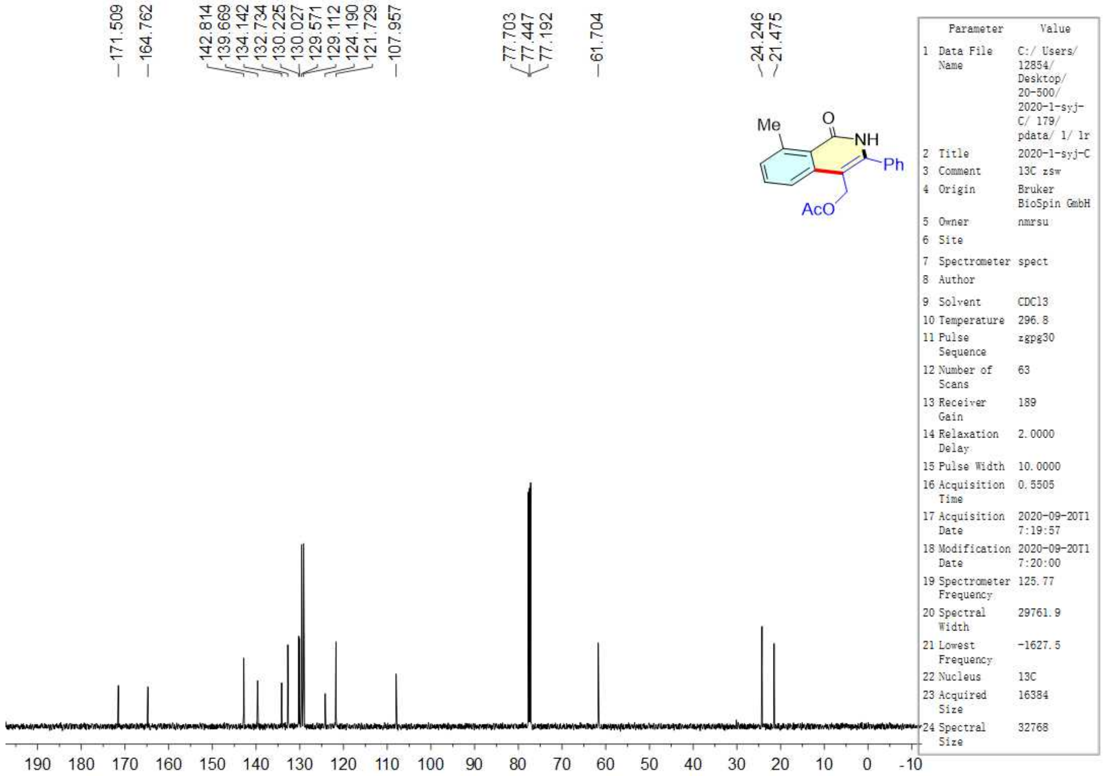

^{13}C NMR spectrum for compound **3oa**

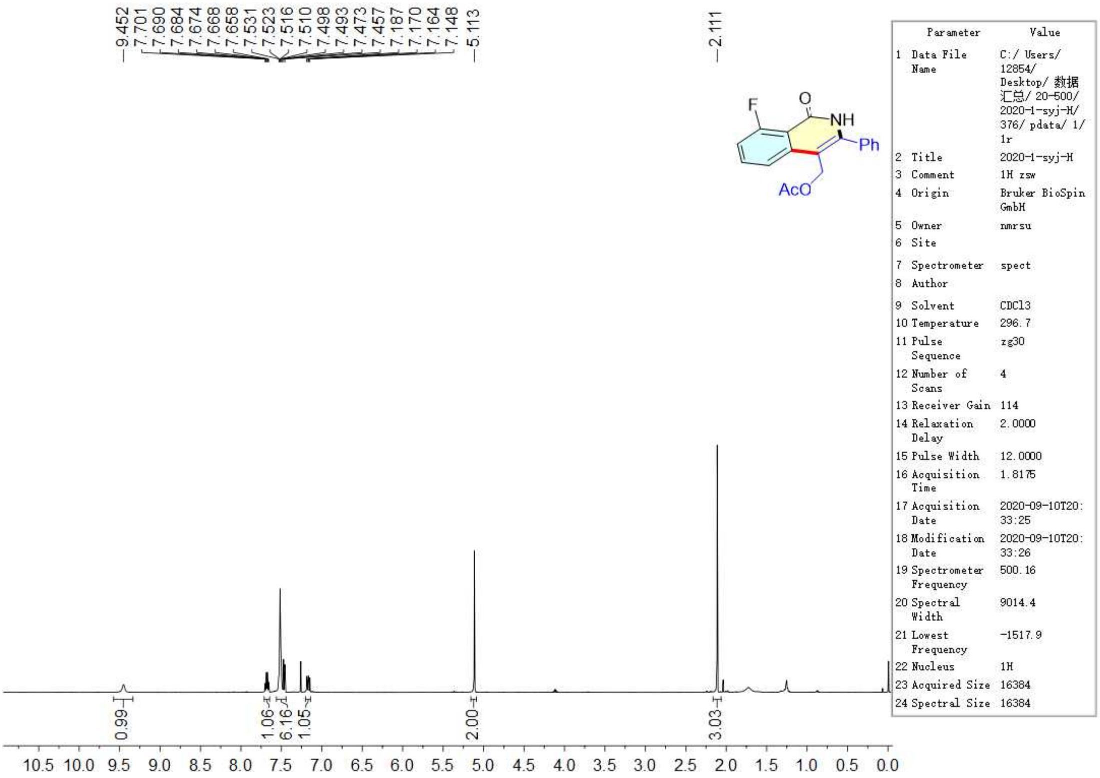

^{1}H NMR spectrum for compound **3pa**

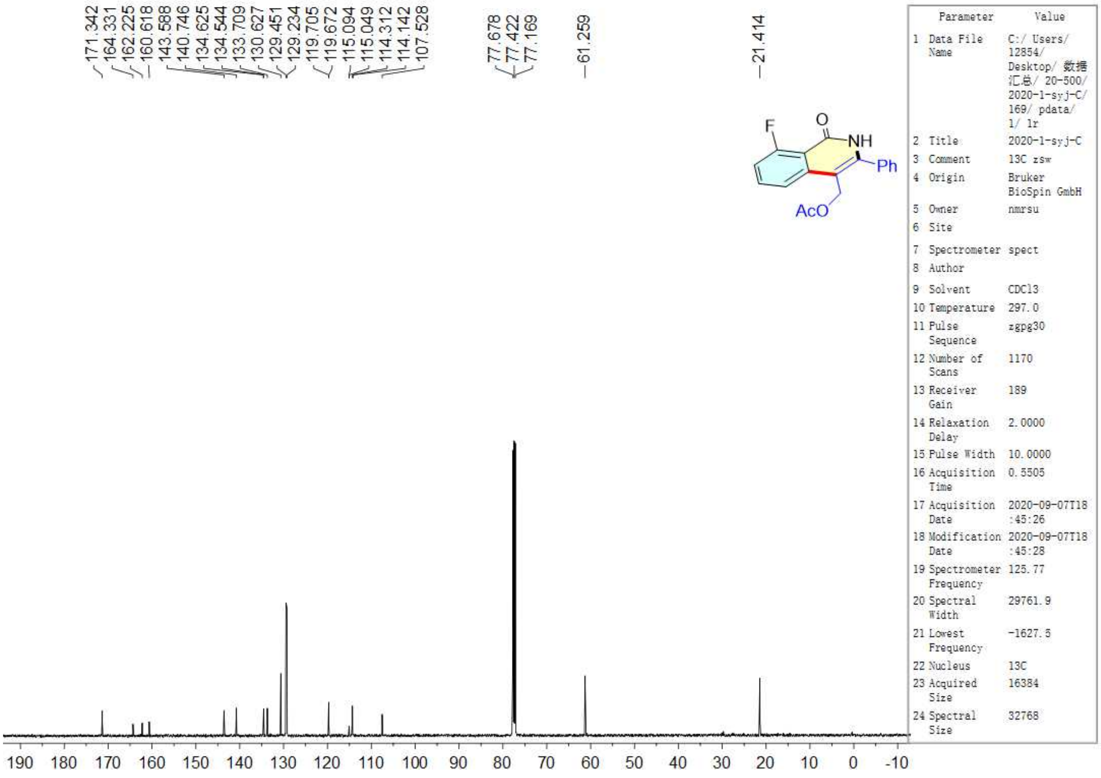

^{13}C NMR spectrum for compound **3pa**

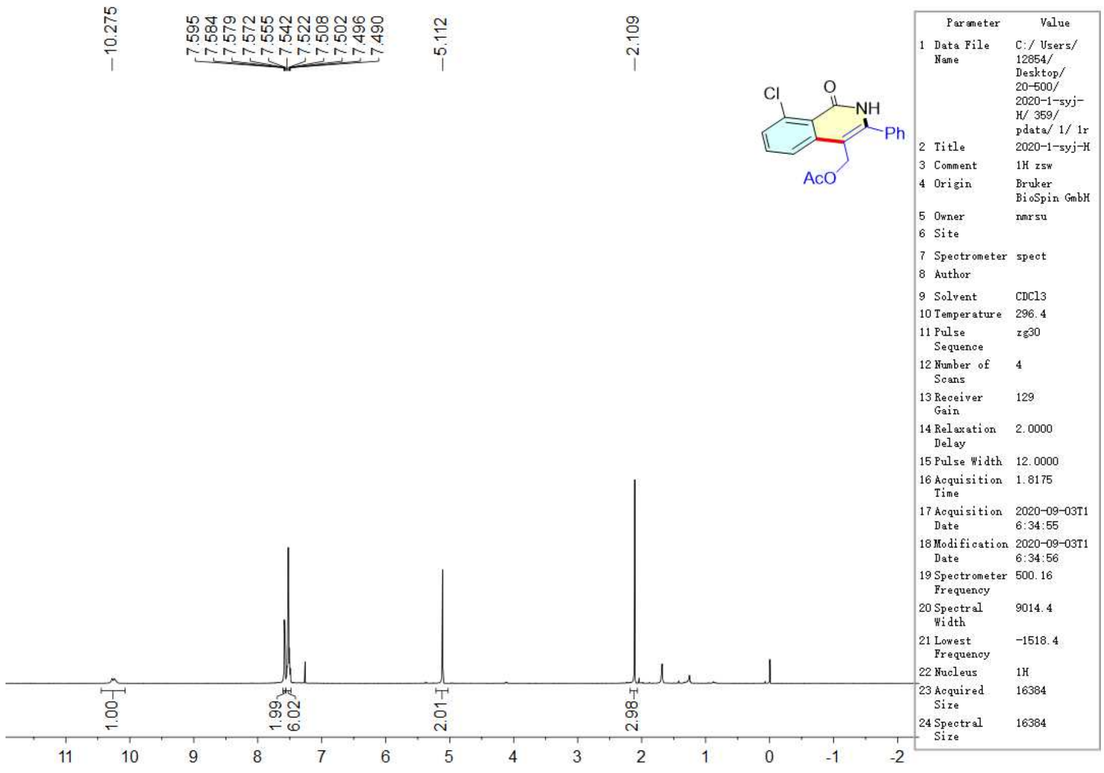

^{1}H NMR spectrum for compound **3qa**

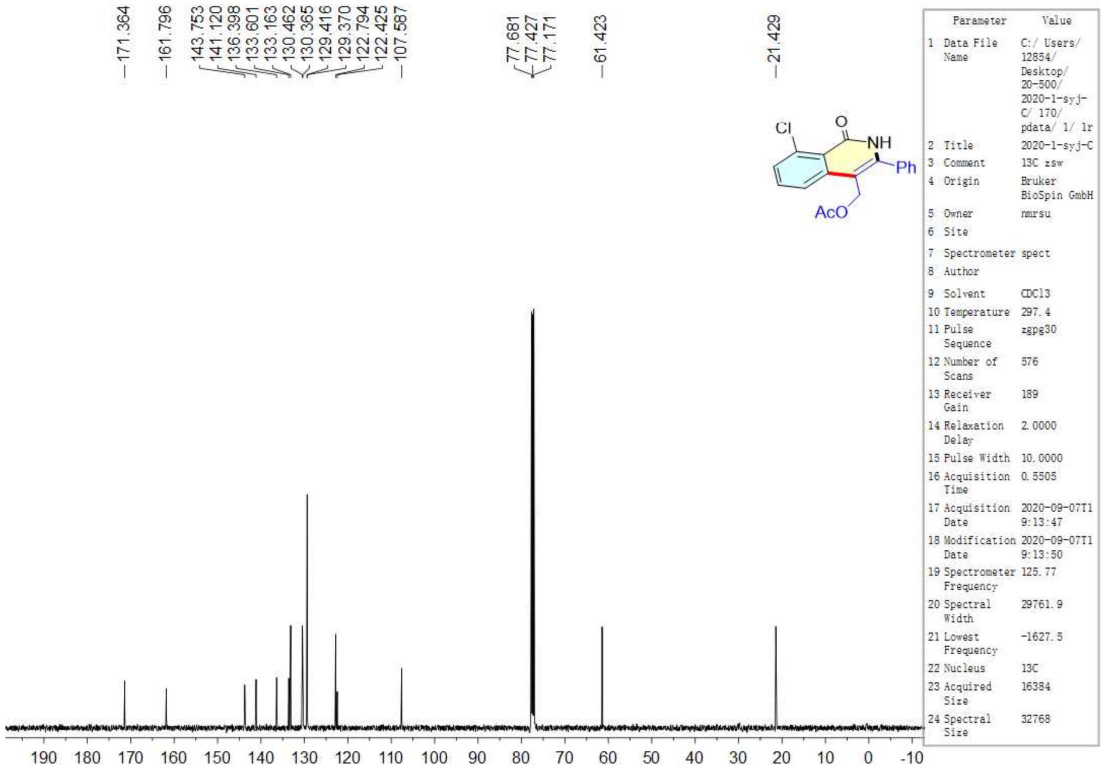

^{13}C NMR spectrum for compound **3qa**

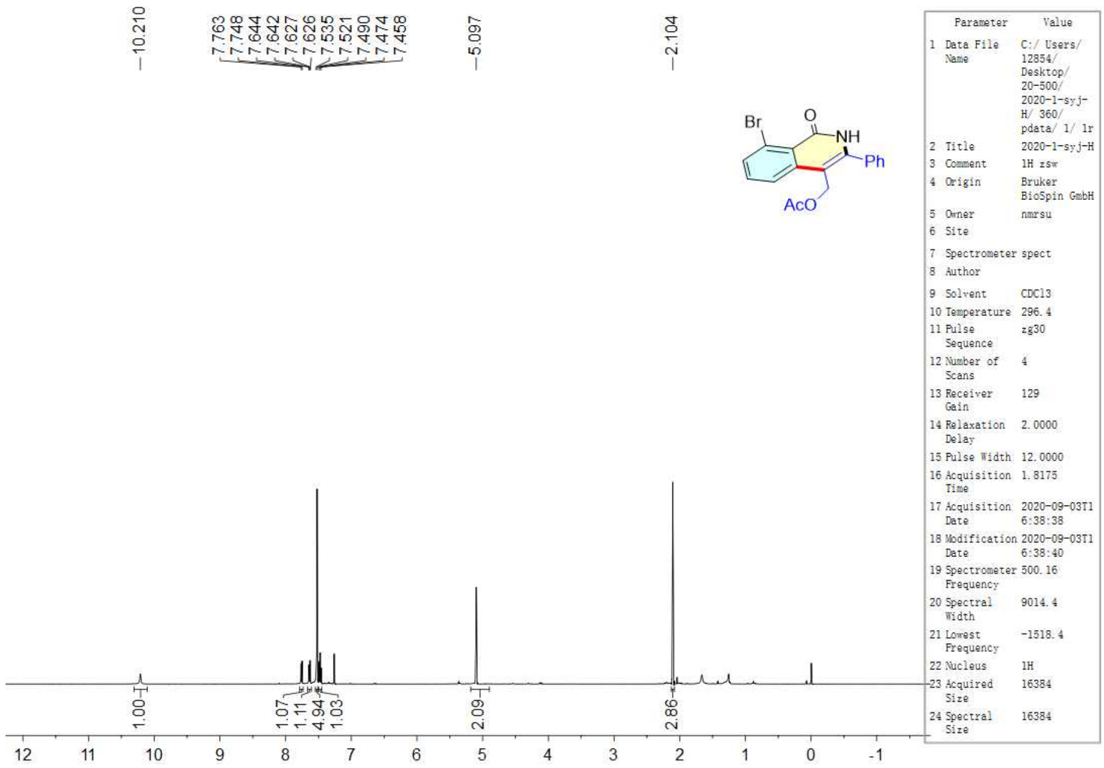

^{1}H NMR spectrum for compound **3ra**

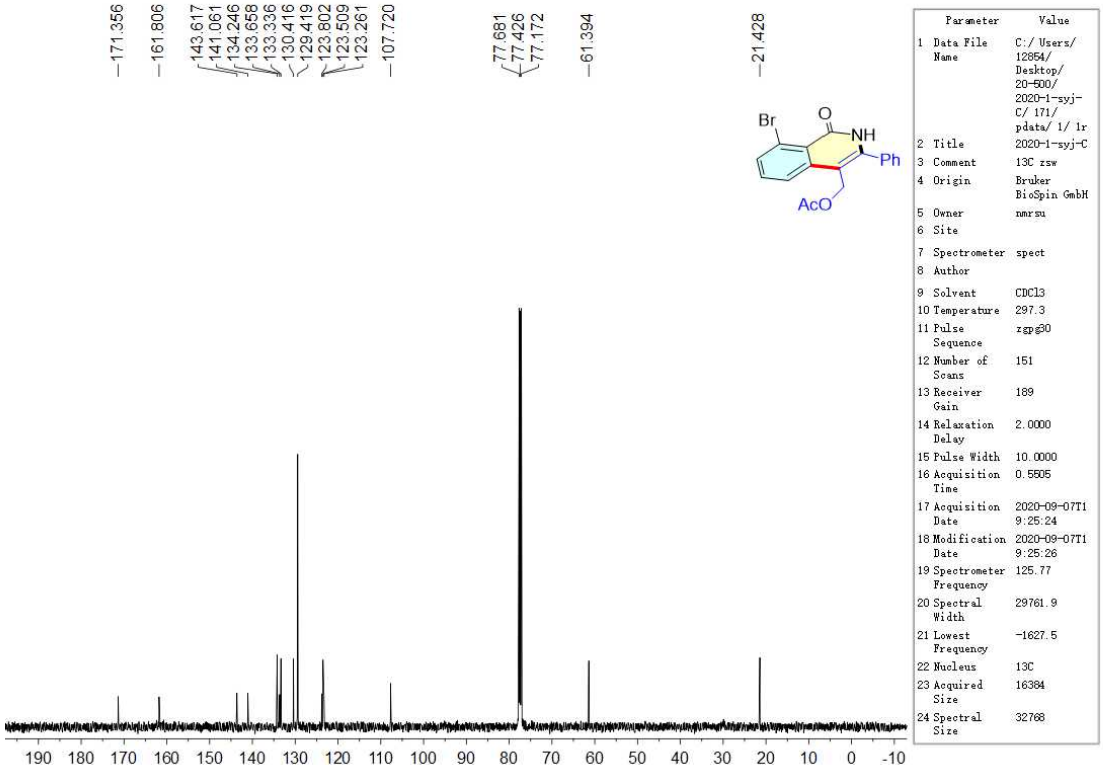

^{13}C NMR spectrum for compound **3ra**

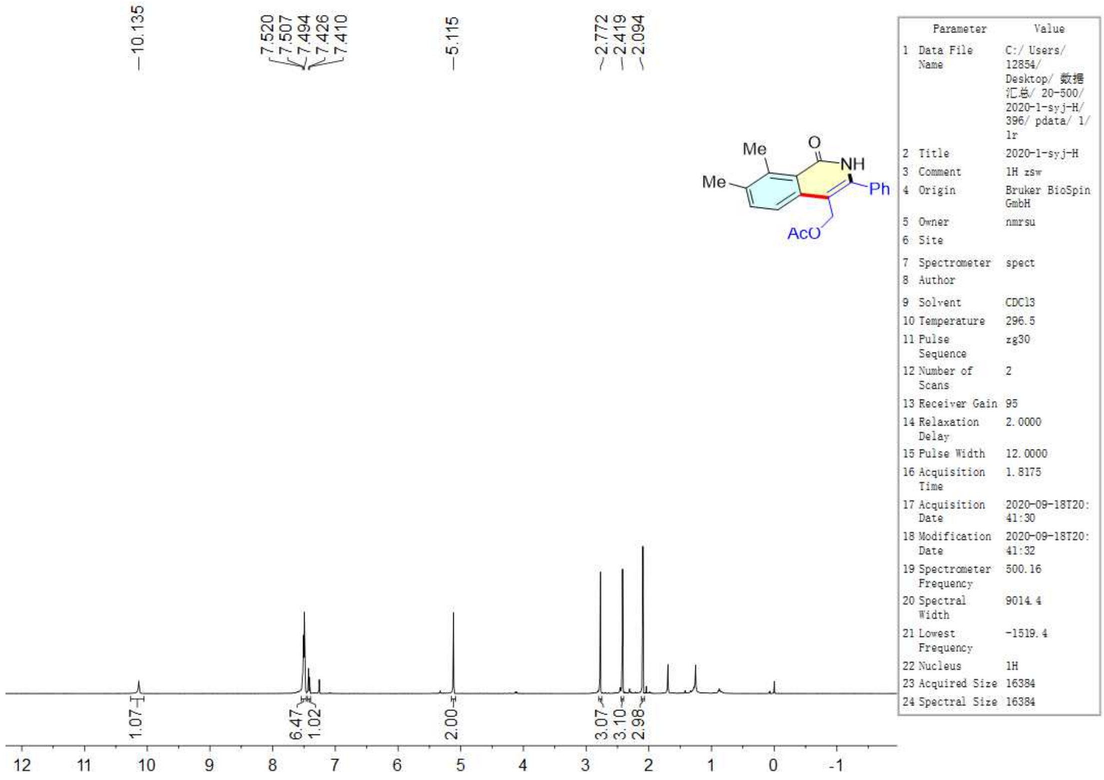

^{1}H NMR spectrum for compound **3sa**

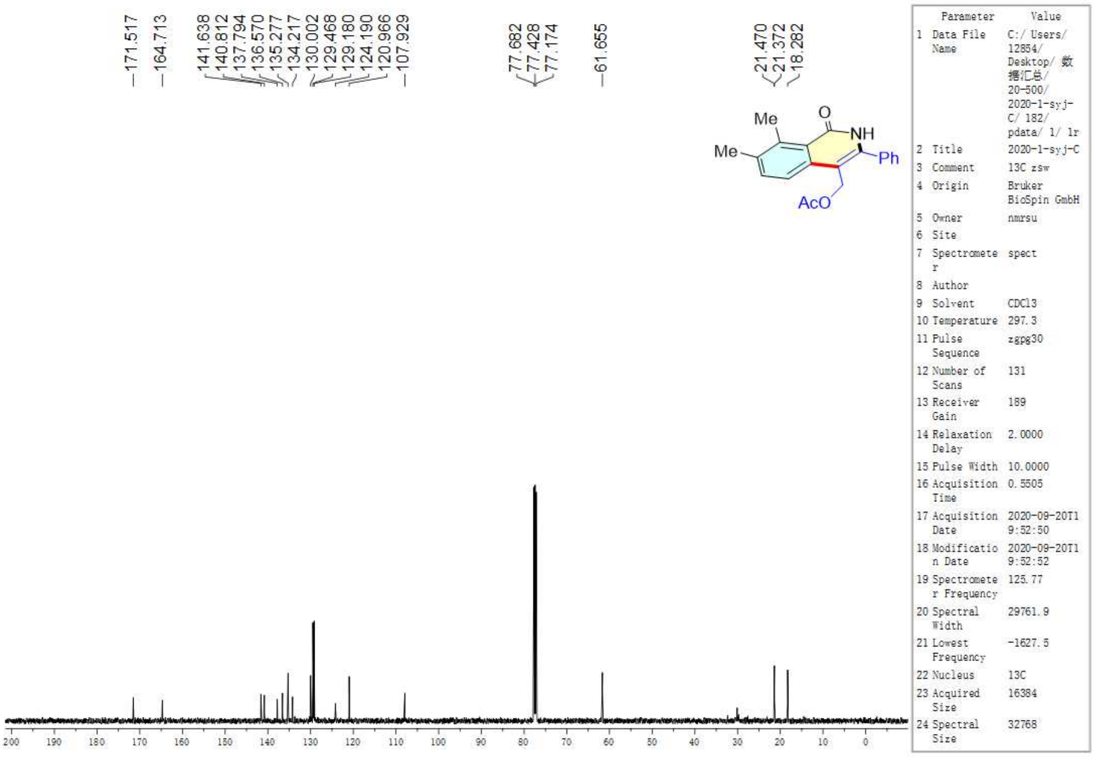

^{13}C NMR spectrum for compound **3sa**

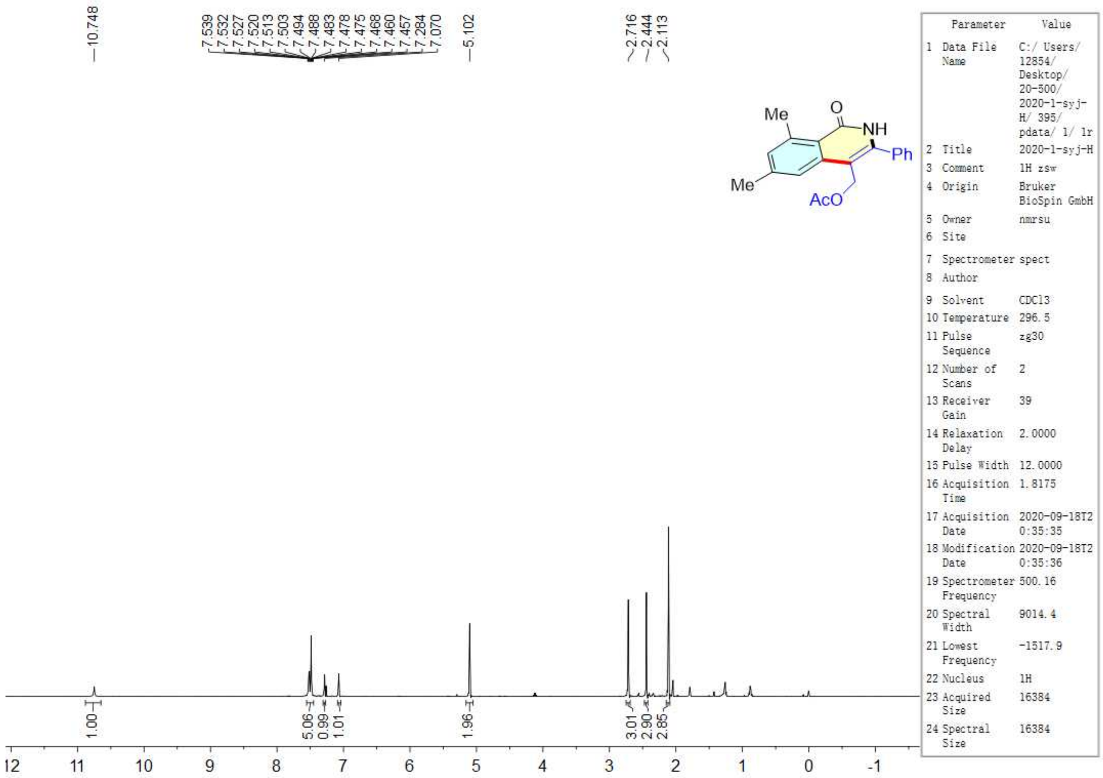

^{1}H NMR spectrum for compound **3ta**

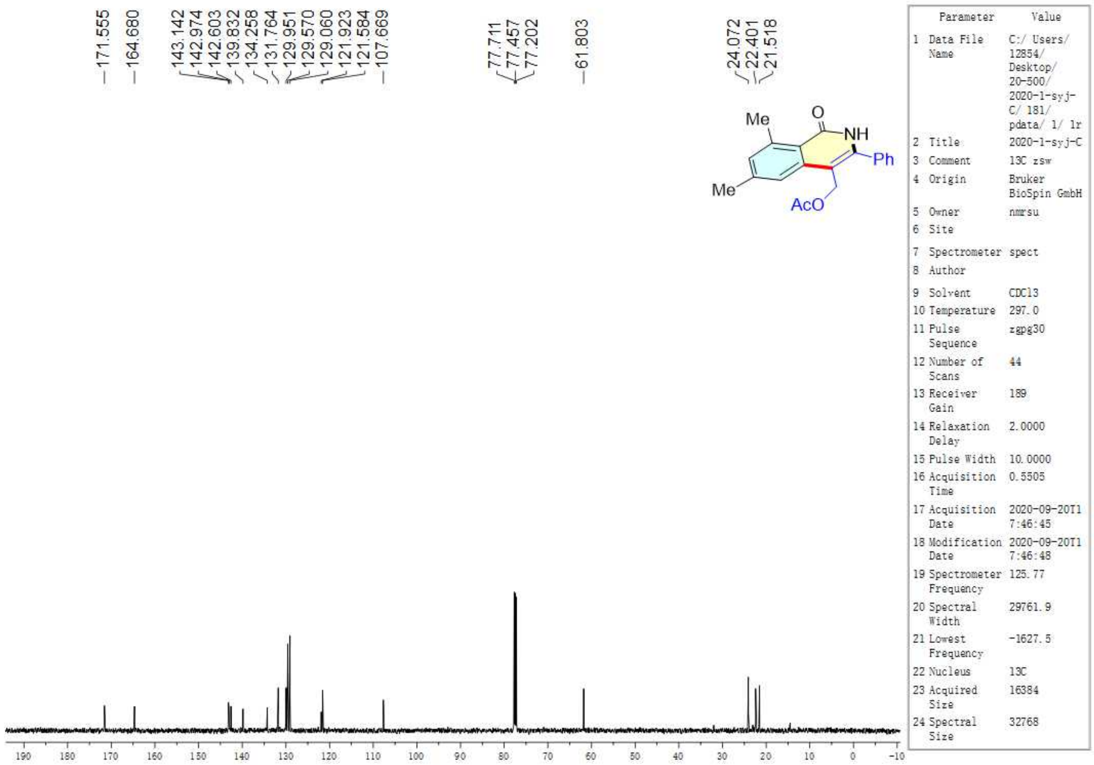

^{13}C NMR spectrum for compound **3ta**

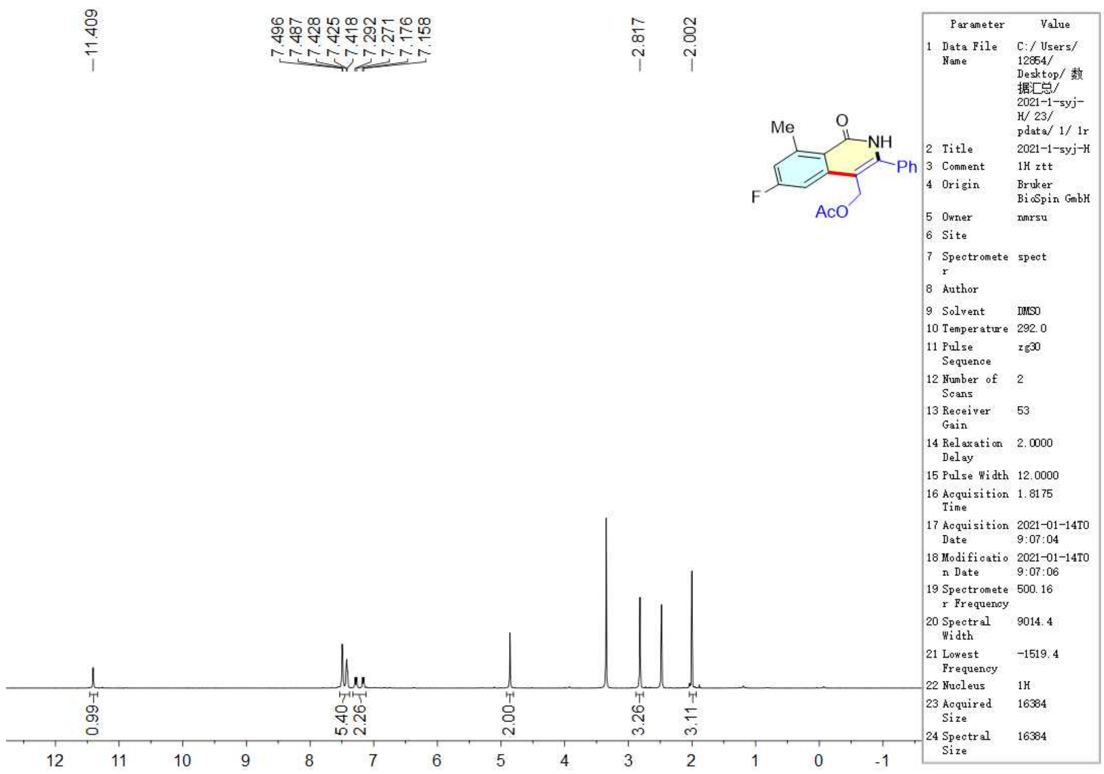

Parameter	Value
1 Data File Name	C:/ Users/ 12854/ Desktop/ 数据汇总/ 2021-1-syj-H/ 23/ pdata/ 1/ 1r
2 Title	2021-1-syj-H
3 Comment	1H ztt
4 Origin	Bruker BioSpin GmbH
5 Owner	nmrsu
6 Site	
7 Spectrometer	spect
8 Author	
9 Solvent	DMSO
10 Temperature	292.0
11 Pulse Sequence	zg30
12 Number of Scans	2
13 Receiver Gain	53
14 Relaxation Delay	2.0000
15 Pulse Width	12.0000
16 Acquisition Time	1.8175
17 Acquisition Date	2021-01-14T09:07:04
18 Modification Date	2021-01-14T09:07:06
19 Spectrometer Frequency	500.16
20 Spectral Width	9014.4
21 Lowest Frequency	-1519.4
22 Nucleus	1H
23 Acquired Size	16384
24 Spectral Size	16384

^{1}H NMR spectrum for compound **3ua**

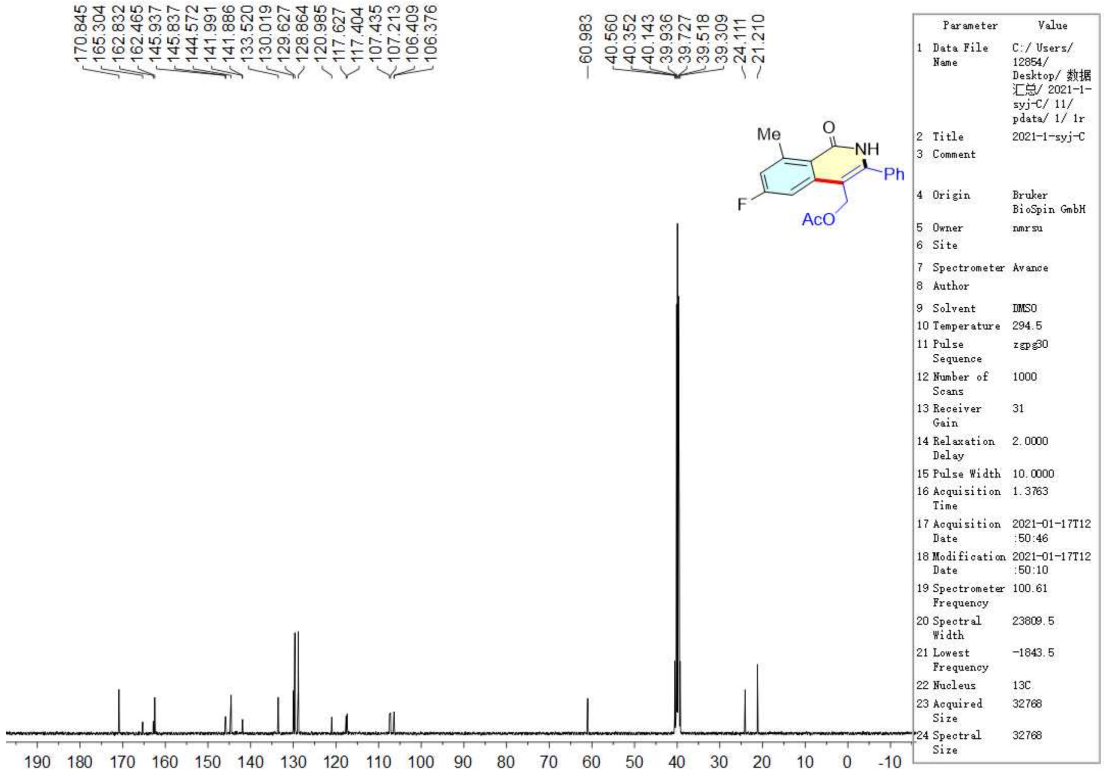

Parameter	Value
1 Data File Name	C:/ Users/ 12854/ Desktop/ 数据汇总/ 2021-1-syj-C/ 11/ pdata/ 1/ 1r
2 Title	2021-1-syj-C
3 Comment	
4 Origin	Bruker BioSpin GmbH
5 Owner	nmrsu
6 Site	
7 Spectrometer	Avance
8 Author	
9 Solvent	DMSO
10 Temperature	294.5
11 Pulse Sequence	zgpg30
12 Number of Scans	1000
13 Receiver Gain	31
14 Relaxation Delay	2.0000
15 Pulse Width	10.0000
16 Acquisition Time	1.3763
17 Acquisition Date	2021-01-17T12:50:46
18 Modification Date	2021-01-17T12:50:10
19 Spectrometer Frequency	100.61
20 Spectral Width	23809.5
21 Lowest Frequency	-1843.5
22 Nucleus	13C
23 Acquired Size	32768
24 Spectral Size	32768

^{13}C NMR spectrum for compound **3ua**

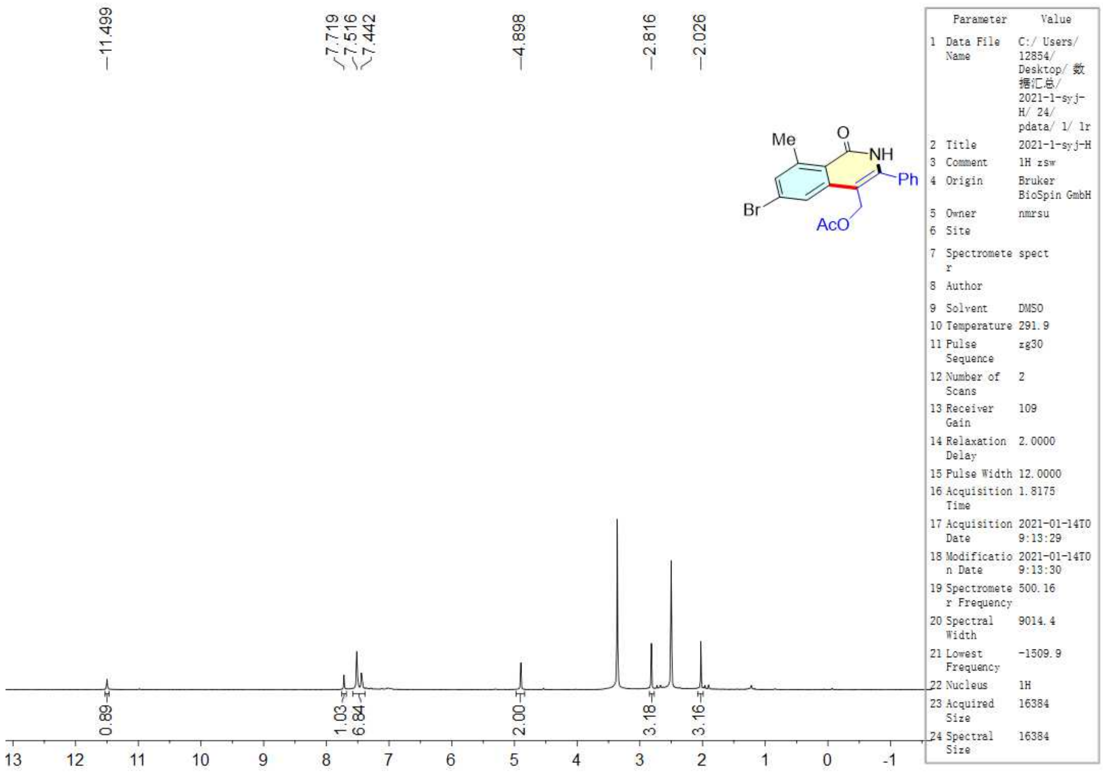

^{1}H NMR spectrum for compound **3va**

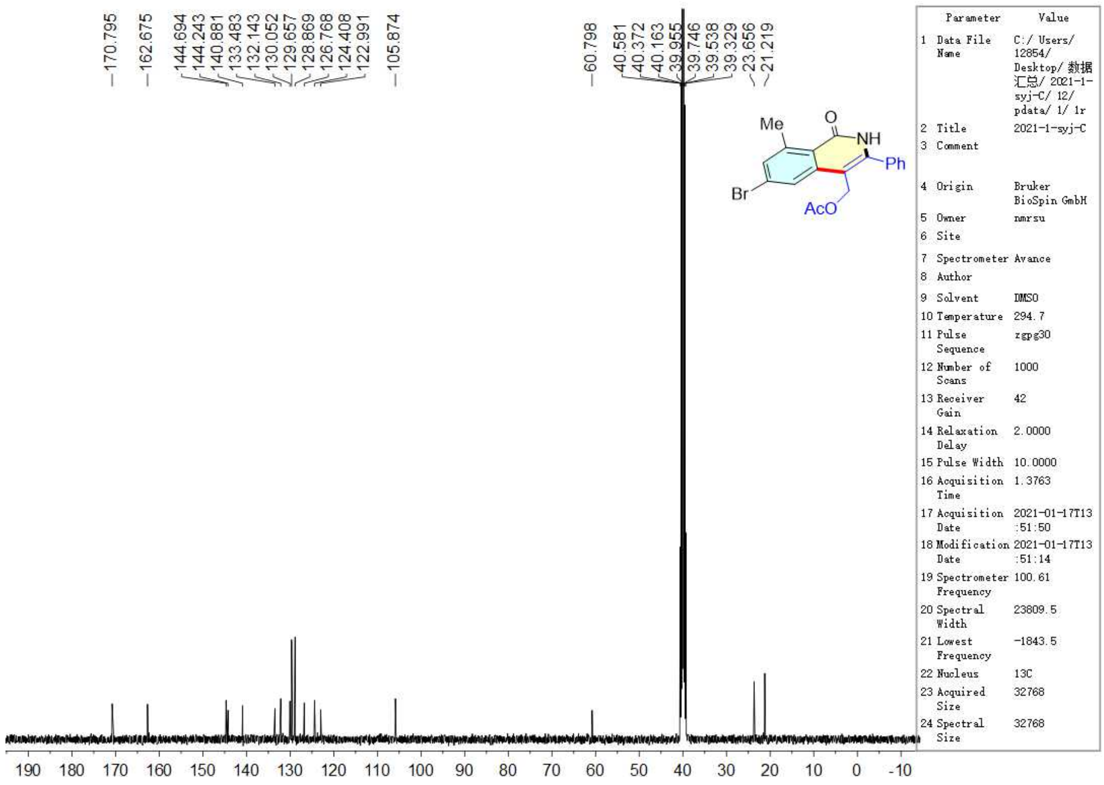

^{13}C NMR spectrum for compound **3va**

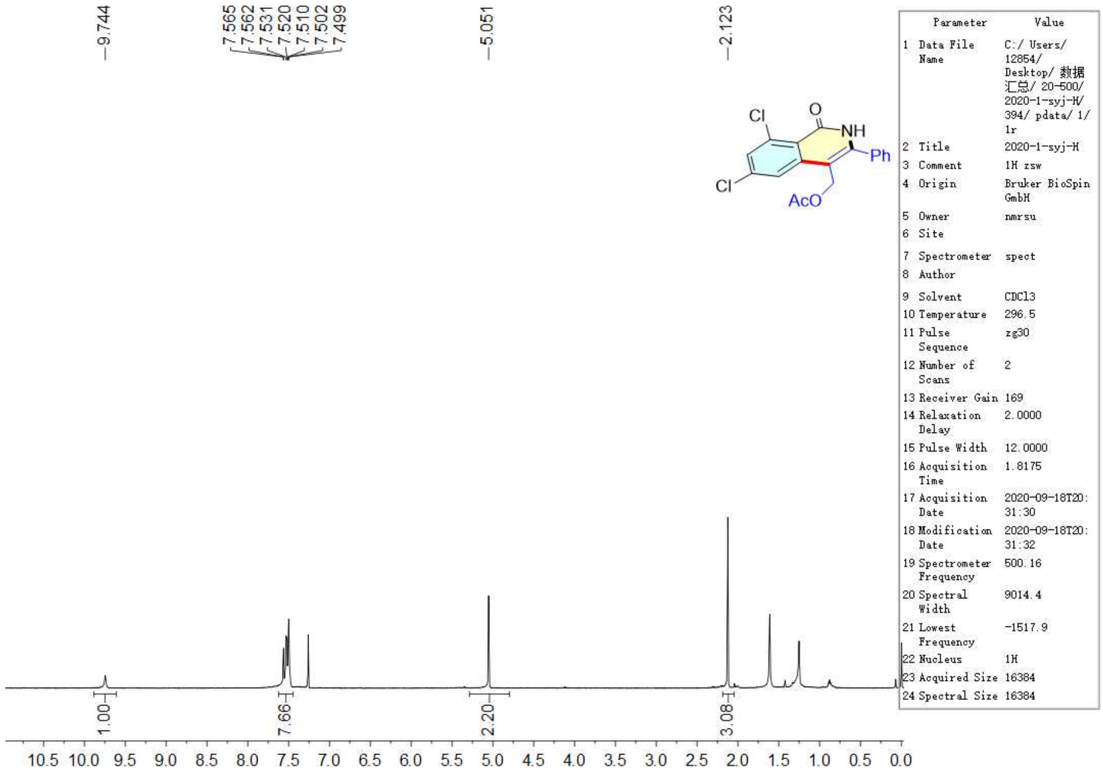

^{1}H NMR spectrum for compound **3wa**

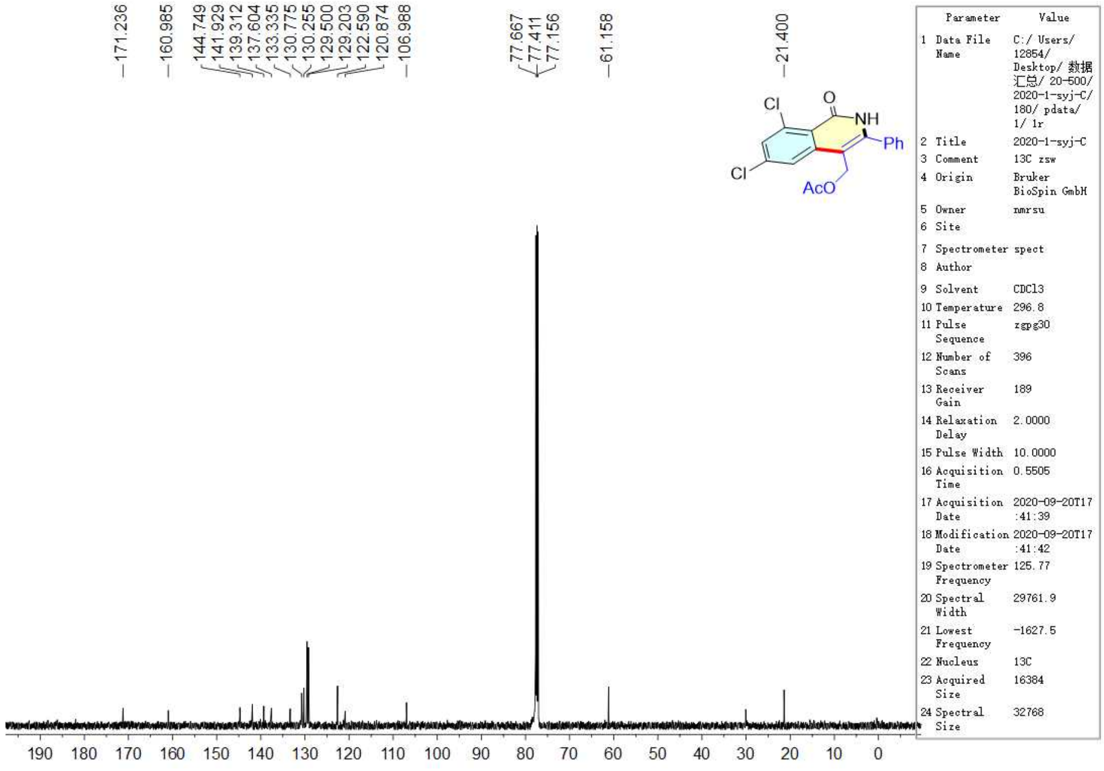

^{13}C NMR spectrum for compound **3wa**

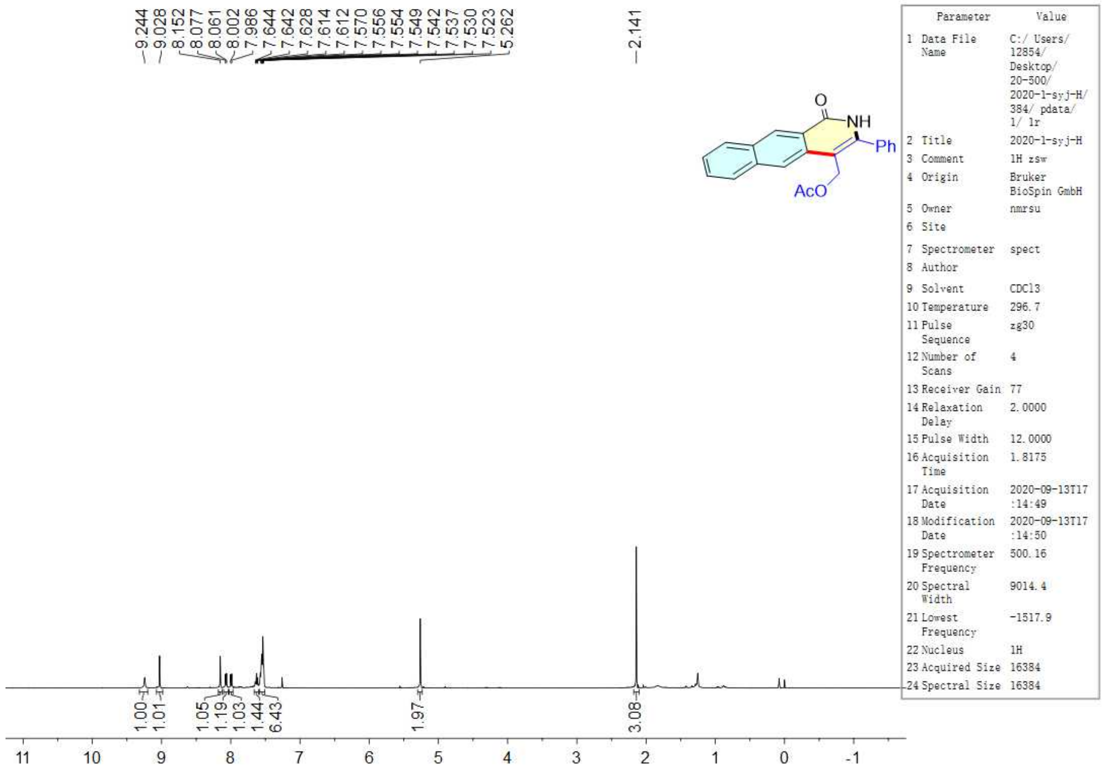

^{1}H NMR spectrum for compound **3xa**

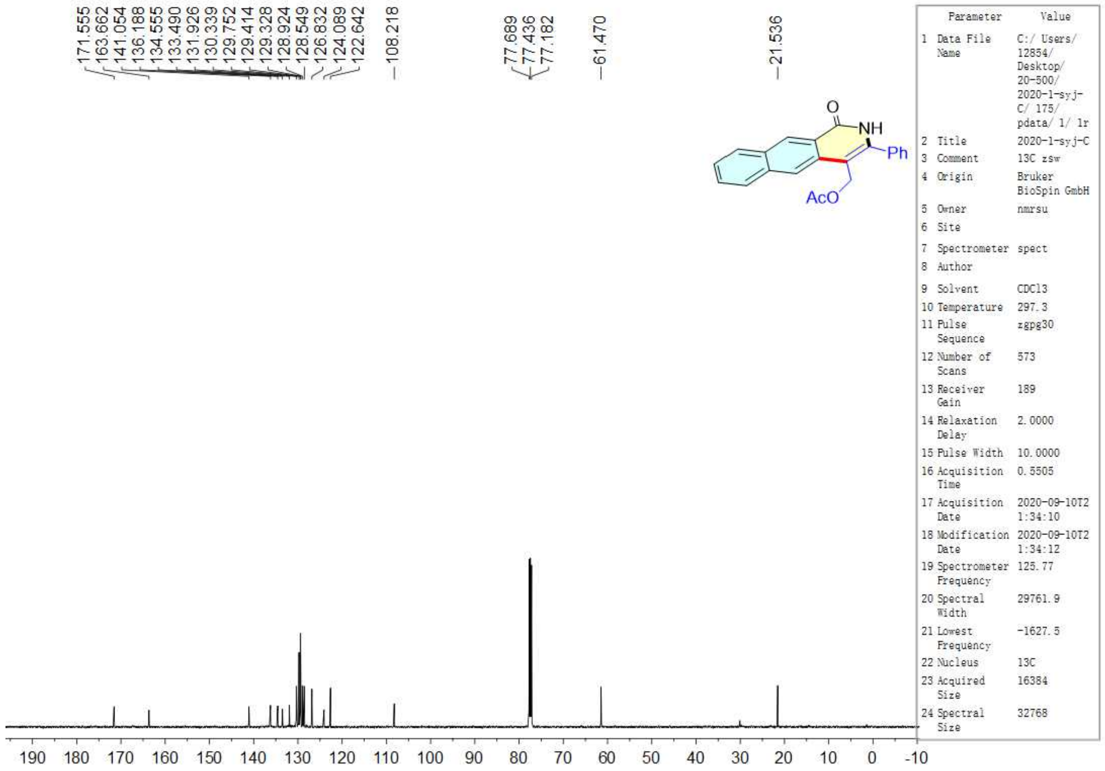

^{13}C NMR spectrum for compound **3xa**

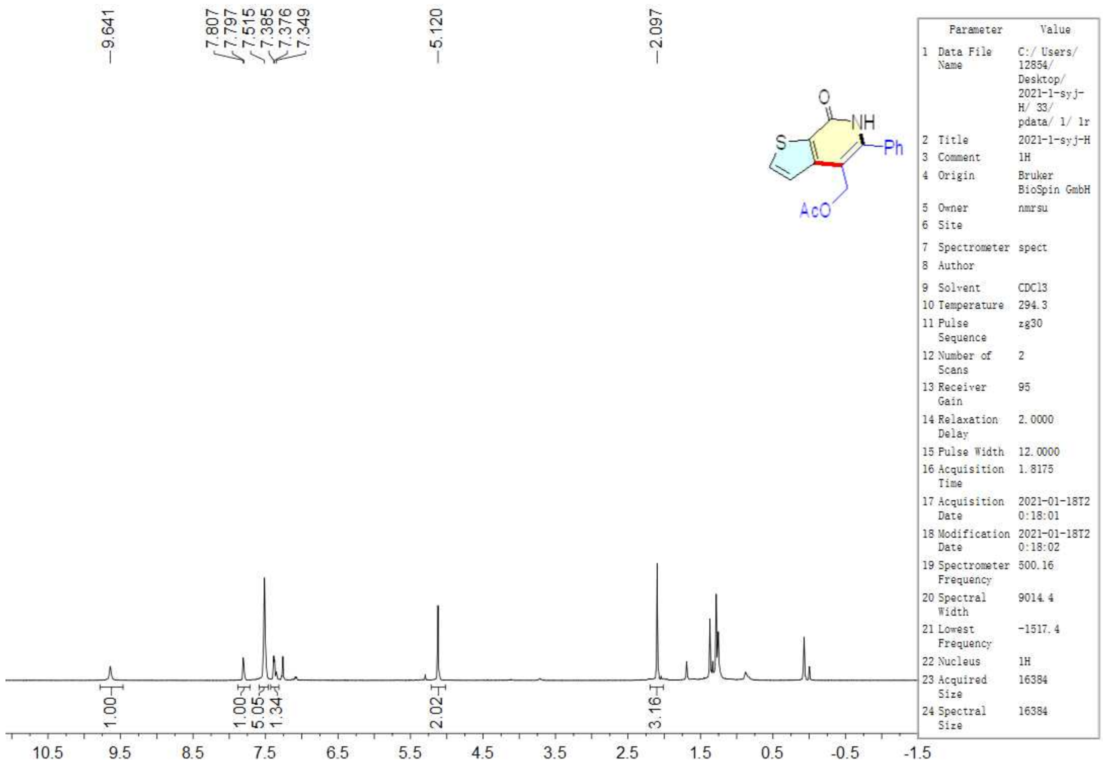

^{1}H NMR spectrum for compound **3ya**

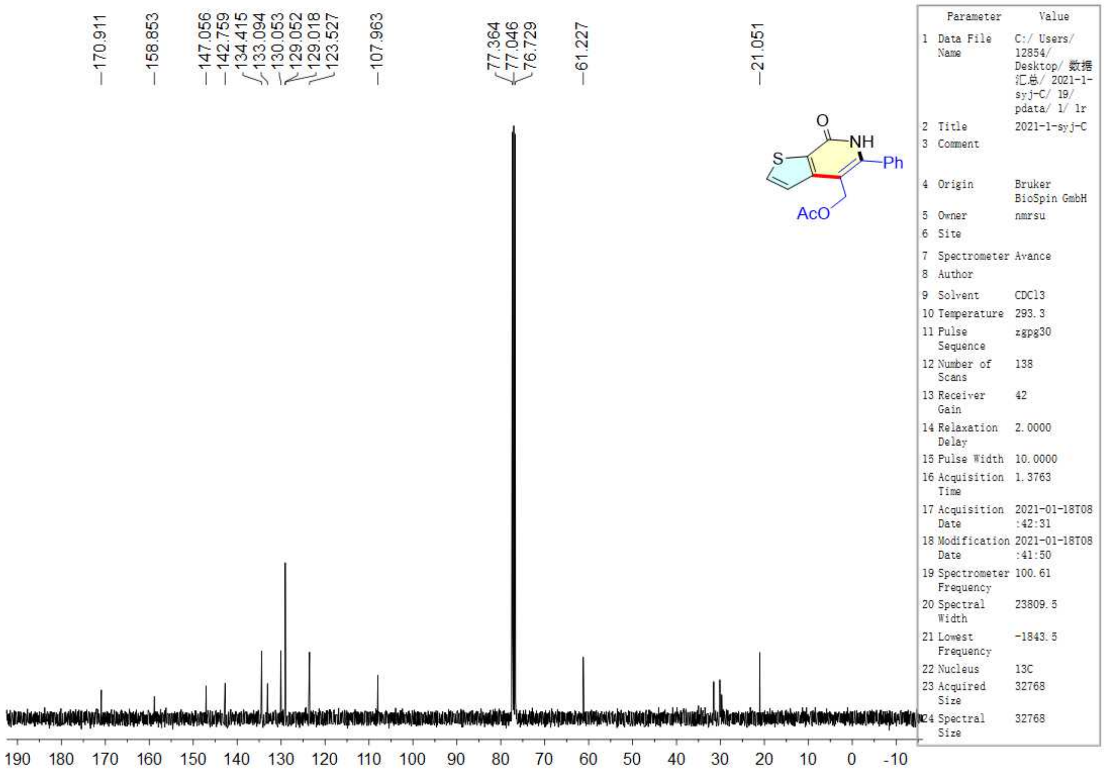

^{13}C NMR spectrum for compound **3ya**

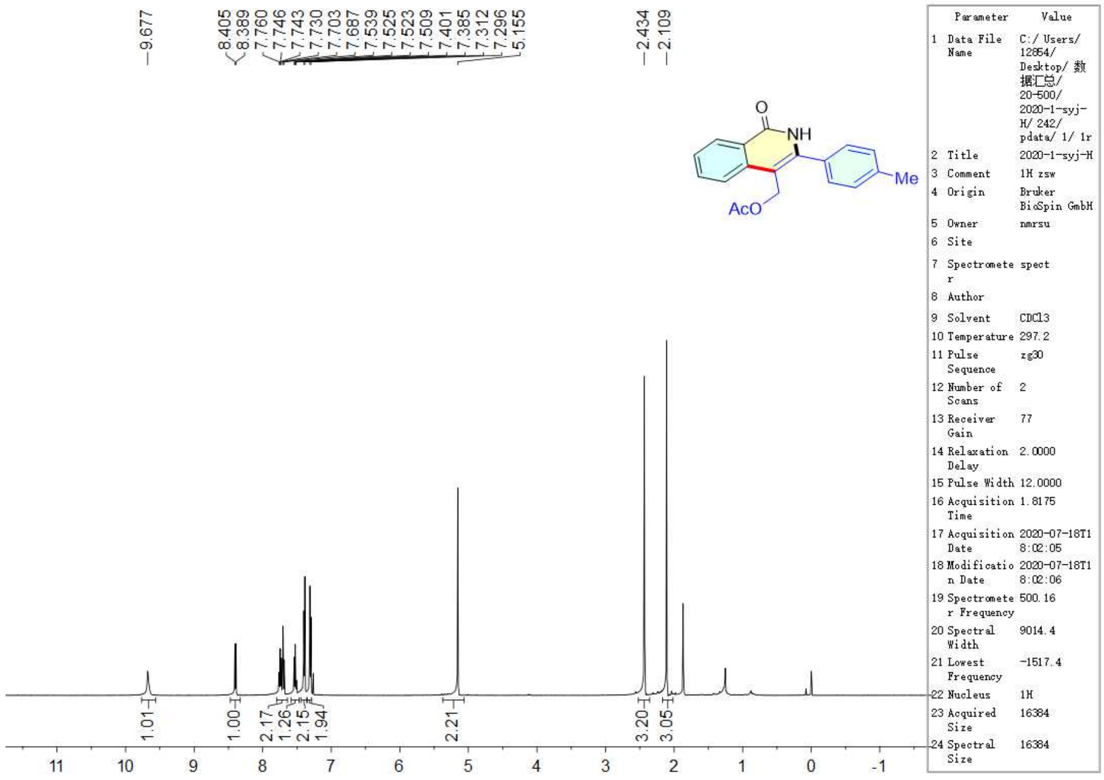

^{1}H NMR spectrum for compound **3ab**

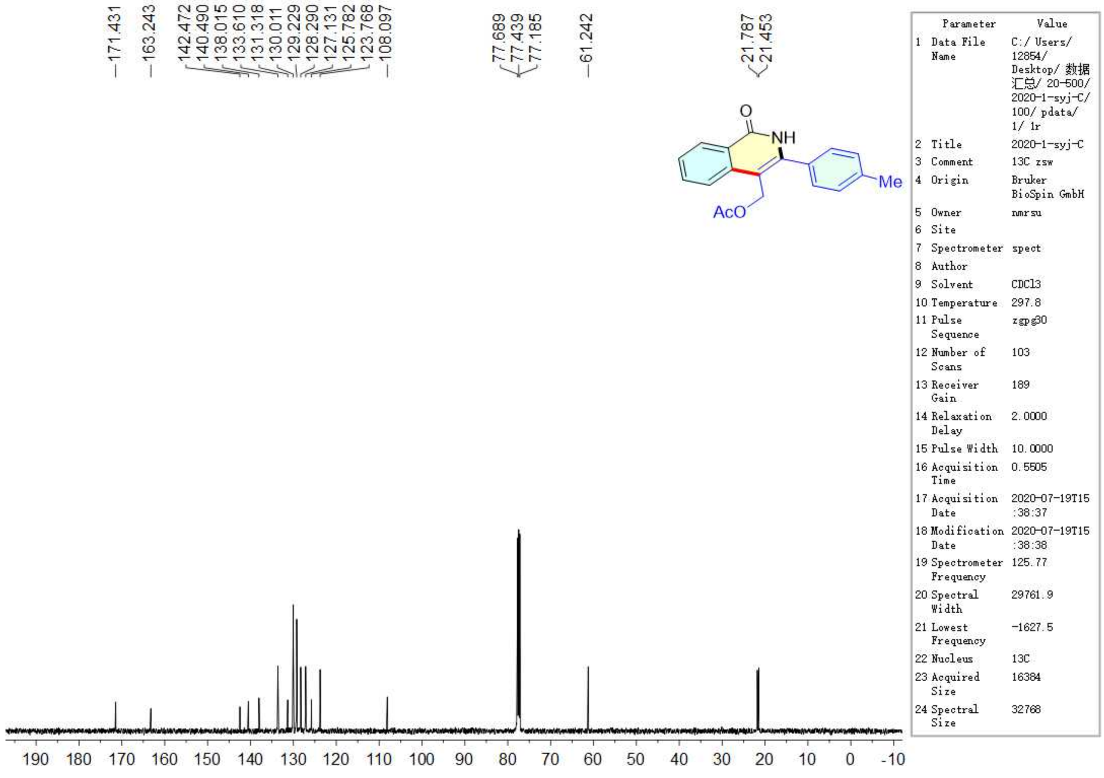

^{13}C NMR spectrum for compound **3ab**

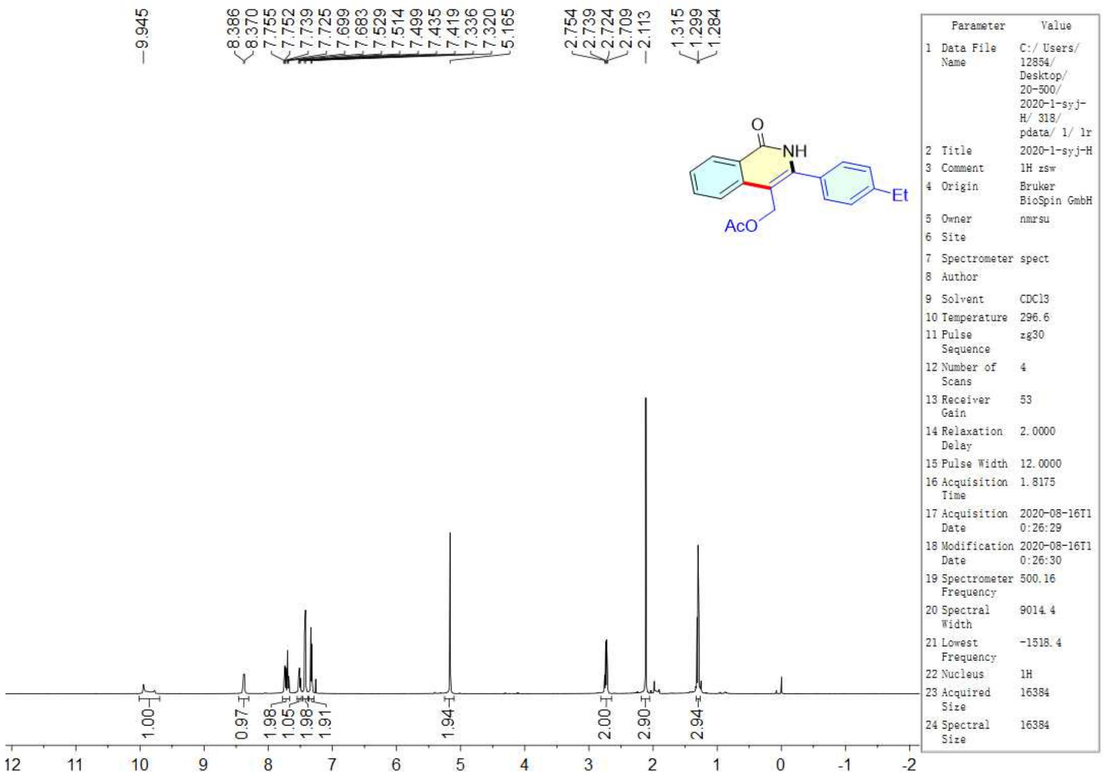

^{1}H NMR spectrum for compound **3ac**

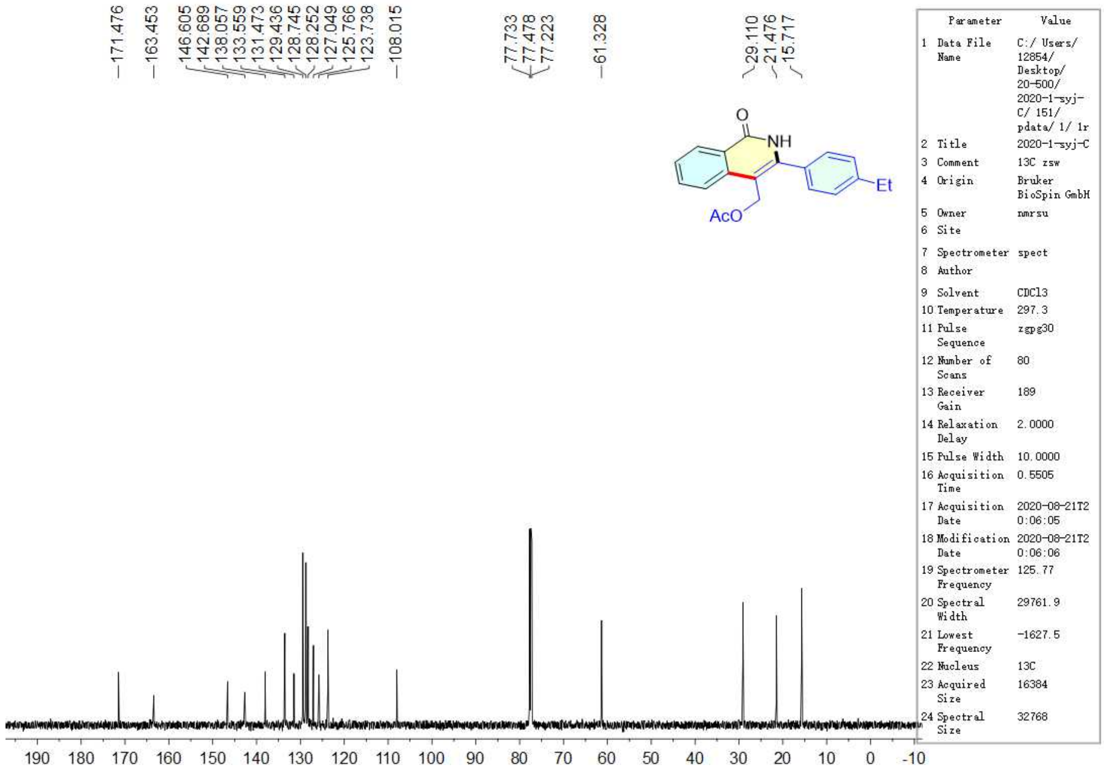

^{13}C NMR spectrum for compound **3ac**

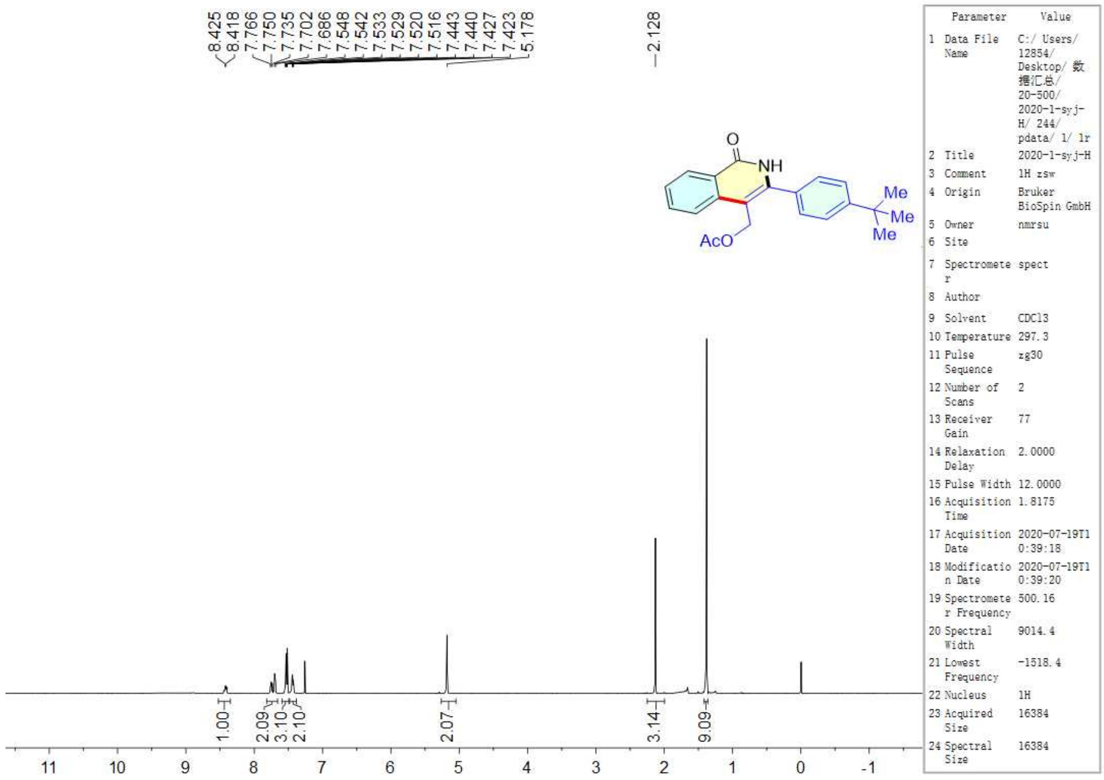

^{1}H NMR spectrum for compound **3ad**

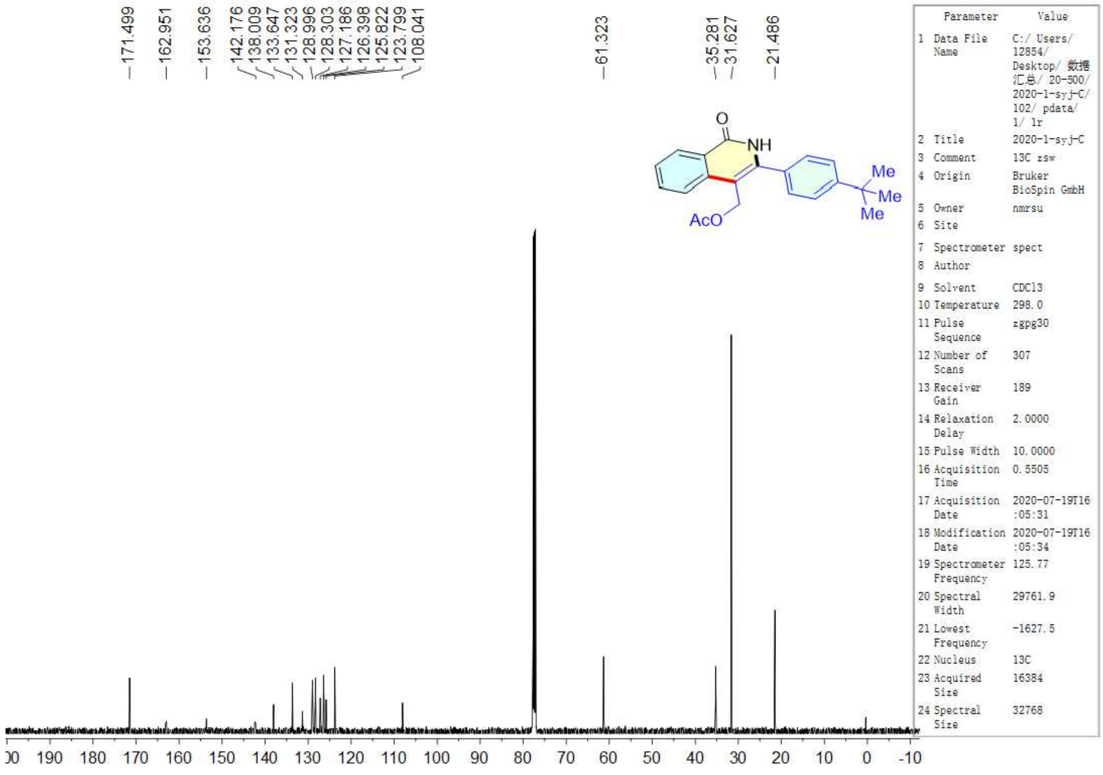

^{13}C NMR spectrum for compound **3ad**

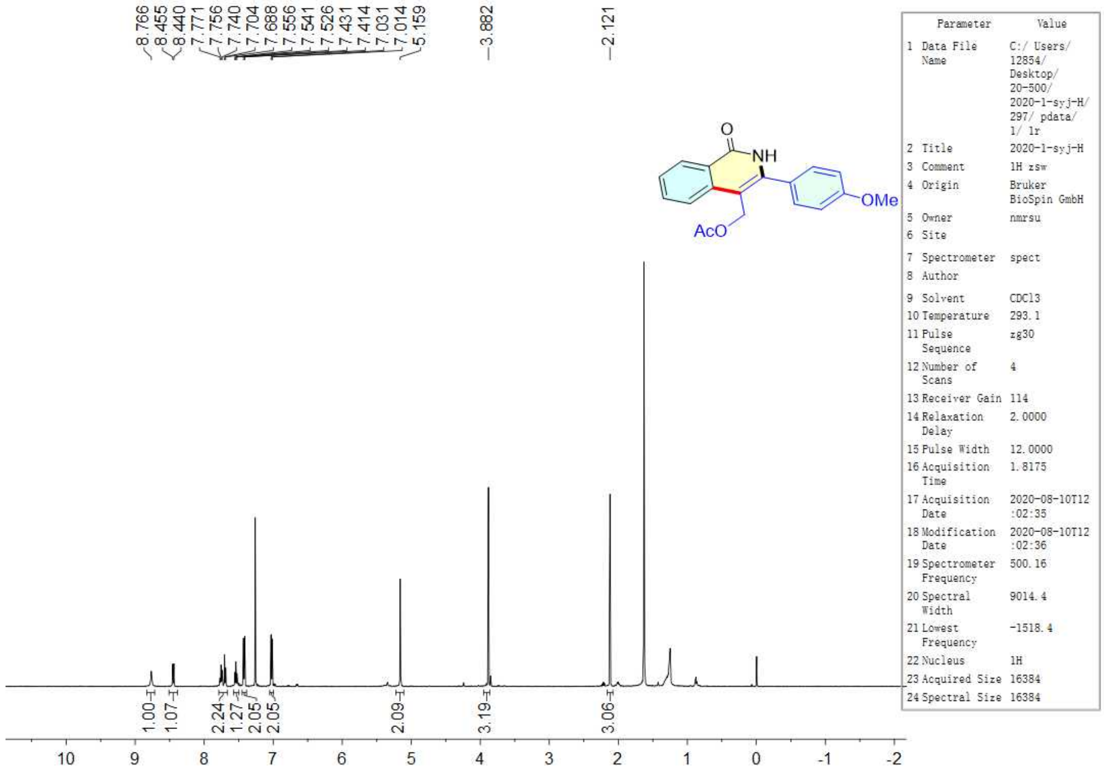

^{1}H NMR spectrum for compound **3ae**

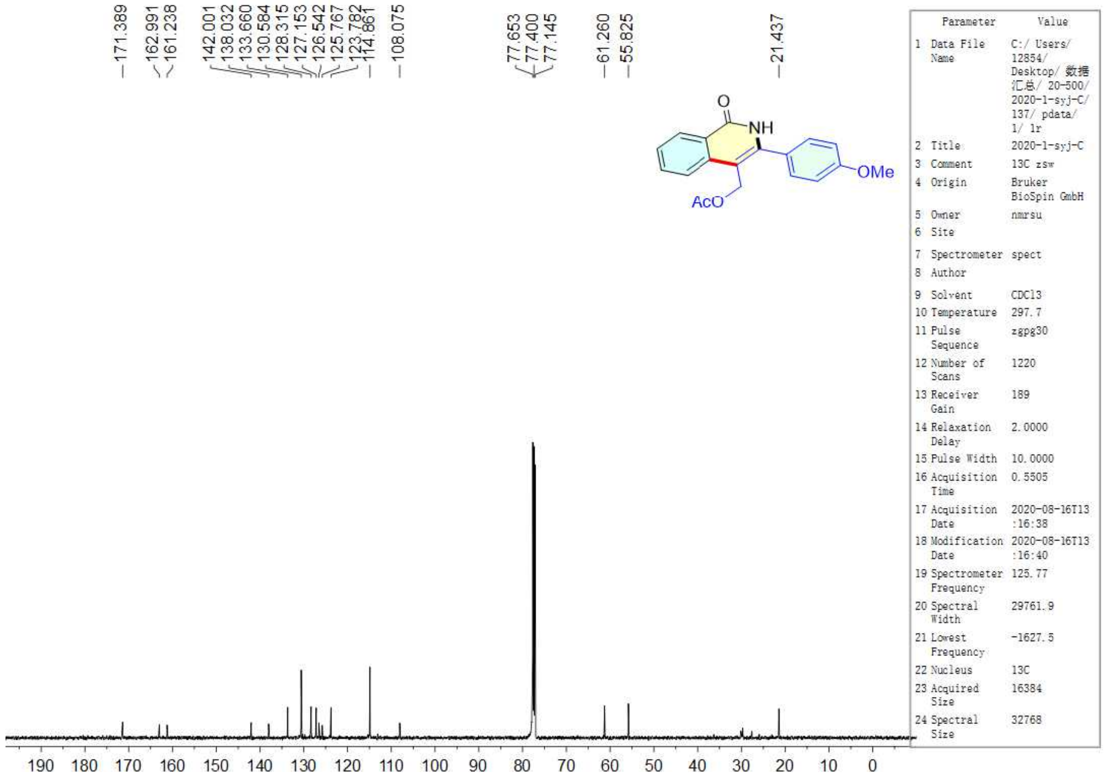

^{13}C NMR spectrum for compound **3ae**

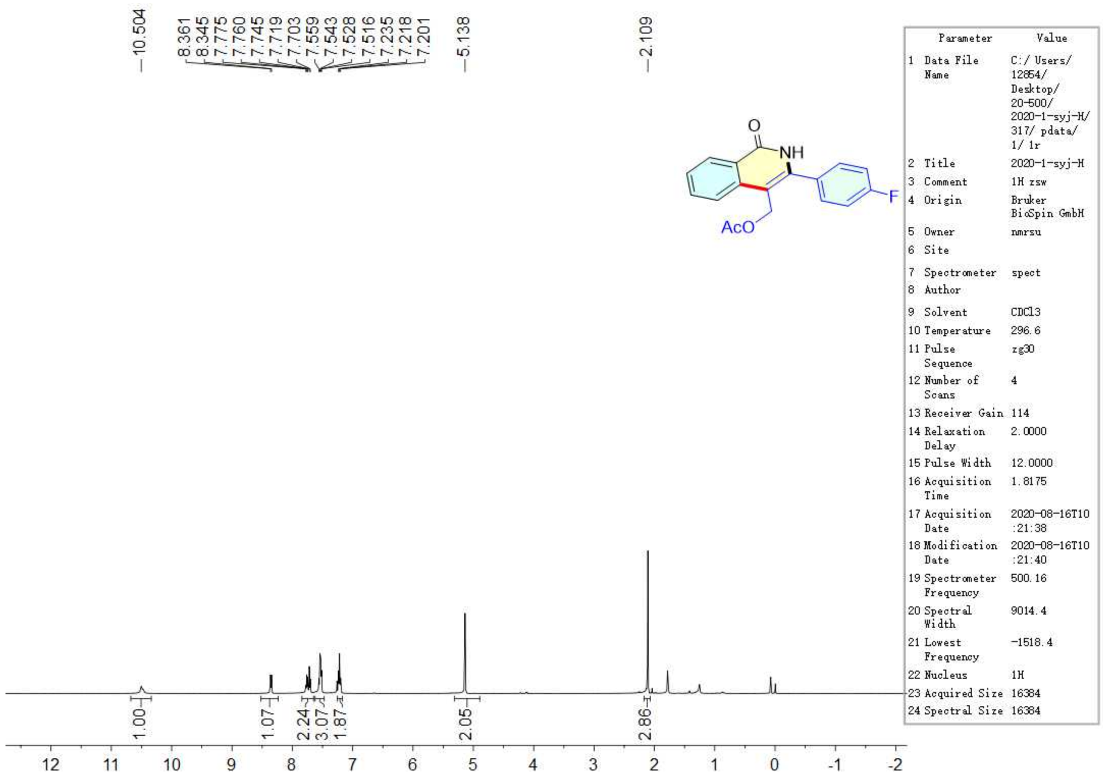

^{1}H NMR spectrum for compound **3af**

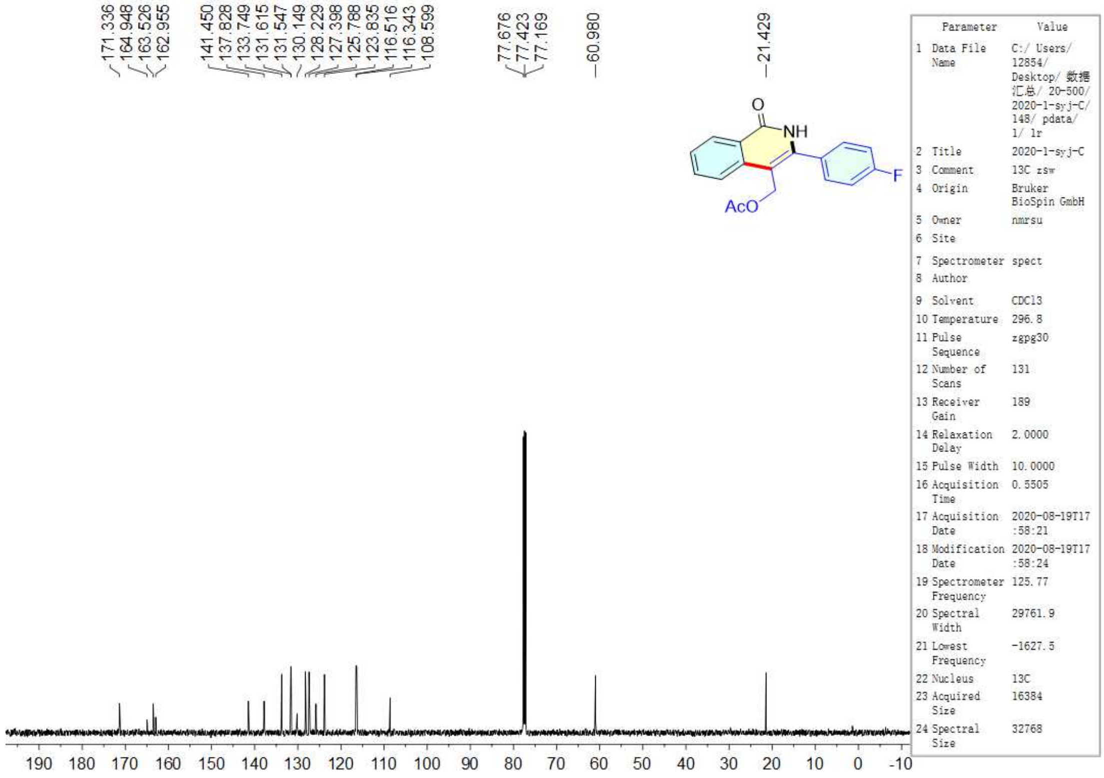

^{13}C NMR spectrum for compound **3af**

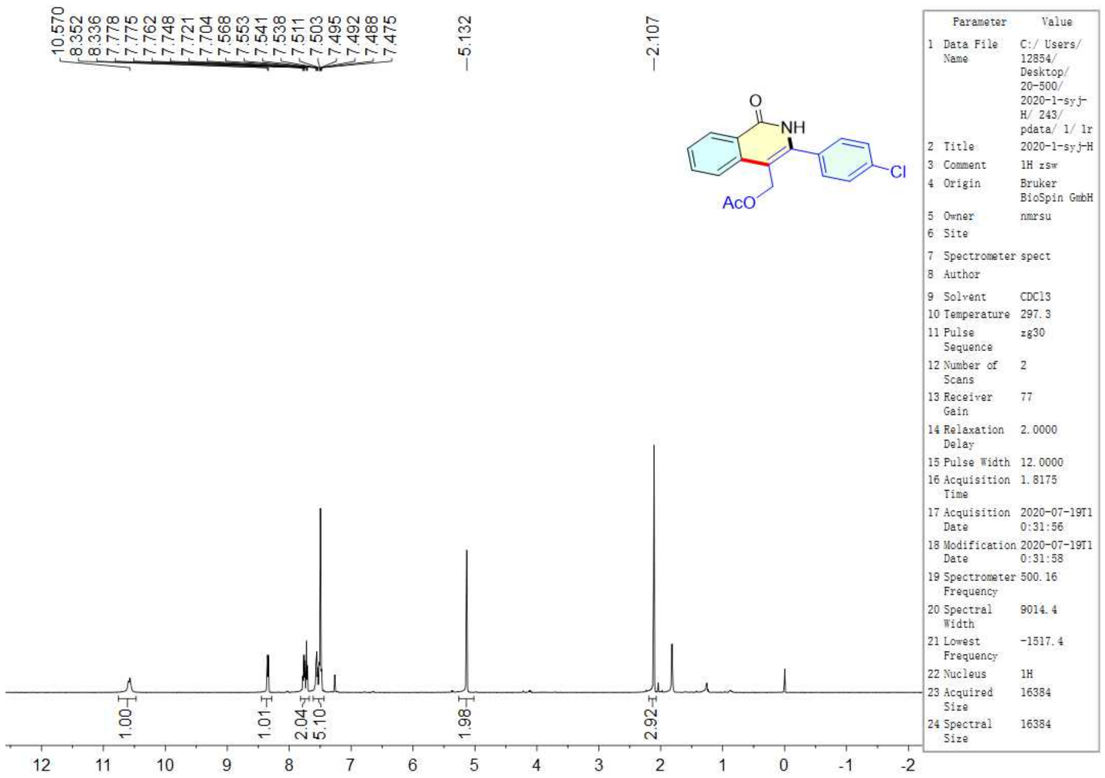

^{1}H NMR spectrum for compound **3ag**

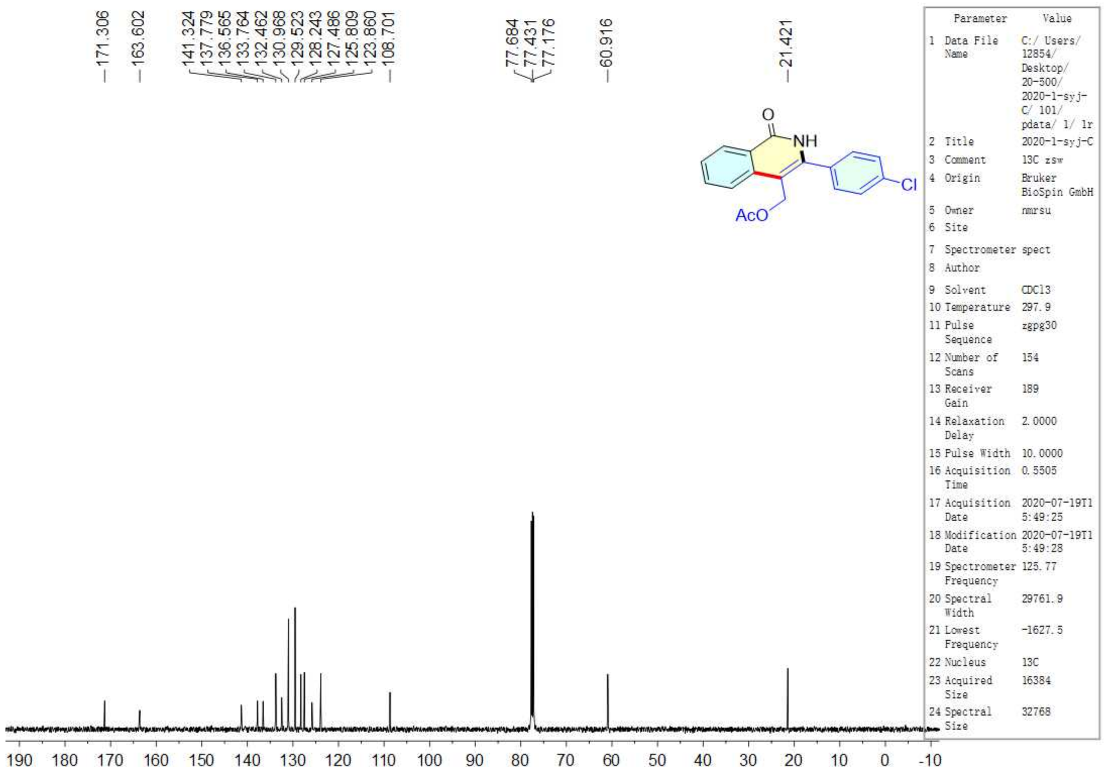

^{13}C NMR spectrum for compound **3ag**

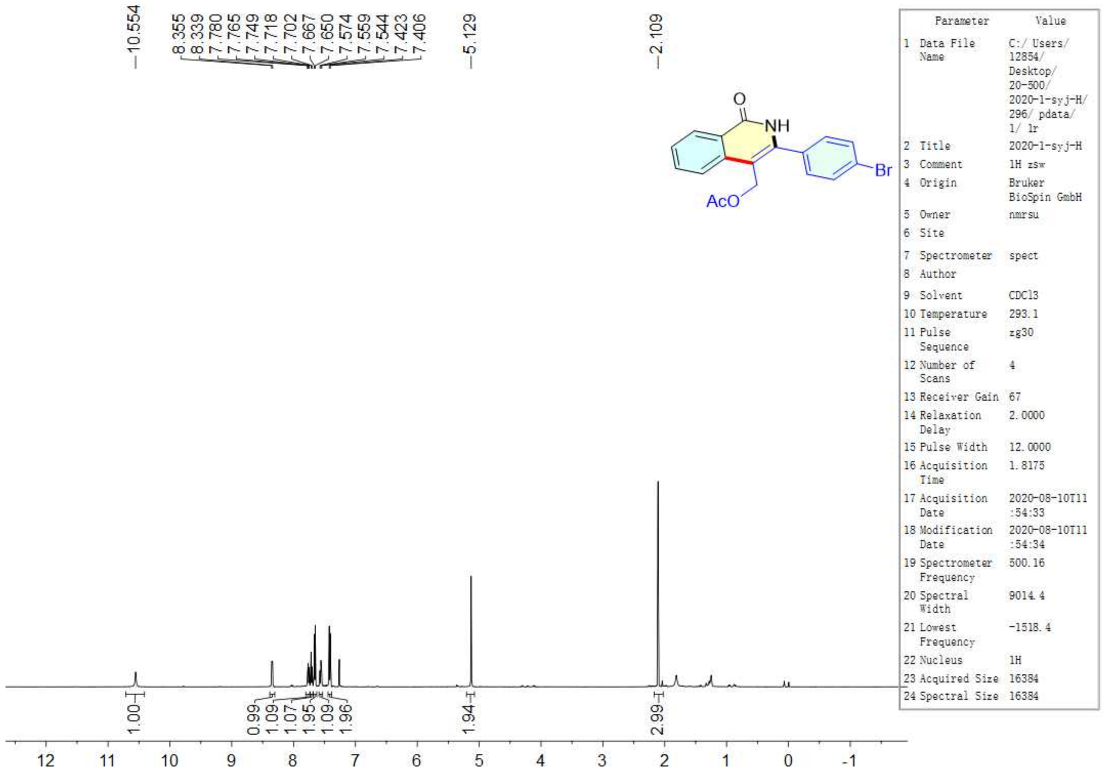

^{1}H NMR spectrum for compound **3ah**

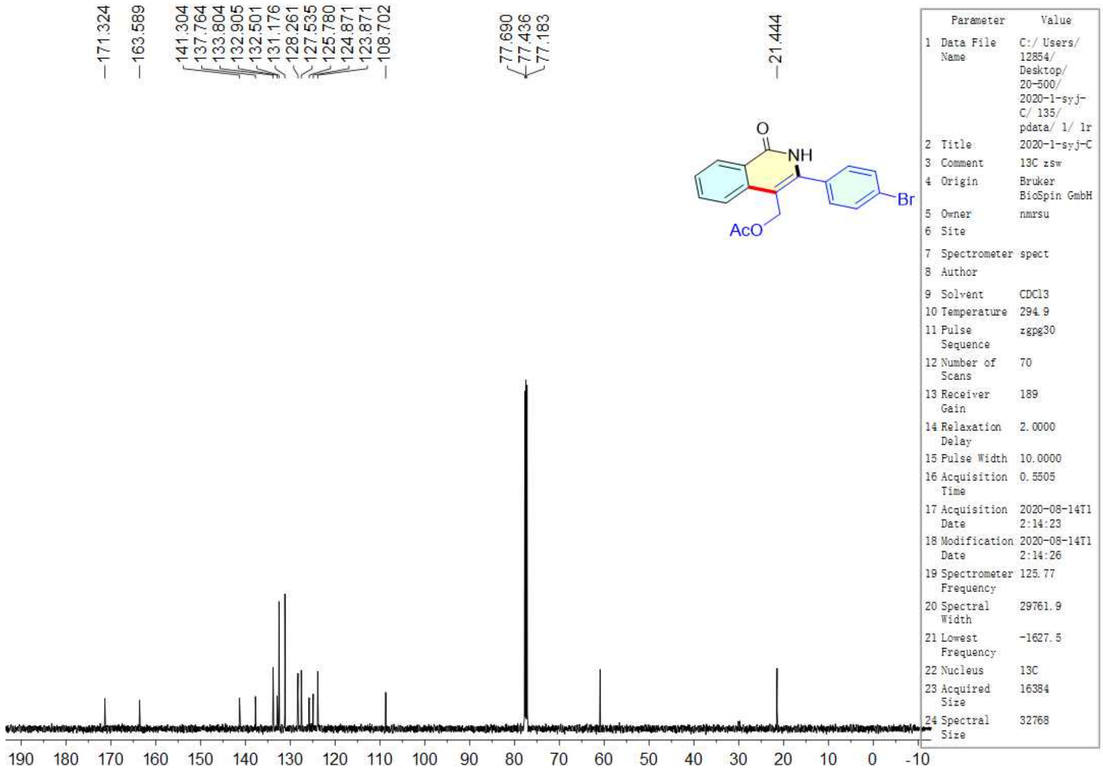

^{13}C NMR spectrum for compound **3ah**

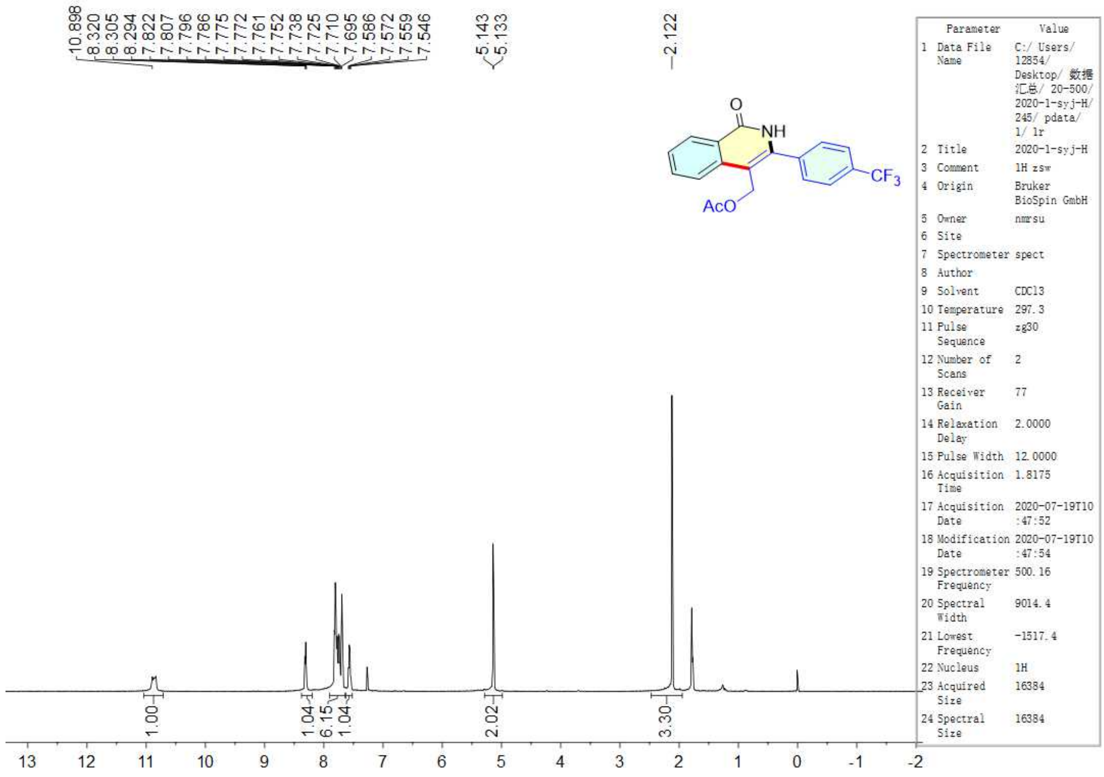

^{1}H NMR spectrum for compound **3ai**

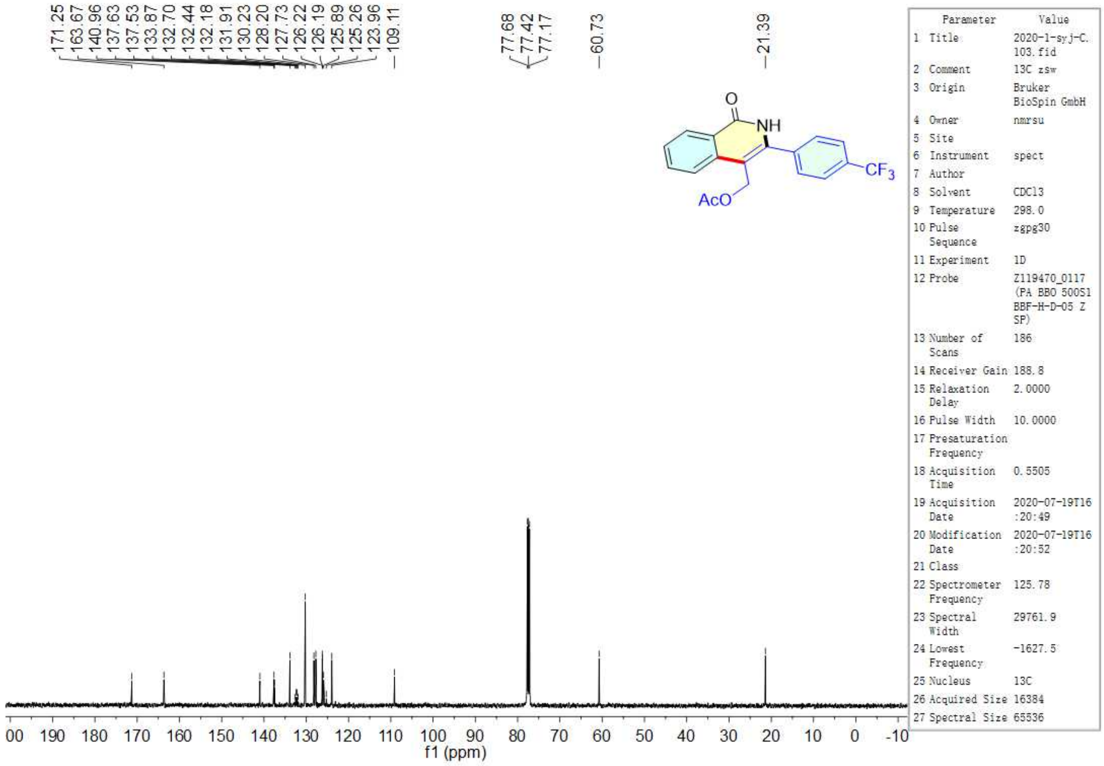

^{13}C NMR spectrum for compound **3ai**

2. X-ray crystal data of compound 3aa

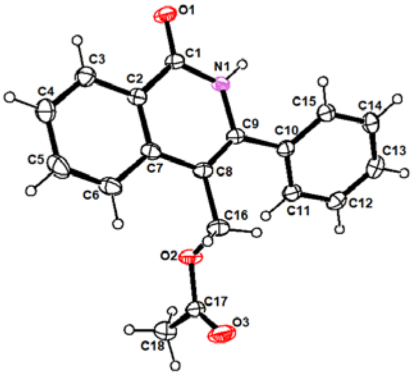

The purified compound **3aa** is dissolved in a mixed solvent of ethyl acetate and n-Hexane, and placed in a dark cabinet to slowly evaporate. After several days, a colourless bulk crystal was obtained. The X-ray crystal-structure determinations were obtained on a Bruker Smart CCDC APEX-2 diffractometer (graphite- monochromated Mo *Kα* radiation, λ=0.71073 nm) at 293.15 K.

Figure S1. ORTEP drawing of compound **3aa** (CCDC 2220211) (30% probability for the thermal ellipsoid).

Table S1. Crystal data and structure refinement for compound **3aa**

CCDC number	2220211	
Identification code	mo_20200722b_0m	
Empirical formula	C18 H15 N O3	
Formula weight	293.31	
Temperature	300.00 K	
Wavelength	0.71073 Å	
Crystal system	monoclinic	
Space group	$P2_1/c$	
Unit cell dimensions	a = 15.2877(12) Å	α= 90°.
	b = 5.3060(4) Å	β= 99.569(3)°.
	c = 18.1471(14) Å	γ= 90°.
Volume	1451.55(19) $Å^3$	
Z	4	
Density (calculated)	1.342 g/cm^3	
Absorption coefficient	0.092 mm^{-1}	
F(000)	616.0	
Crystal size	0.15 × 0.11 × 0.1 mm^3	
2Θ range for data collection	5.668 to 55.002°.	
Index ranges	-19<=h<=19, -6<=k<=6, -23<=l<=23	
Reflections collected	32608	
Independent reflections	3312 [R(int) = 0.0848, R(sigma) = 0.0381]	
Data/restraints/parameters	3312/0/200	
Goodness-of-fit on F^2	1.031	
Final R indices [I>2sigma(I)]	R1 = 0.0501, wR2 = 0.1091	
Final R indices (all data)	R1 = 0.0907, wR2 = 0.1278	
Largest diff. peak and hole	0.18 and -0.23 e.$Å^{-3}$	
